# Yolk sac cell atlas reveals multiorgan functions during human early development

**DOI:** 10.1126/science.add7564

**Published:** 2023-08-18

**Authors:** Issac Goh, Rachel A. Botting, Antony Rose, Simone Webb, Justin Engelbert, Yorick Gitton, Emily Stephenson, Mariana Quiroga Londoño, Michael Mather, Nicole Mende, Ivan Imaz-Rosshandler, Lu Yang, Dave Horsfall, Daniela Basurto-Lozada, Nana-Jane Chipampe, Victoria Rook, Jimmy Tsz Hang Lee, Mai-Linh Ton, Daniel Keitley, Pavel Mazin, M.S. Vijayabaskar, Rebecca Hannah, Laure Gambardella, Kile Green, Stephane Ballereau, Megumi Inoue, Elizabeth Tuck, Valentina Lorenzi, Kwasi Kwakwa, Clara Alsinet, Bayanne Olabi, Mohi Miah, Chloe Admane, Dorin-Mirel Popescu, Meghan Acres, David Dixon, Thomas Ness, Rowen Coulthard, Steven Lisgo, Deborah J Henderson, Emma Dann, Chenqu Suo, Sarah J. Kinston, Jong-eun Park, Krzysztof Polanski, John Marioni, Stijn van Dongen, Kerstin B. Meyer, Marella de Bruijn, James Palis, Sam Behjati, Elisa Laurenti, Nicola K. Wilson, Roser Vento-Tormo, Alain Chédotal, Omer Bayraktar, Irene Roberts, Laura Jardine, Berthold Göttgens, Sarah A. Teichmann, Muzlifah Haniffa

**Affiliations:** 1 Wellcome Sanger Institute, Wellcome Genome Campus, Hinxton, Cambridge CB10 1SA, UK; 2 Biosciences Institute, Newcastle University, NE2 4HH, UK; 3 Sorbonne Université, INSERM, CNRS, Institut de la Vision, Paris, France; 4 Department of Haematology, Wellcome-MRC Cambridge Stem Cell Institute, CB2 0AW, UK; 5 MRC Laboratory of Molecular Biology, Cambridge Biomedical Campus, CD2 0QH, UK; 6 Department of Zoology, University of Cambridge, Cambridge UK; 7 Translational and Clinical Research Institute, Newcastle University, NE2 4HH, UK; 8 Centre Nacional d’Analisi Genomica-Centre de Regulacio Genomica (CNAG-CRG), Barcelona Institute of Science and Technology (BIST), Barcelona, Spain; 9 NovoPath, Department of Pathology, Newcastle Hospitals NHS Foundation Trust, Newcastle upon Tyne, UK; 10 Korea Advanced Institute of Science and Technology, Daejeon, South Korea; 11 EMBL-EBI, Wellcome Genome Campus, Cambridge, UK; 12 CRUK Cambridge Institute, University of Cambridge, Cambridge, UK; 13 MRC Molecular Haematology Unit, MRC Weatherall Institute of Molecular Medicine, Radcliffe Department of Medicine, University of Oxford, OX3 9DS, UK; 14 Department of Pediatrics, University of Rochester Medical Center, Rochester, 14642, NY, USA; 15 Department of Paediatrics, University of Cambridge, Cambridge, UK; 16 Department of Paediatrics, University of Oxford, OX3 9DS, UK; 17 Theory of Condensed Matter Group, Cavendish Laboratory/Department of Physics, University of Cambridge, Cambridge, UK; 18 Department of Dermatology and NIHR Newcastle Biomedical Research Centre, Newcastle Hospitals NHS Foundation Trust, Newcastle upon Tyne, NE1 4LP, UK

## Abstract

The extraembryonic yolk sac (YS) ensures delivery of nutritional support and oxygen to the developing embryo but remains ill-defined in humans. We therefore assembled a comprehensive multiomic reference of human YS from 3-8 post-conception weeks by integrating single-cell protein and gene expression data. Beyond its recognized role as a site of hematopoiesis, we highlight roles in metabolism, coagulation, vascular development, and hematopoietic regulation. We reconstructed the emergence and decline of YS hematopoietic stem/progenitor cells from hemogenic endothelium and revealed a YS-specific accelerated route to macrophage production that seeds developing organs. The multiorgan functions of YS are superseded as intraembryonic organs develop, effecting a multifaceted relay of vital functions as pregnancy proceeds.

## Structured Abstract

**Introduction:**

The yolk sac (YS) generates the first blood and immune cells and provides nutritional and metabolic support to the developing embryo. Our current understanding of its functions derives from pivotal studies in model systems and insights from human studies are limited. Single-cell genomics technologies have facilitated the interrogation of human developmental tissues at unprecedented resolution. Atlases of blood and immune cells from multiple organs have been greatly enhanced by focused, time-resolved analyses of specific tissues.

**Rationale:**

To characterize the functions of human YS, we performed single-cell RNA sequencing (scRNA-seq) and cellular indexing of transcriptomes and epitopes (CITE-seq) on YS and paired embryonic liver. After integration with external datasets, our reference comprised 169,798 cells from 10 samples spanning 4-8 post-conception weeks (PCW) or Carnegie stages (CS) 10-23. A repertoire of 2D and 3D imaging techniques provided spatial context and validation. We compared the products of two hematopoietic inducible pluripotent stem cell (iPSC) culture protocols against our reference.

**Results:**

We determined that YS metabolic and nutritional support originates in the endoderm and that endoderm produces coagulation proteins and hematopoietic growth factors (erythropoietin (EPO) and thrombopoietin (THPO)). Although metabolic and coagulation protein production was conserved between humans, mice, and rabbits, EPO and THPO production was observed in humans and rabbits only.

We reconstructed trajectories from YS hemogenic endothelium to early hematopoietic stem and progenitor cells (HSPCs). Using transcriptomic signatures of early and definitive hematopoiesis, we parsed YS HSPCs into myeloid-biased early HSPCs, and lymphoid and megakaryocyte-biased definitive HSPCs. Human embryonic liver remained macroscopically pale prior to CS14, when hematopoietic cells first emerge from the aorta–gonad–mesonephros (AGM) region. Tracking hemoglobin subtypes led us to conclude that initial erythropoiesis is YS-restricted. In mice by contrast, Hb subtypes suggested two waves of pre-AGM erythropoiesis, including maturation in the macroscopically red embryonic liver.

Before CS14, monocytes were absent and macrophages originated from HPSCs via a pre-macrophage cell state. After CS14, monocytes emerged and a second, monocyte-dependent differentiation trajectory was reconstructed. A rare subset of TREM2^+^ macrophages, with a microglia-like transcriptomic signature, was present after CS14. The iPSC system optimized for macrophage production recapitulated the two routes to macrophage differentiation but did not generate the diversity of macrophages (including TREM2^+^ macrophages) observed in developing tissues.

**Conclusions:**

Our study illuminates a previously obscure phase of human development, where vital functions are delivered by the YS acting as a transient extraembryonic organ. Our comprehensive single cell atlas represents a valuable resource for studying the cellular differentiation pathways unique to early life and leveraging these for tissue engineering and cellular therapy.

**Figure:**
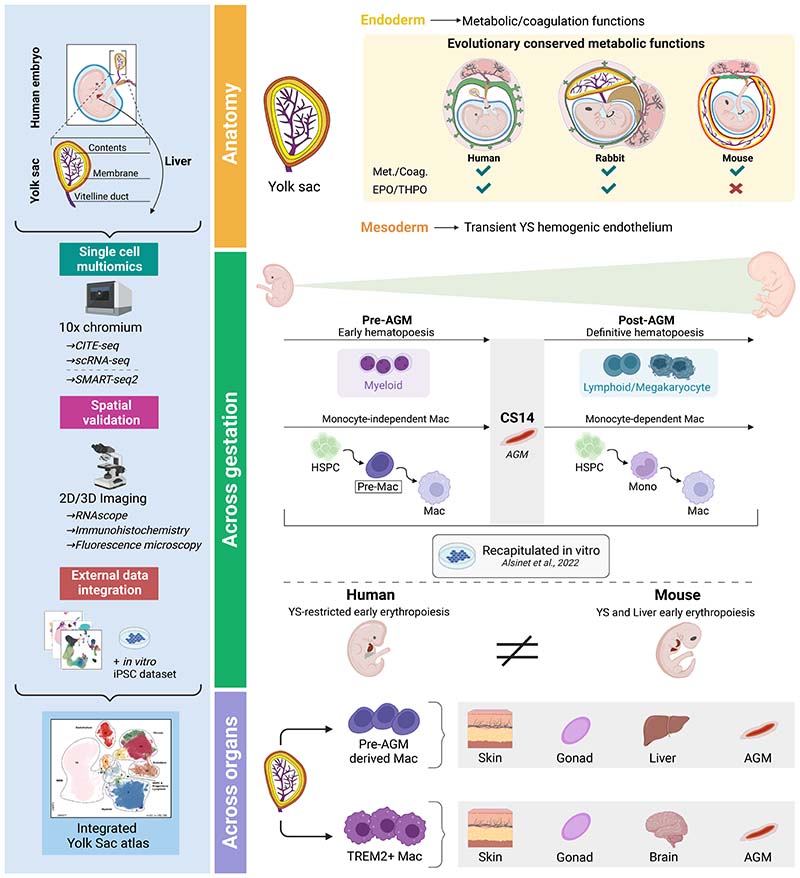
Multiorgan functions of the human yolk sac We characterized functions of the developing human YS, combining scRNA-seq and CITE-seq, with 2D and 3D imaging techniques. Our findings revealed YS contributions to metabolic and nutritional support, and early hematopoiesis. We characterized myeloid bias in early hematopoiesis, distinct myeloid differentiation trajectories, evolutionary divergence in initial erythropoiesis, and YS contributions to developing tissue macrophages.

The primary human yolk sac (YS) derives from the hypoblast at the time of embryo implantation (Carnegie stage 4, (CS4); ˜1 post conception week (PCW)) (1, 2). The secondary YS supersedes the primary structure at around CS6 (˜2.5 PCW) and persists until CS23 (˜8 PCW) (1, 2). The secondary YS has three tissue compartments: mesothelium (an epithelial layer interfacing the amniotic fluid), mesoderm (including endothelial cells, blood cells, and smooth muscle), and endoderm (an inner layer interfacing the vitelline-fluid-filled YS cavity) (1). The functions of the YS in nutrient uptake, transport, and metabolism are phylogenetically conserved (2).

Hematopoiesis originates in the YS of mammals, birds, and some ray-finned fishes (3). The first wave of mouse YS hematopoiesis yields primitive erythroid cells, macrophages, and megakaryocytes (MKs) from embryonic day 7.5 (E7.5) (3, 4). After circulation begins, a second wave of erythromyeloid and lymphomyeloid progenitors arise in the YS and supply the embryo (5). Finally, definitive hematopoietic stem cells arise in the aorta–gonad–mesonephros (AGM) region of the dorsal aorta and seed the fetal liver (6). Limited evidence suggests that the YS also provides the first blood cells during human development. Primitive erythroblasts expressing embryonic globin genes, surrounded by endothelium, emerge in the YS at CS6 (˜2.5 PCW) (7, 8). Hematopoietic progenitors and macrophages are detectable at CS11 (˜4 PCW) (9), with MKs, monocytes, mast cells and innate lymphocytes also reported (9, 10). Long-term multilineage repopulating (definitive) hematopoietic stem and progenitor cells (HSPCs) originate in the AGM at CS14 (˜5 PCW) (11). Equivalent cells are subsequently found in the YS at CS16 and the liver from CS17 (11, 12).

In this study, we report a time-resolved atlas of the human YS, combining single-cell protein and gene expression with imaging, providing a comprehensive depiction of the metabolic and hematopoietic functions of the human YS, as well as a benchmark for in vitro culture systems aiming to recapitulate human early development.

## A single-cell atlas of human YS

**Fig. 1:**
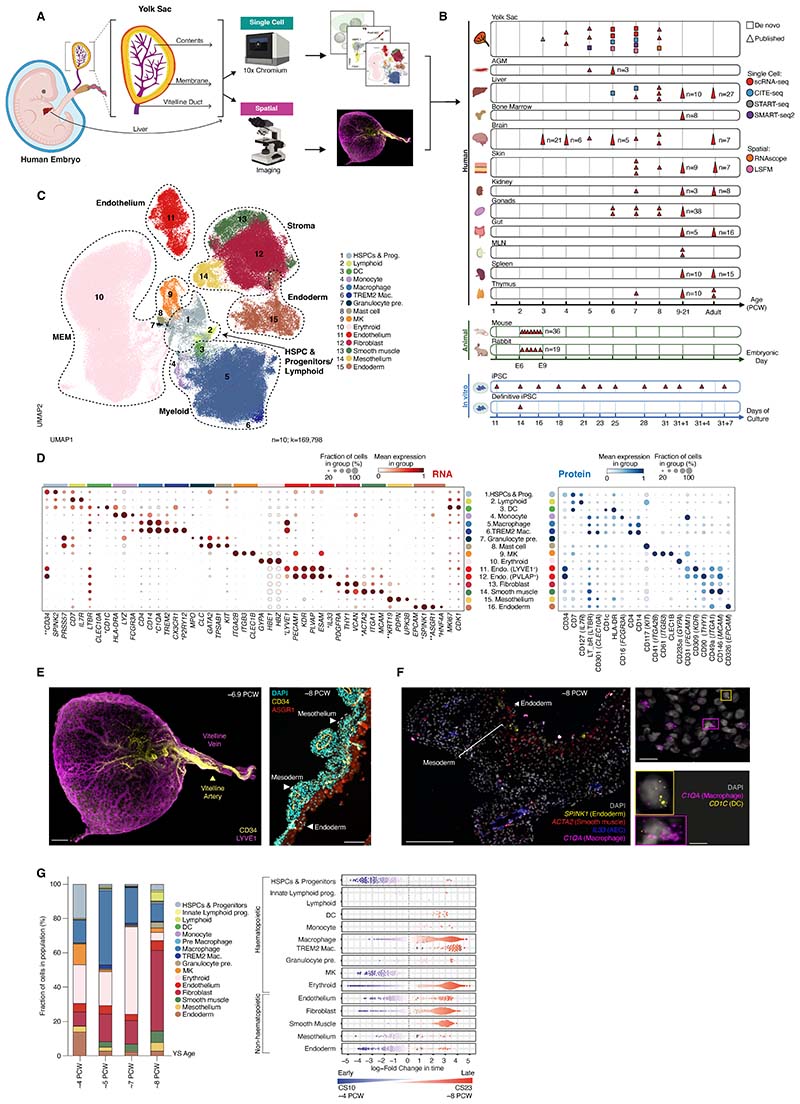
A single-cell atlas of the human yolk sac. **(A)** Schematic of experimental outline. **(B)** Summary of data included in analyses. Squares represent new data and triangles represent published data: YS (10, 12, 49, 64), AGM (12), liver (10), fetal BM (35), fetal brain (56), fetal skin (49), fetal kidney (80), fetal gonads (50), mouse (75), iPSC (12, 20). Color indicates assay used (data S6). (**C)** UMAP visualization of YS scRNA-seq data (n=10; k=169,798), colors represent broad cell states: DC: dendritic cell, Mac: macrophage, MEM: megakaryocyte—erythroid–mast cell lineage, MK: megakaryocyte, pre.: precursor. (**D**) Left: Dot plot showing the mean expression (color) and proportion of cells expressing genes (dot size) of broad cell states in YS scRNA-seq data. Right: Equivalent protein expression (color) and proportion of cells expressing proteins (dot size) from YS CITE-seq data (n=2; k=3,578). Equivalent gene names are in parentheses. * indicates genes validated by RNAscope and ** indicates proteins validated by IHC/IF (data S4). Data are variance-scaled and min-max-standardized. (**E**) Left: light-sheet fluorescence microscopy of CD34^+^ and LYVE1^+^ vascular structures in YS (representative ˜6.9 PCW sample; scale bar: 500 µm; movie S1). Right: Immunofluorescence of an ˜8 PCW YS highlighting endoderm (ASGR1; red) and endothelium (CD34; yellow), costained with DAPI (cyan). Scale bar: 100 µm (data S23). (**F**) RNAscope of YS (representative 8 PCW sample). Left: endoderm (*SPINK1;* yellow), smooth muscle (*ACTA2;* red), AEC (*IL33;* blue), and macrophages (*C1QA;* magenta) (scale bar: 200 µm). Right: DCs (*CD1C*; yellow box) and macrophages (*C1QA*; magenta box) (scale bar: 50 µm). Individual channels shown in fig. S4A. (**G**) Left: Bar graph showing the proportion representation of cell states in YS scRNA-seq data by gestational age. Right: Milo beeswarm plot of YS scRNA-seq neighborhood differential abundance across time. Blue/red neighborhoods are significantly enriched earlier/later in gestation respectively. Color intensity denotes degree of significance (data S24).

We performed droplet-based single cell RNA-sequencing (scRNA-seq) to profile human YS, and integrated with external datasets to yield 169,798 high-quality cells from 10 samples spanning 4-8 PCW (CS10-CS23), which can be interrogated on our interactive web portal (https://developmental.cellatlas.io/yolk-sac (13)) (Fig. 1, A to C; fig. S1, A to C; and data S1 to S18). Graph-based Leiden clustering yielded 39 cell types grouped into 15 broad categories including hematopoietic cells, endoderm, mesoderm, and mesothelium. Key marker genes were validated by plate-based scRNA-seq (Smart-seq2) (Fig. 1, C and D; fig. S1, B to F; and data S3 to S5, S8, S17, and S18). We used the term “HSPC” for cells collectively based on their expression of a core HSPC signature (e.g., *CD34, SPINK2*, and *HLF*) without implying long-term repopulating capacity or multilineage potential. With comparison datasets, unless otherwise specified, we adopted published annotations (data S6 and S7). Surface protein expression from CITE-seq of n=2 YS cell suspensions (fig. S1, G and H) identified combinatorial antigens for cell purification and functional characterization (fig. S2A and data S9, S19, and S20). We generated matched embryonic liver scRNA-seq and CITE-seq data (figs. S2, B to F, and S3, A to C, and S5, S10, S21, and S22), which confirmed the presence of discrete B cell progenitor stages only in the liver (fig. S3C and data S12). Around half of YS lymphoid cells were innate lymphoid progenitors (I. Lymp. Prog.), which terminated in natural killer (NK) and innate lymphoid cell (ILC) precursor states on force-directed graph (FDG) visualization (fig. S3D). A small population of cells were termed “Lymphoid B lineage” due to their expression of *CD19, CD79B*, and *IGLL1*. These cells did not express the typical B1 markers *CD5, CD27*, or *CCR10* however. Given the absence of distinct B cell progenitor stages and their later emergence (>5 PCW), these may constitute migratory B cells of fetal liver origin (fig. S3, D and E).

Three-dimensional visualization of the YS by light sheet microscopy marked the CD34^hi^LYVE1^lo^ vitelline artery and CD34^lo^LYVE1^hi^ vitelline vein contiguous with a branching network of CD34^lo^LYVE1^hi^ vessels (Fig. 1E; fig. S3F; data S23; and movies S1 and S2). The CD34^lo^LYVE1^hi^*IL33*^+^ vessels were situated within the mesoderm, a distinct layer beneath the ASGR1^+^*SPINK1*^+^ endoderm (Fig. 1, E and F, and figs. S3, F to I, and S4, A and B). *ACTA2*^+^ smooth muscle cells formed a sublayer between mesoderm and endoderm (Fig. 1F and fig. S4, A and B). Macrophages (*C1QA*^+^*CD1C*^+/−^ and a small number of dendritic cells (DCs) (*C1QA*^+/−^*CD1C*^+^) were identified within the mesoderm (Fig. 1F and fig. S4A).

The most prevalent hematopoietic cell types in early YS (CS10; ˜4 PCW) were HSPCs, erythroid cells, macrophages, and megakaryocytes. Both HSPCs and MKs proportionately diminished thereafter, whereas erythroid cells and macrophages were sustained. DCs and *TREM2*^+^ macrophages did not emerge until >6 PCW (Fig. 1G and data S4). The ratio of hematopoietic to nonhematopoietic cells was around 3:1 in early YS (CS10; ˜4 PCW), with endoderm relatively abundant (Fig. 1G). The ratio approached 1:3 in late YS (CS22-23; ˜8 PCW) due to expansion of fibroblasts (Fig. 1G). The transcriptional profile of MKs was consistent across gestation, but both erythroid cells and macrophages had early and late gestation-specific molecular states, suggesting dual waves of production (Fig. 1G; fig. S4C; and data S24).

## Multiorgan functions of YS

**Fig. 2:**
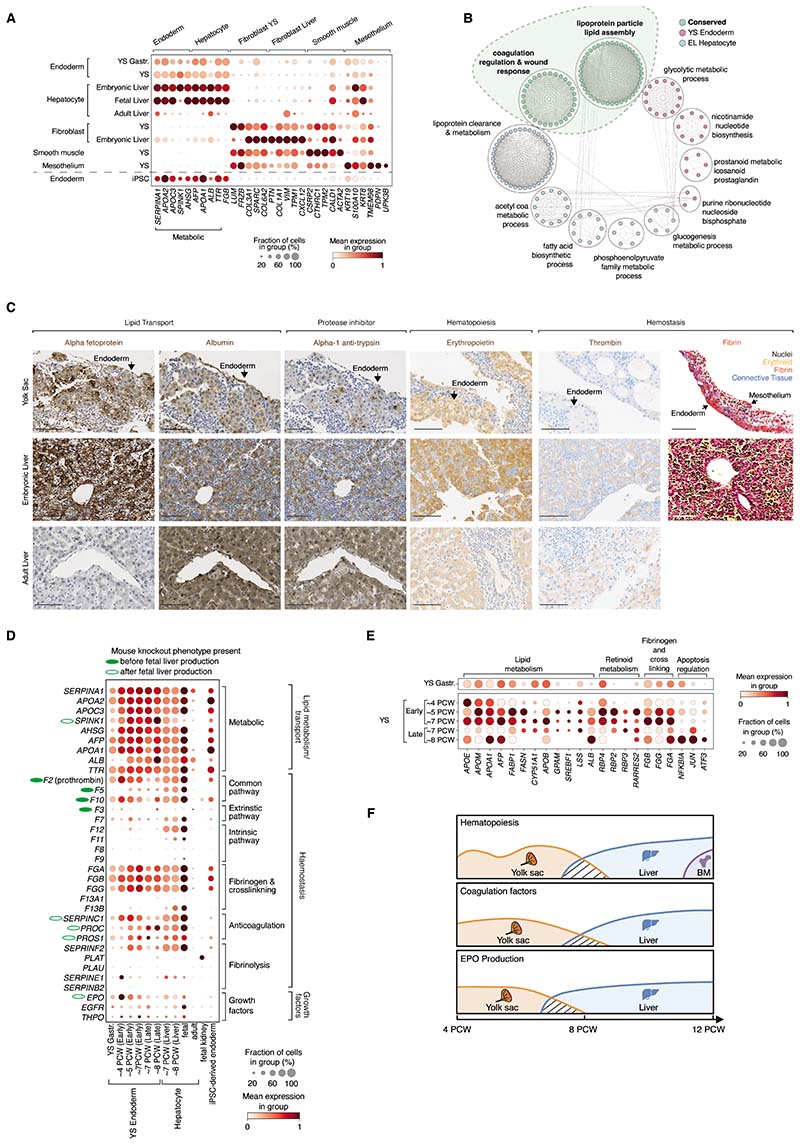
Multiorgan functions of YS. **(A)** Dot plot showing the mean expression (color) and proportion of YS and liver stromal cells (dot size) expressing stromal DEG markers (data S3, S7, and S21). YS scRNA-seq data includes main and gastrulation (gastr.) data. Liver scRNAseq data includes matched embryonic, fetal, and adult liver. **(B)** Flower plot illustrating significantly enriched pathways in YS endoderm (pink) and embryonic liver (EL) hepatocytes (blue). Conserved pathways between tissues are highlighted in green and a dashed outline (data S25). **(C)** Columns 1-3: IHC staining of alpha fetoprotein (*AFP*), albumin (*ALB*) and alpha-1 antitrypsin (*SERPINA1*) in 8 PCW YS and EL (middle), and adult liver (bottom). Representative images of n=5 YS (4-8 PCW), n=3 ELs (7-8 PCW) and n=3 adult liver samples. Columns 4-5: IHC staining of erythropoietin (EPO) and thrombin (F2) in 7 PCW YS (top), 7 PCW EL (middle), and healthy adult liver (bottom). Representative images from n=3 samples per tissue: YS (4-7 PCW), ELs (7-12 PCW). Protein (brown) and nuclei (blue). Column 6: Martius Scarlet Blue (MSB)-stained 8 PCW EL (representative of n=3) and 4 PCW YS (representative of n=3). Nuclei (gray), erythroid (yellow), fibrin (red), and connective tissue (blue) (data S23). Scale bars: 100 μm. **(D)** Dot plot showing the mean expression (color) and proportion of cells in YS endoderm, embryonic, fetal and adult liver hepatocytes, and stromal cells from fetal kidney (64). Brackets indicate enriched GO annotations. Green ellipses denote genes with prenatal phenotypes in homozygous null mice. Solid/hollow green outline denotes phenotype onset prior/post fetal liver function respectively, as per fig. S4E (data S25) **(E)** Dot plot showing the mean expression (color) and proportion of cells expressing Milo-derived DEGs across gestation (dot size) in YS endoderm (data S24). Genes are grouped by function. **(F)** Schematic of the relative contributions of YS (orange), liver (blue), and BM (purple) to hematopoiesis, coagulation factor, and EPO production in the first trimester of human development.

YS endoderm coexpressing *APOA1/2, APOC3*, and *TTR*, (similar to embryonic/fetal hepatocytes) was present from gastrulation at ˜2-3 PCW (14) (Fig. 2A and data S3, S7, and S21). YS endoderm expressed higher levels of serine protease 3 (*PRSS3*), glutathione S-transferase alpha 2 (*GSTA2*), and multi-functional protein galectin 3 (*LGALS3*), compared to embryonic liver hepatocytes, whereas hepatocytes expressed a more extensive repertoire of detoxification enzymes, including alcohol and aldehyde dehydrogenases and cytochrome P450 enzymes (fig. S4D and data S3, S7, and S21). Both cell states shared gene modules implicated in coagulation and lipid and glucose metabolism (Fig. 2B and data S25), which were also conserved in mouse and rabbit extraembryonic endoderm (fig. S4, E and F, and data S25). The expression of transport proteins (alpha-fetoprotein and albumin), a protease inhibitor (alpha-1-antitrypsin), erythropoietin (EPO), and coagulation proteins (thrombin, prothrombin, and fibrin) were validated in human YS endoderm and embryonic liver hepatocytes (Fig. 2C and fig. S4G).

From the earliest timepoints, YS endoderm expressed genes for anticoagulant proteins antithrombin III (*SERPINC1*) and protein S (*PROS1*) and components of the tissue factor-activated extrinsic coagulation pathway—thrombin (*F2*), factor VII (*F7*), and factor X *(F10)* (Fig. 2D and data S25), confirmed at the protein level for thrombin (Fig. 2C). Intrinsic pathway factors VIII, IX, XI, and XII (*F8, F9, F11*, and *F12*) were minimally expressed in YS, but were expressed by embryonic liver hepatocytes (Fig. 2D). Tissue factor, antithrombin III and fibrinogen subunits were also expressed in mouse extraembryonic endoderm and rabbit YS endoderm (fig. S4E). Embryonic lethality of homozygous-null mice lacking prothrombin, thrombin, and coagulation factor V prior to liver synthetic function (i.e., at E9-12) implies functional relevance of YS expression (Fig. 2D and data S25) (15, 16), whereby coagulant and anticoagulant pathways develop in parallel to balance hemostasis.

YS endoderm cells expressed EPO and THPO that are critical for erythropoiesis and megakaryopoiesis (Fig. 2, C and D; fig. S4H; and data S25 and S26). In mouse development, EPO is produced by fetal liver and is only essential for definitive and the later stages of primitive erythropoiesis, with *Epo*/*Epor*-knockout mice dying at around E13 (17). An EPO source prior to liver development is therefore likely not needed in mice. Accordingly, EPO has not been found in mouse YS (18) (fig. S4E). In parallel to human YS, rabbit YS endoderm also produced EPO at gestational stages preceding liver development (fig. S4E). We compiled a 12-organ integrated human fetal atlas spanning 3-19 PCW (k=3.12 million, n=150; fig. S8, G and H, and data S6 and S7) and observed that EPO and THPO production were restricted to YS and liver (fig. S4H), specifically to YS endoderm and liver hepatocytes (fig. S4I). Differentially expressed genes between early and late YS endoderm revealed active retinoic acid and lipid metabolic processes until 7 PCW, after which genes associated with cell stress and death were expressed (Fig. 2E and data S26). A decline in the proportion of YS endoderm cells producing EPO was compensated by onset of EPO production by hepatocytes at 7 PCW (fig. S4J).

Thus, the human YS plays a critical role supporting hematopoiesis, metabolism, coagulation, and erythroid cell mass regulation before these functions are taken over by the embryonic/fetal liver, and then ultimately, by the adult liver (metabolism and coagulation), bone marrow (BM) (hematopoiesis), and kidney (erythroid cell mass regulation) (Fig. 2F).

## Early versus definitive hematopoiesis in YS and liver

**Fig. 3:**
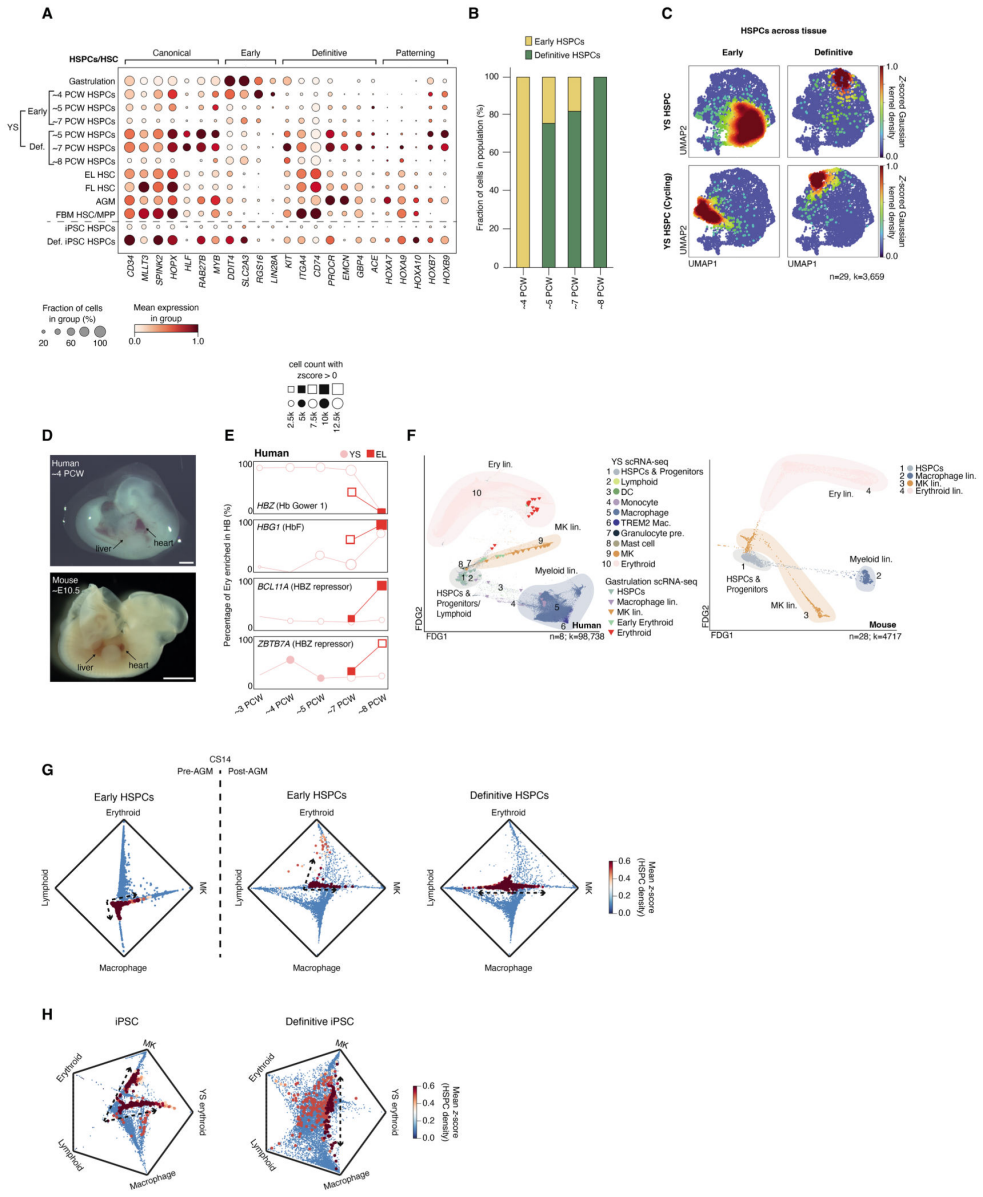
Early versus definitive hematopoiesis in YS and liver. **(A)** Dot plot showing mean expression (color) and proportion of cells expressing selected HSPC genes (dot size) in HSPCs from YS (main and gastrulation (14)), liver (EL and fetal/FL (10)), AGM (76), BM (35) and iPSC cultures (iPSC (20) and definitive iPSC (12)). **(B)** Bar chart showing proportion of early (yellow) to definitive HSPCs (green) in the YS scRNA-seq data grouped by gestational age. **(C)** Density plots showing YS HSPC (top) and cycling HSPC (bottom) with early (left) and definitive signatures (right) in an integrated landscape as per **A**. Color: population *z*-scored KDE (data S5). Tissue contributions are shown in fig. S5E. **(D)** Representative image of whole ˜4 PCW/CS12 human (top; n=4) and ˜E10.5/CS12 mouse embryo (bottom). Scale bars: 1 mm. **(E)** Line graphs showing change in erythroid cell proportion (*y*-axis) enriched in globin gene expression across gestational age. Colors indicate scRNA-seq dataset: Pink: human YS; Red: matched EL. Shape size: cell count; scale: representative counts; No shape: count <500. Globins grouped by roles in early or definitive hematopoiesis, and repression. **(F)** FDG of hematopoietic cell states in the YS scRNA-seq data (n=8, k=98,738; dots) integrated with human gastrulation (14) scRNA-seq data (n=1, k=91; triangles) (left), and equivalent cell states in the mouse gastrulation scRNA-seq dataset (75) (n=28, k=4,717; dots) (right). Colors represent cell states and clouds mark lineages. **(G)** Radial plots showing lineage transition probabilities between pre-AGM (CS10-11; left) and post-AGM (>CS14; right) YS early and definitive HSPCs. Color: population *z*-scored KDE. Density position indicates respective lineage priming probability between macrophage, lymphoid (NK and B lineage), erythroid, and MK terminal states. Arrows indicate proposed lineage priming based on KDE. **(H)** Radial plots showing lineage transition probabilities between iPSC-derived HSPCs (left) and definitive iPSC-derived HSPCs (right). Interpretation as in **G,** with addition of embryonic erythroid terminal state.

Human YS hematopoietic progenitors spanned two groups: HSPCs characterized by *SPINK2, CYTL1*, and *HOXB9* expression and cycling HSPCs characterized by cell cycle-associated genes such as *MKI67* and *TOP2A* (fig. S5A and data S17). Using markers recently associated with early (*DDIT4, SLC2A3, RGS16*, and *LIN28A*) and definitive (*KIT, ITGA4, CD74*, and *PROCR*) HSPCs (12), we identified early and definitive fractions within both HSPCs and cycling HSPCs (Fig. 3, A to C). Early and definitive HSPCs expressed canonical HSPC genes such as *SPINK2, HOPX*, and *HLF* (Fig. 3A and data S17), but diverged in expression of genes involved in multiple processes such as enzymes (*GAD1*), growth factors (*FGF23*), adhesion molecules (*SELL*), and patterning genes (*HOXA7*) (fig. S5C and data S17). By logistic regression (LR), YS HSPCs had a high median probability of class correspondence to liver HSCs, but this probability was higher for definitive than for early HSPCs (fig. S5B). YS cycling HSPCs had a bipartite probability distribution between liver MPP and CMP, with the definitive cycling HSPC more “MPP-like” than the early version. Differential protein expression in YS CITE-seq data indicated that CD122, CD194 (CCR4), and CD357 mark early HSPCs whereas CD44, CD48, CD93, and CD197 (CCR7) mark definitive HSPCs (fig. S5D and data S27), in keeping with the reported use of CD34 and CD44 to segregate early and definitive-type HSPCs by FACS (19). We confirmed that an iPSC-derived culture system reported to generate definitive HSPCs did express RNA markers characteristic of definitive HSPCs (12), but an iPSC-derived culture system optimized for macrophage production (20) did not (Fig. 3A).

To assess cross tissue HSPC heterogeneity, we integrated HSPCs across hematopoietic organs (Fig. 3C and fig. S5E and data S6 and S7). By kernel density estimation score (KDE) on integrated UMAP embeddings, YS definitive HSPCs qualitatively colocalized with definitive HSPCs from age-matched liver (Fig. 3C and fig. S5E). From exclusively early HSPCs at ˜3 PCW, we observed rapid accumulation of definitive HSPCs after AGM development CS14 (˜5 PCW), likely accounting for the increase in the YS HSPC/progenitor fraction at 8 PCW (Figs. 1G and 3B and fig. S5F).

Next, we examined the transition from YS to liver hematopoiesis. Prior to AGM, the human embryonic liver is macroscopically pale, suggesting that erythropoiesis predominantly occurs in the YS (Fig. 3D). We tracked the proportional representation of hemoglobin (Hb) subtypes over time as a proxy for YS versus embryonic liver contributions. *HBZ* and *HBE1* (genes for Hb Gower 1) were restricted to YS erythroblasts, whereas *HBG1* (which forms fetal Hb/HbF in combination with an alpha chain) was expressed in fetal liver erythroblasts (21–26) (Fig. 3E and fig. S5H). The sustained *HBZ* production in YS for several days prior to liver bud formation (4 PCW) was consistent with a scenario where YS supports initial erythropoiesis. At 7 PCW, the embryonic liver contained both *HBZ* and *HBG1* (Fig. 3E), in keeping with previous studies of Hb switching (8). By 8 PCW, embryonic liver erythroblasts expressed *HBZ-*repressors and were *HBG1-*dominant, as we have previously shown (10). By contrast, the mouse liver was macroscopically red prior to AGM maturation (Fig. 3D). Tracking Hb subtype usage in the mouse, we noted two waves of pre-AGM erythropoiesis: an initial wave with *Hbb-y* and *Hba-*x*-Hba-a1/2*, and a second wave mirrored in both YS and torso/liver (*Hbb*-*bt1* and *Hbb-bs*) (fig. S5, G to I). Thus, there is a species-specific difference in YS erythropoiesis, and a rapid shift in Hb usage following AGM development in humans.

We examined data from human gastrulation (˜2-3 PCW) and CS10-11 (˜4 PCW)—timepoints prior to AGM-HSPC formation—to explore the differentiation potential of early HSPCs. At gastrulation, the YS hematopoietic landscape had a tripartite differentiation structure, with erythroid, MK, and myeloid differentiation (Fig. 3F). This structure was also observed in mouse YS (fig. S6, A and B, and data S5 and S11). Differential-fate-prediction analysis demonstrated that early HSPCs pre-AGM at CS10-11 (˜4 PCW) were myeloid-biased, consistent with previous observations (9). However, the abundance of differentiating erythroid and MK cells at CS10-11 suggested that an earlier wave of erythroid/MK production had occurred (Fig. 3G and fig. S6C). Post-AGM, the model predicted that remaining early HSPCs were erythroid and MK-biased, whereas definitive HSPCs were lymphoid- and MK-biased (Fig. 3G). This was in keeping with the first appearance of YS lymphoid cells (ILC progenitors, NK cells, and B lineage cells) post CS14 (Fig. 1G). Differential-fate-prediction analyses suggested that iPSC-derived HSPCs were embryonic erythroid-, myeloid-, and MK-biased, whereas definitive iPSC-derived HSPCs were lymphoid-, MK-, erythroid-, and myeloid-primed, consistent with the predicted lineage potential of their in vivo early and definitive counterparts (Fig. 3H and fig. S6D).

## The lifespan of YS HSPCs

**Fig. 4:**
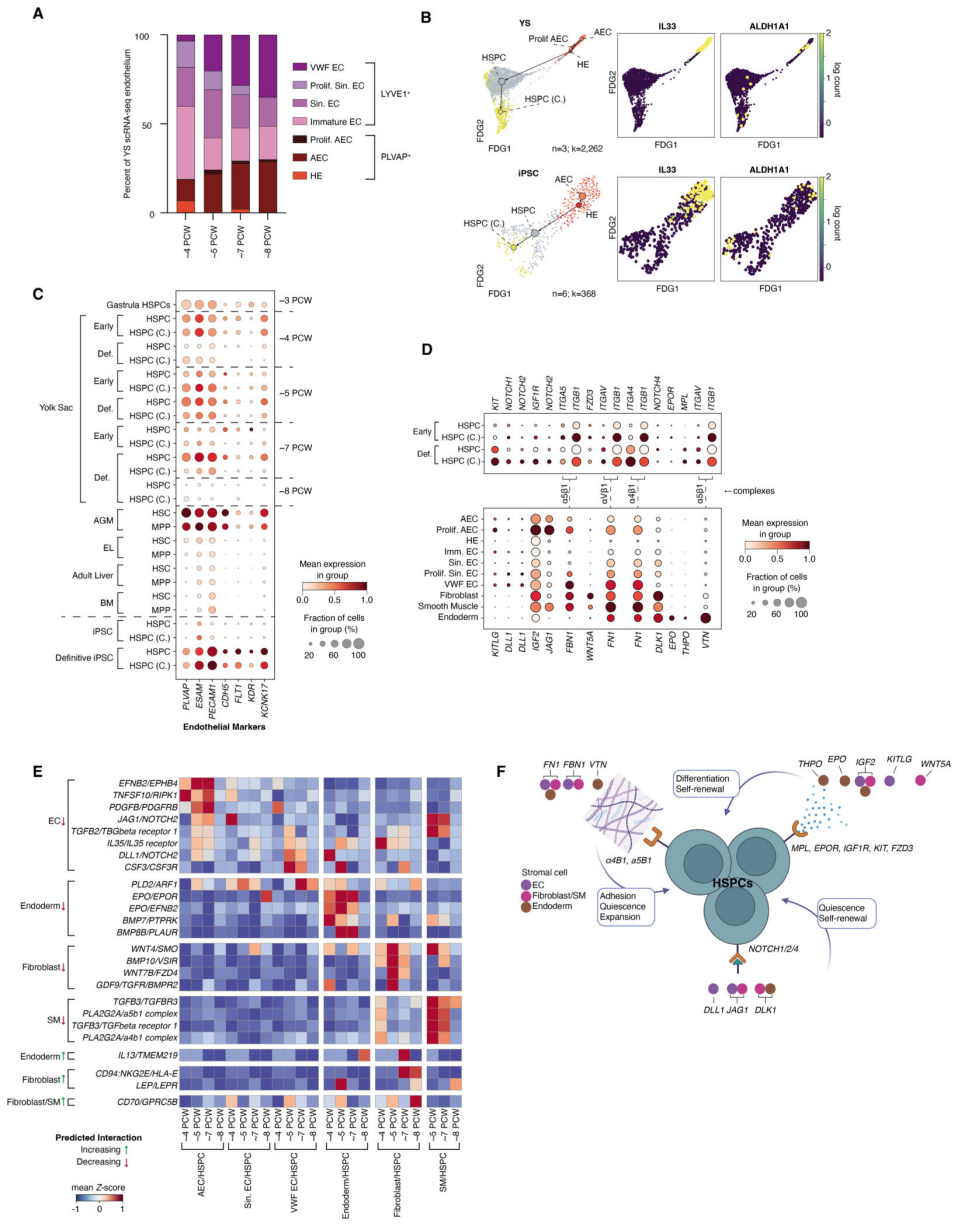
The lifespan of YS HSPCs. **(A)** Bar chart showing the relative proportions of YS endothelial cell (EC) subsets by age (PCW). EC: endothelial cells, AEC: arterial endothelial cells, Sin. EC: sinusoidal endothelial cells, and HE: hemogenic endothelium. **(B)** FDG overlaid with PAGA showing trajectory of HE transition to HSPC in YS scRNA-seq data (n=3; CS10, 11 and 14; k=2,262) (top) and iPSC-derived HSPC scRNA-seq data (n=7, k=437) (20) (bottom), with feature plots of key genes (*IL33, ALDH1A1*) involved in endothelial to hemogenic transition (data S5). **(C)** Dot plot showing the mean expression (color scale) and proportion of cells expressing EC-associated genes (dot size) in HSPCs across gestational age (PCW). HSPCs are derived from YS (including gastrulation), AGM (12), matched EL (embryonic liver), FL (fetal liver) (10), fetal BM (35), iPSC-derived HSPC (20) and definitive iPSC-derived HSPC (12) scRNA-seq datasets. (**D)** Dot plot of the mean expression (color scale) and the fraction of cells expressing each gene (dot size) of curated genes predicted by CellphoneDB to form statistically significant (*P*<0.05) protein–protein interactions between HSPCs (top plot) and stromal cells (bottom plot) across all time gestational points. Brackets indicate which protein counterparts form complexes (data S29). Data are log-normalized, variance-scaled, and min–max-standardized with a distribution of 0-1. **(E)** Heatmap showing curated and statistically significant (*P*<0.05) CellphoneDB-predicted interactions between YS HSPCs and stromal cells that change across gestation. Color scale indicates relative mean expression *z*-scores. **(F)** Schematic of selected and statistically significant (*P*<0.05) CellphoneDB-predicted interactions between YS HSPCs and endoderm, fibroblasts (Fib), smooth muscle cells (SMC), or EC derived from scRNA-seq data. Interactions are grouped by predicted receptor to ECM interactions, ligand—receptor interactions, and surface-bound ligand–receptor interactions. Receptors and ligands in italics significantly decrease at CS17-23 (6-8 PCW) (data S28 and S29).

HSPCs arise from hemogenic endothelium (HE) in the aorta, YS, BM, placenta, and embryonic head in mice (27–31). In human AGM, definitive HSPCs emerge from *IL33*^+^*ALDH1A1*^+^ arterial endothelial cells (AECs) via *KCNK17*^+^*ALDH1A1*^+^ HE (32). Dissecting YS endothelial cell (EC) states in greater detail, the broad category of PLVAP^+^ ECs included AECs and HE, whereas LYVE1^+^ ECs encompassed sinusoidal, immature, and VWF-expressing ECs (Fig. 4A; fig. S7, A and B, and data S4 and S5). HE was a transient feature of early YS (Fig. 4A). Along inferred trajectories, YS HSPCs appeared to arise from AECs via HE as in AGM (12), sequentially upregulating expected genes such as *ALDH1A1 (33)* (Fig. 4B). The same EC intermediate states and transition points were identified in both iPSC culture systems (Fig. 4B and fig. S7C). In keeping with their more recent endothelial origin, we found that YS HSPCs and AGM HSPCs, but not embryonic liver or fetal BM HSPCs retained an EC gene signature characterized by the expression of *KDR, CDH5, ESAM*, and *PLVAP* (Fig. 4C).

Receptor–ligand interactions capable of supporting HSPC expansion and maintenance in YS were predicted using CellPhoneDB (34) and compared to predictions in fetal BM (35). We identified YS ECs, fibroblasts, smooth muscle cells, and endoderm as likely interacting partners (Fig. 4, D to F, and data S28). YS ECs, like fetal BM ECs, were predicted to maintain and support the HSPC pool (36) through the production of stem cell factor (*KITLG*) and *NOTCH1/2*, although the repertoire of NOTCH ligands diverged between tissues (*DLL1* and *JAG1* in YS and *DLK1, JAG1/2, NOV*, and *DLL4* in BM) (Fig. 4D). YS endoderm was predicted to support HSC pool expansion (37) through *WNT5A* signaling to *FZD3*. *WNT5A* was also expressed by a wide range of BM stromal cell types, but BM HSPCs were predicted to respond via *FZD6* rather than *FZD3*. All YS stromal fractions contributed to extracellular matrix, which provides a substrate for adhesion but also modifies HSPC function, with *FN1* (from all fractions) potentially expanding the HSPC pool and *VTN* (from endoderm) contributing to long-term HSC-like quiescence (Fig. 4, D and F) (38, 39). Although BM HSPCs were also predicted to adhere to extracellular matrix proteins, the integrins and matrix constituents differed. YS endoderm was predicted to form unique interactions with HSPCs via *EPO*, which may influence the fate of differentiating progenitors (40), and *THPO*, which supports HSC quiescence and adhesion in BM (41). No BM stromal source of *EPO* or *THPO* was detectable in our data however (10, 35). Thus, these anatomically different hematopoietic tissues use similar pathways to support HSPCs, albeit with tissue-specific components.

YS HSPC receptor to stromal ligand interactions diminished between CS17-CS23 (4-8 PCW), including loss of cytokine and growth factor support and loss of *TFGB1, WNT*, and *NOTCH2* signals (Fig. 4E; fig. S7E; and data S29). In many interactions, there was reduction in HSPC receptor expression as well as stromal ligand expression (Fig. 4E; and fig. S7, D and E; and data S29), yet ligands were still expressed in age-matched liver and AGM stromal cells (fig. S7F and data S29). Adhesive interactions in YS were also predicted to be significantly modulated (fig. S7, F and G, and data S29). Although aged-matched liver provided opportunities for adhesion with stromal cells, the AGM did not (fig. S7F and data S29). YS interactions gained between CS17 and CS23 included endoderm-derived *IL13* signaling to the *TMEM219*-encoded receptor implicated in the induction of apoptosis (Fig. 4D). Although limited conclusions can be made from studying cells that passed quality control for cell viability, we did observe upregulation in proapoptotic gene scores in late-stage YS HSPCs, both early and definitive (fig. S7H).

Despite marked change in the stromal environment of later stage YS, the proportion of HSPC to cycling HSPC remained stable (fig. S5F). Differential lineage priming analysis revealed that very few HSPCs remained in CS22-23 (8 PCW) YS and most cells were terminally differentiated (fig. S6C). Thus, it is likely that an early burst of early HSPC production arises from transient YS HE, a later influx of definitive HSPCs derives from AGM, and a loss of stromal support between 6-8 PCW, results in apoptosis and depletion of remaining HSPCs by terminal differentiation.

## An accelerated route to macrophage production in YS and iPSC culture

**Fig. 5:**
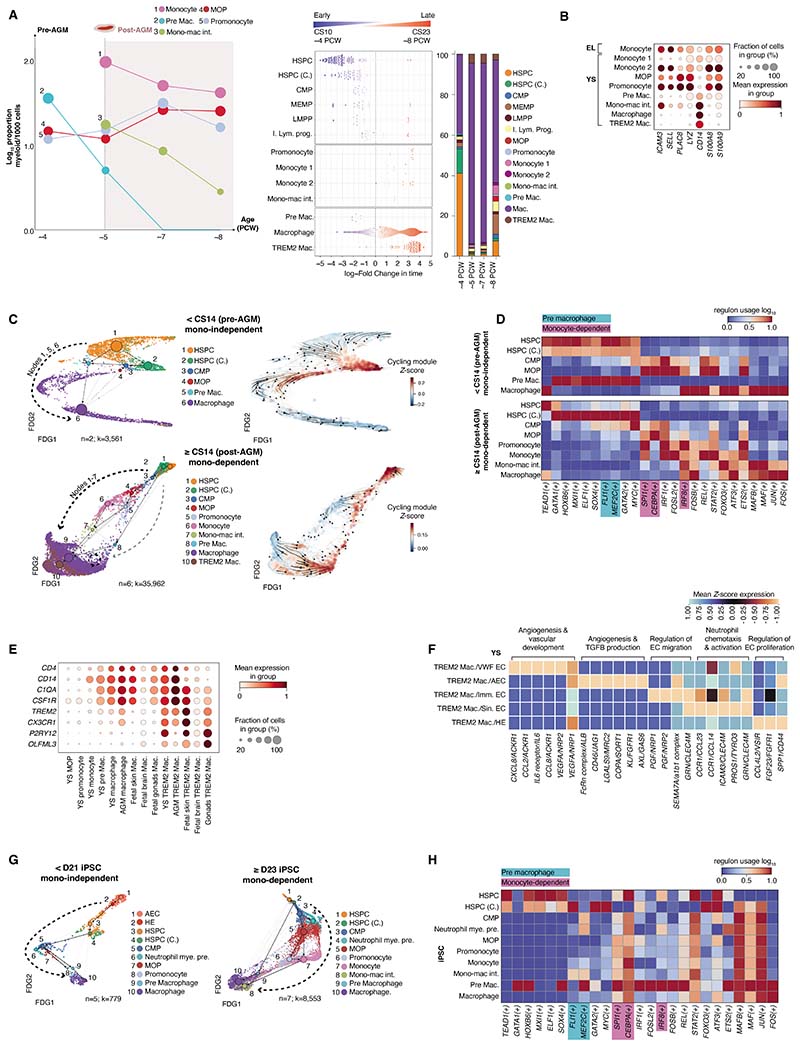
Accelerated macrophage production in YS and iPSC culture. **(A)** Left: Line graph of monocyte and macrophage proportions in YS scRNA-seq across time. Dashed line indicates pre- and post-AGM stages. Middle: Milo beeswarm plot showing differential abundance of YS scRNA-seq myeloid neighbourhoods across time. Color shows degree of enrichment (blue: early, red: later) (data S4 and S24). Right: Bar chart of YS scRNA-seq myeloid cell state proportions across time. Mono–mac int. monocyte macrophage intermediate. **(B)** Dot plot showing the mean expression (color) and proportion of cells expressing monocyte marker genes (dot size) in EL monocytes and YS myeloid cell states. Genes include YS vs EL monocyte DEGs and established monocyte markers (data S17). **(C)** Left: FDG of macrophage trajectory in YS scRNA-seq, colored by cell state, overlaid with PAGA showing monocyte-independent <CS14 (pre-AGM; n=2; k=3,561; top) and monocyte-dependent trajectories >CS14 (post-AGM; n=6; k=35,962; bottom) (data S5). Right: FDG overlaid with scVelocity directionality, colored by cell cycle gene enrichment (GO:000704 module). **(D)** Heatmap of regulons associated with trajectories in **C**. TFs discussed in text highlighted (turquoise: pre-macrophage; purple: monocyte-dependent). **(E)** Dot plot showing the mean expression (color) and proportion of cells expressing macrophage and microglia marker genes (dot size) in myeloid cell states in YS, AGM (12), skin (49), gonad (50), and brain (56) fetal scRNA-seq datasets (data S13 and S31). **(F)** Heatmap of significant (*P*<0.05) CellphoneDB-predicted interactions between YS scRNA-seq TREM2^+^ macrophages and ECs (data S28). Color represents *z*-scored expression of gene pairs, brackets indicate top curated interactions for cell-state pairs. **(G)** FDG of macrophage trajectory in iPSC scRNA-seq (20), colored by cell state, overlaid with PAGA showing monocyte-independent <D21 (n=5; k=779; left) and monocyte-dependent >D21 (n=7; k=8,553; right) transitions (data S7 and S5). **(H)** Heatmap of regulons associated with iPSC macrophage trajectories shown in **G**. TFs discussed in text are highlighted as in **D**.

Although YS hematopoietic progenitors are restricted to a short time window in early gestation, mouse models suggest that they contribute to long-lived macrophage populations in some tissues (42). By scRNA-seq, transcriptionally similar macrophage populations can be identified in YS and fetal brain prior to the emergence of definitive HSPCs (9). In our previous work, k=6682 YS macrophages resolved into two subgroups (10). By contrast, our integrated dataset of k=45,118 YS macrophages in the current study revealed a greater heterogeneity including pre-macrophages, *C1QA/B/C* and *MRC1-*expressing macrophages, and a rare *TREM2*^+^ macrophage subset (fig. S8A). Promonocytes expressing *HMGB2, LYZ*, and *LSP1* and monocytes expressing *S100A8, S100A9* and *MNDA* were also detected (fig. S8A). Monocytes were observed only after liver development and AGM-derived hematopoiesis at CS14 (˜5 PCW), but pre-macrophages and macrophages formed as early as CS10 (˜4 PCW) (Fig. 5A and fig. S8B). Although the potential of early YS HSPCs to differentiate into monocytes has been demonstrated in vitro (9), there were too few promonocytes and monocyte progenitors in our data prior to CS14 to reliably confirm this potential. We identified two populations of YS monocytes, which diverged in expression of adhesion molecules. YS Monocyte 2 expressed adhesion molecules *ICAM3, SELL*, and *PLAC8* (Fig. 5B), which were also expressed on fetal liver but not YS HSPCs (fig. S5C). YS Monocyte 2 had a high probability of class prediction against FL monocytes (fig. S8C). Thus, Monocyte 2 is likely a recirculating FL monocyte, although sequential waves of monocytopoiesis occurring within the YS cannot be excluded. YS CITE-seq data was used to identify discriminatory markers (CD15 and CD43 for Monocyte 1; CD9 and CD35 for Monocyte 2) and provide protein-level validation for differential expression of *SELL* (CD62L) and CD14 (fig. S8D and data S20).

The YS pre-macrophage uniquely expressed high levels of *PTGS2, MSL1*, and *SPIA1*, as well as expressing progenitor genes (*SPINK2, CD34*, and *SMIM24*), macrophage genes (*C1QA* and *MRC1*), and *CD52*, which is typically associated with monocytes (fig. S8A). This YS pre-macrophage rapidly declined by 5 PCW (Fig. 5A) and had no equivalent in embryonic liver (fig. S8C), KNN graph-based FDG and partition-based graph abstraction (PAGA) suggested a direct monocyte-independent trajectory to YS macrophages prior to CS14 (Fig. 5C and data S5). In this pre-AGM trajectory, a transition from HSPC to pre-macrophages, then macrophages (nodes 1, 5, and 6 in Fig. 5C upper panel) fit with our predictions that pre-AGM HSPCs exhibit myeloid bias (Fig. 3G). After CS14, there was a clear differentiation trajectory from cycling HSPC to monocytes and monocyte–macrophages (nodes 1-7 in Fig. 5C lower panel). After CS14, 15.33% of this macrophage pool was proliferating and CellRank RNA state transition analysis was in keeping with active self-renewal (fig. S8E and data S5). Using PySCENIC, YS pre-macrophages were predicted to employ a group of transcription factors (TFs), including *FLI1* and *MEF2C*, that have been reported in the differentiation of multiple lineages (43, 44). By contrast, the monocyte-dependent route (CMPs, monocyte progenitor (MOP), promonocytes and monocytes) relied on recognized myeloid transcription factors such as *SPI1, CEBPA*, and *IRF8* (Fig. 5D and data S30). *TREM2*^+^ macrophages expressed microglia-associated transcripts *CX3CR1, OLFML3*, and *TREM2* and were observed in YS only after CS14 (Fig. 5A, C and E; fig. S8A; and data S13). By PAGA and CellRank state transition analysis, *TREM2*^+^ macrophages were closely aligned to the self-renewing macrophage population (Fig. 5C and fig. S8E). YS *TREM2*^+^ macrophages were located adjacent to the mesothelium, in a region enriched by EC (fig. S8F). CellPhoneDB predicted interactions between *TREM2*^+^ macrophages and *VWF*^+^ EC, via *CXCL8* and *NRP1*, both of which are involved in angiogenic pathways (45, 46) (Fig. 5F and data S28). *TREM2*^+^ macrophages also expressed the purinergic receptor *P2RY12*, which supports trafficking towards ATP/ADP-expressing ECs, as reported in the mouse CNS (47), (48) (Fig. 5E and data S31). To establish whether *TREM2*^+^ macrophages are present in other fetal tissues, we assembled an integrated 12-organ developmental atlas (fig. S8G). We resolved six macrophage fractions based on harmonized cross-tissue definitions from our recent prenatal immune analysis (by label transfer): pre-macrophages and TREM2^+^ macrophages (as in our cluster-driven annotations), as well as LYVE1^hi^, Kupffer-like, iron-recycling, and proliferating macrophages (49) (fig. S8, C and G to J, and data S5 to S7 and S17). TREM2 is implicated in lipid sensing by anti-inflammatory tissue macrophages in the adult human and mouse (23–25), but we observed the highest expression of *TREM2* in macrophages bearing a “microglia-like” signature in developing tissues including YS, skin (as previously reported (49)), gonads (as previously reported (50)), brain, and AGM, but not BM, liver, kidney, thymus, mesenteric lymph nodes (MLNs), or gut (fig. S8, I and J, and data S31).

Next, we asked whether transcriptional features of pre-AGM macrophages could be used to evaluate YS macrophage contribution to developing tissues. In our 12-organ macrophage dataset, pre-AGM macrophages were compared against post-AGM macrophages in an integrated variational-autoencoder (VAE) latent space using a Bayesian differential expression approach. The most predictive pre-AGM macrophage features comprised nine genes, including five genes in common with a “TLF^+^ signature” identified from cross-tissue analysis of mouse macrophages (*LYVE1, TIMD4, FOLR2, MRC1*, and *NINJ1*) (51) (fig. S9A and data S17). By KDE, macrophages significantly enriched in our pre-AGM module colocalized with LYVE1^hi^ macrophages from gonads, liver, skin, and AGM, and with all macrophage fractions from the YS (fig. S9, B to D; fig. S8H; and data S7 and S32). The proportion of pre-AGM module-enriched macrophages trended downwards over time, even in the brain (fig. S9E). By transcriptome alone, it was not possible to separate dilution by influx of non-YS macrophages from transcriptional adaptation to the tissue environment. With this caveat, we assembled a 20-organ, cross-tissue integrated landscape of adult tissue macrophages using publicly available single-cell data from the Human Cell Atlas and Tabula sapiens (fig. S9, F to H; and data S6, S7, S14, and S17). Fat, vasculature, muscle, brain, and bladder had the highest proportion of macrophages enriched in the pre-AGM signature (fig. S9H and data S7).

We integrated our YS gene expression data with scRNA-seq data from iPSC-derived macrophage differentiation (n=19; k=50,512) (20) after refining the annotations of iPSC-derived cell-states (fig. S10, A to C; and data S5 and 13). Non-adherent, *CD14*-expressing cells appearing after week 2 of differentiation expressed *C1QA, C1QB*, and *APOC1* in keeping with a macrophage identity, while C*D14, CD52, FCN1*, and *S100A8/9*-expressing monocytes only emerged after week 3 (fig. S10, D and E). Prior to monocyte emergence, a monocyte-independent macrophage differentiation trajectory was observed, consistent with previous observations (20) (Fig. 5G and fig. S10C). TF regulatory profiles of iPSC-derived macrophage differentiation were consistent with the both pre-macrophage and monocyte-dependent TF profiles inferred from our YS data, including usage of *MEF2C, SPI1, CEBPA*, and *IRF8* in iPSC-derived pre-macrophages (Fig. 5H and data 30). However, neither iPSC culture system could recapitulate the heterogeneity of macrophages seen in native tissues (fig. S10E), suggesting that interactions with stromal cells, such as ECs, may be required to acquire specific molecular profiles.

## Discussion

Using single-cell multiomic and imaging technologies, we delineate the dynamic composition and functions of human YS in vivo from 3 PCW, when the three embryonic germ layers form, to 8 PCW when the majority of organ structures are already established (21). Although the scarcity and small sample size necessitated a primarily computational approach, we deliver a comprehensive resource. LR and VAE models provided by our data will facilitate future use of our YS atlas to map scRNA-seq datasets (52, 53), empowering future mechanistic perturbation and lineage-tracing experiments in iPSCs and model systems.

We detail how YS endoderm shares metabolic, biosynthetic, and erythropoiesis-stimulating functions with the liver. In part, this shared functionality may relate to their common role in creating a hematopoietic niche (54). We identify differences in the handover from YS to liver hematopoiesis between species. In mice, erythroid progenitors in the YS mature prior to the onset of circulation, but erythromyeloid progenitors can exit the YS and mature in the fetal liver, giving rise to long-lived populations such as fetal liver monocyte-derived macrophages. We show that in human YS, active differentiation of erythroid and macrophage cells occurs for several weeks prior to liver handover, and, at least in terms of erythropoiesis, there is a rapid transition from YS erythroid production to embryonic liver erythroid production shortly after AGM-derived HSPCs emerge. In a landmark study on human Hb switching, directly-labeled 6 PCW liver and YS erythrocytes contained embryonic Hb subunits (ε and ζ), but colonies derived from liver and YS progenitors at this time produced fetal Hb subunits (α and γ) (8). This is in keeping with YS-derived erythrocytes recirculating throughout the embryo and membranes while a post-AGM progenitor is preparing for liver erythropoiesis. Direct evidence that human liver erythropoiesis is supplied predominantly from AGM-derived HSPCs rather than a YS-derived “EMP-like” progenitor is still lacking. Future studies are also needed to examine the handover of macrophage production from early to definitive sources in humans, which may question the primacy of mouse models of early myelopoiesis. A more expansive species reference, including rabbits with their greater early gestational similarity to humans, will facilitate selection of appropriate models for genetic manipulation and functional validation (55).

The developmental window investigated here encompasses hematopoiesis from HSPCs arising both within the YS and within the embryo proper. We reconstructed YS HSPC emergence from a temporally restricted HE, featuring similar transition states and molecular regulation to AGM HSPCs. By gastrulation (CS7; 2-3 PCW), YS HSPCs already differentiate into erythroid, MK, and myeloid lineages. Building on a recent compilation of gene scorecards that characterize early and definitive HSPCs (12), we were able to parse the two fractions and document transition to definitive HSPC-dominance after CS14 (˜5 PCW). This separation also allowed us to identify an early HSPC bias towards myeloid, erythroid, and MK lineages and a definitive HSPCs bias towards MK and lymphoid lineages. Both early and definitive YS HSPCs became more quiescent and upregulated apoptosis-related genes between CS17 and CS23 (˜6-8 PCW). Stromal cell ligands predicted to support HSPCs were markedly disrupted during this time, suggesting that the barriers to YS HSPC survival may be extrinsic.

Early HSPCs uniquely employ an accelerated route to macrophage production independent of monocytes. Both “accelerated” and monocyte-dependent macrophages were recapitulated during in vitro differentiation of iPSCs, but diverse macrophage subtypes such as *TREM2*^+^ macrophages were not. *TREM2*^+^ macrophages, which are transcriptionally aligned with brain microglia, fetal skin, testes, and AGM *TREM2*^+^ macrophages, were predicted to interact with endothelial cells, potentially supporting angiogenesis as described in mouse brain (56).

There is a growing appreciation of the potentially life-long consequences of early developmental processes. Our study illuminates a previously obscure phase of human development, where vital organismal functions are delivered by a transient extraembryonic organ employing non-canonical cellular differentiation pathways that can be leveraged for tissue engineering and cellular therapy.

## Materials and Methods

### Ethics and sample acquisition

Tissues were obtained from the MRC–Wellcome Trust-funded Human Developmental Biology Resource (HDBR; http://www.hdbr.org) with appropriate written consent and approval from the Newcastle and North Tyneside NHS Health Authority Joint Ethics Committee (18/NE/0290). HDBR is regulated by the UK Human Tissue Authority (HTA; www.hta.gov.uk) and operates in accordance with the relevant HTA Codes of Practice. Tissues used for light-sheet fluorescence microscopy were obtained through INSERM’s HuDeCA Biobank and made available in accordance with the French bylaw (Good practice concerning the conservation, transformation and transportation of human tissue to be used therapeutically, published on December 29, 1998). Permission to use human tissues was obtained from the French agency for biomedical research (Agence de la Biomédecine, Saint-Denis La Plaine, France).

Embryos were staged using the Carnegie staging method (57). A piece of skin or chorionic villi tissue was collected from each sample to perform quantitative PCR karyotyping of sex chromosomes and autosomal chromosomes 13, 15, 16, 18, 21, and 22 for the most commonly seen chromosomal abnormalities. No abnormalities were detected.

### Processing samples for imaging and single-cell sequencing

Tissues were transported in phosphate-buffered saline (PBS) on ice, were dissected within 24 hours, and were processed immediately (<1 hour after dissection). For formalin-fixation and paraffin-embedding, samples were immediately placed in 10% (w/v) formalin. Processing and embedding were performed by NovoPath, Newcastle upon Tyne NHS Trust. For RNAscope, samples were snap-frozen in an isopentane bath in liquid nitrogen prior to embedding in optimal cutting temperature (OCT) compound. Single-cell suspensions were generated by dicing tissue into segments <1 mm^3^, followed by enzymatic digestion for 30 min at 37°C with intermittent shaking. Digestion media was 1.6 mg/ml collagenase type IV (Worthington) in RPMI (Sigma-Aldrich) supplemented with 10% (v/v) heat-inactivated fetal bovine serum (FBS; Gibco), 100 U/ml of penicillin (Sigma-Aldrich), 0.1 mg/ml of streptomycin (Sigma-Aldrich), and 2 mM L-glutamine (Sigma-Aldrich). Digested tissue was passed through a 100-μm filter, and cells were collected by centrifugation (500*g* for 5 min at 4°C). Cells were treated with 1X RBC lysis buffer (eBioscience) for 5 min at room temperature and washed once with Flow Buffer (PBS containing 5% (v/v) FBS and 2 mM EDTA) before counting. Processing for scRNA-seq was continued promptly on fresh cells, for other uses (including CITE-seq) cells were collected by centrifugation (500*g* for 5 min at 4°C) and resuspended in 10% (v/v) DMSO in FBS for freezing. For light-sheet fluorescence microscopy (LSFM), tissues were fixed in 4% PFA and dissected. Gestational age was then estimated as previously described (58).

### Processing of single-cell suspensions for scRNA-seq

Immediately following isolation and counting, cells were collected by centrifugation (500g for 5 min at 4°C) and resuspended in a residual buffer. Three microliters of CD45 BUV395 (clone: HI30, BD Biosciences) was added to the resuspended cells and incubated on ice in the dark for 30 min, washed with Flow Buffer and resuspended at ˜1×10^7^ cells/ml. Immediately prior to sorting, cells were passed through a 35-µm filter (Falcon) and DAPI (Sigma-Aldrich) was added at a final concentration of 3 μM. Flow sorting was performed on a BD FACSAria Fusion instrument using DIVA v.8, and data were analyzed using FlowJo (v.10.4.1, BD Biosciences). Cells were gated to exclude dead cells and doublets, and then isolated for scRNA-seq analysis (droplet-based 10x Genomics, or plate-based Smart-seq2) using a 100-µm nozzle. For droplet-based scRNA-seq, CD45^+^ and CD45^−^ cells were sorted into separate chilled fluorescence-activated cell sorting (FACS) tubes coated with FBS and prefilled with 500 µl of sterile PBS. For plate-based scRNA-seq, CD45^−^AF^+^SSC^++^ single cells were index-sorted into 96-well LoBind plates (Eppendorf) containing 10 µl of lysis buffer (TCL (Qiagen) + 1% (v/v) β-mercaptoethanol) per well.

### Library preparation and sequencing of scRNA-seq and CITE-seq samples

For the droplet-based scRNA-seq experiments, cell suspensions isolated by FACS were counted and loaded onto the 10X Genomics Chromium Controller to achieve a maximum yield of 10,000 cells per reaction. 5‏ V1 kits were used and sequencing libraries were generated according to the manufacturer’s protocols. Libraries were sequenced using either an Illumina HiSeq 4000 or NovaSeq 6000 to generate at least 50,000 raw reads per cell.

For the plate-based scRNA-seq experiments, the frozen cell lysates were thawed on ice for 1 min. Purified cDNA was generated and amplified using a modified Smart-seq 2 protocol described in Villani et al (59). Sequencing libraries were then generated using Illumina Nextera XT kits with v2 index sets A, B, C and D. 384 cells were pooled and were sequenced using a HiSeq 4000 to generate at least 1×10^6^ raw reads per cell.

For the CITE-seq experiments, frozen cells were thawed, counted, and pooled. Fc blocking reagent (Biolegend) was added to the cell pools and left to incubate at room temperature for 10 min. Five hundred nanoliters of CD34 APC/Cy-7 (clone: 581, Biolegend) was then added to the Fc-blocked cells and left to incubate in the dark and on ice for 10 min. During this incubation, the CITE-seq antibody cocktail (Biolegend) (see data S33) was centrifuged at 14,000*g* for 1 min. Flow buffer was then added to reconstitute before incubating for 5 min at room temperature. The resuspended antibody cocktail was then centrifuged at 14,000*g* for a further 10 min before adding to the cells. The cells and CITE-seq antibody cocktail were then left to incubate for 30 min in the dark and on ice. After this time, the cells were washed twice with Flow buffer and resuspended in a final concentration of 50 μg/ml of 7 AAD (Thermo Fisher Scientific) in Flow buffer.

Live, single cells or live, single CD34^−^ cells and live, single CD34^+^ cells (for the CITE-seq experiments) were then isolated by FACS into 500 µl of PBS in FACS tubes coated with FBS. Cells were then counted and submitted to the CRUK CI Genomics Core Facility for subsequent processing using 10x Genomics protocols and sequencing. Single-cell gene expression and cell surface protein libraries were generated using Single cell 3‏ v3 kits according to the manufacturer’s protocol. Libraries were sequenced using a NovaSeq 6000 to achieve a minimum of 20,000 reads per cell for gene expression and 5000 reads per cell for cell-surface protein.

### Alignment, quality control, filtering, and preprocessing of scRNA-seq and CITE-seq data

scRNA-seq expression data (including droplet-based and plate-based) were mapped with CellRanger (version 3.0.2) to a human reference genome (see data S1) and low-quality cells expressing <2000 reads, <200 genes, and >20% mitochondrial reads were filtered out of the data. Data on genes expressed in fewer than three cells was removed.

For droplet-based scRNA-seq data, the following additional QC steps were performed. Scrublet (60) v0.2.3 was applied to each sequencing lane for doublet detection, and clusters with >(Median+(1.48*MAD)) (MAD: Median absolute deviation) of the median cluster doublet detection score were removed (data S3). Ambient RNA was removed with Cellbender (v0.2.0) with fdr=0.01 and epochs=150 (61). To determine likelihood of maternal contamination, data were pooled by donor and submitted to Souporcell (v2.4.0) at genotype clusters k=1 and k=2 models to represent likelihood of no maternal contamination and possible maternal contamination, respectively. The optimal model was identified via BIC (Bayesian Information Criterion), where we observed a smaller BIC index at k=2 in one donor (F37, Female, 5 post conception weeks (PCW)). Cells from the F37 alternate genotype were identified as potential maternal contaminants, composed mainly of monocytes (n=149), and monocyte–macrophage intermediates (mono–mac int.) (n=25), and excluded from downstream analysis.

For CITE-seq data, FASTQ alignment was performed for multiplexed RNA lanes with CellRanger (v4.0.0) and GRCh38-2020-A reference genome, and for multiplexed protein lanes with CITE-seq-Count (v1.4.3). Lanes with cells pooled from multiple donors were deconvoluted using Souporcell singularity image at https://github.com/wheaton5/souporcell. Low quality cells expressing <200 genes and >20% mitochondrial reads were removed and doublets were removed by applying Scrublet v0.2.3 to each sequencing lane and then removing clusters with >(Median+(1.48*MAD)) of the median cluster doublet detection score. CITE-seq protein data underwent QC and preprocessing as previously described (35), (i.e., cells were first filtered to intersect barcodes with counterpart CITE-seq RNA data, then unmapped antibodies were filtered out and then protein cells were filtered for low quality by cells with <30 proteins and expressing >5000 reads) (data S4, S19, and S22).

scRNA-seq count matrix transformation, normalization, and preprocessing were performed using Scanpy (62) (v1.9.0) in python (v3.8.6). We normalized raw gene counts using the *sc.pp.normalize_total* function (*target_sum* = *10e4*) from and performed *ln(x)+1* transformation. Reported expression values were normalized, log-transformed, and scaled to variance of mean using the *sc.pp.scale* function independently for each analysis.

For CITE-seq data count matrix transformation, we first performed denoised and scaled by background (DSB)-normalization and applied a Gaussian Mixture Model (GMM) for background non-specific binding signal regression per sample as previously described (63). For the first step, a modified DSB-normalization approach used in our previous study (35) was constructed. For each CITE-seq lane, low quality/empty droplets were identified as droplets under the largest UMI peak which had a value <1.96*standard deviations (std) of the mean UMI counts value (mu_UMI) per sample. Peak detection was conducted using the *scipy.signal.find_peaks* function. The number of peak detection bins were dynamically estimated as (3.322*log(X)), where X was the total number of droplets. The model iterated through a series of 20 prominence intervals (0-20) with widths (0-10) where peaks detected < (mu_UMI-(1.96*std)) were retained as empty droplet peaks. In cases where no empty droplet peaks were detected, the empty droplet threshold was taken to be <(mu_UMI-(1.96*std)). The estimated empty droplets matrix was then taken into downstream DSB normalization in the same way as our previous study (35). For the second step of CITE-seq matrix transformation, we trained a GMM to model the variance of protein expression levels in each cell. We used the sckitlearn (v.1.1.3) *sklearn.mixture.GaussianMixture* module to fit 20 models with an increasing number of cell clusters *k* (between *k*=2 and *k*=21) to represent expression patterns of each protein by cell. The optimal model was identified using BIC (BIC_i_ = 2L_i_ + kilog*n*) and AIC (Akaike information criterion) (AIC_i_ = 2L_i_ + 2k_i_) metrics where *k* is the number of GMM cell protein expression clusters, n is the number of cells in the sample and L is the model log likelihood. The models with the best performing BIC and AIC scores were selected.

The mean expression values of GMM clusters with lowest expression values from each GMM model were interpreted as mean background expression per protein. Background-signal regression was then carried out using a Gaussian linear model (GLM) per protein, constructed using the *GLM* function from *statsmodels (v0.13.5)* on standardized, per cell background scores (BG_score). Per-cell background scores were defined by taking the euler number (*e*) to the power of each protein background mean (*mu_bg*), divided by *e* to the power of the protein expression in each cell (*x*) then scaled to a distribution between 0-1 by subtracting the minimum score and subsequently dividing by the maximum score. Background scores inversely correlate with the magnitude of background expression per cell. (BG_score = (score - min(*e*^mu_bg^/*e*^x^))/max(*e*^mu_bg^/*e*^x^)). The per-cell background signal regressed counts were used for subsequent analyses, interpretation and visualization. Cells comprising the empty droplet matrix were removed and were not considered for downstream analyses.

### Integration and batch correction of scRNA-seq and CITE-seq datasets

For integration of newly generated yolk sac (YS) scRNA-seq data with external datasets, CellRanger count was first reapplied for the alignment of CS10/CS11 and CS14 embryonic YS scRNA-seq data previously acquired (12, 64) (data S1). The following steps were then followed for the total integrated YS droplet-based scRNA-seq dataset. Highly variable gene (HVG) selection was performed using the *sc.pp.highly_variable_genes* function (min_mean=0.001, max_mean=10) for embedding by dispersion. Dimensionality reduction and batch correction for the was carried out using the scVI module within *scvi-tools* (v0.19.0) (53) as used in scvi-tools (52) (HVG = 7500, dropout_rate=0.2, n_layer=2) with biological replicate taken as the technical covariate. To ensure model performance was optimal for each independent analysis, scVI was benchmarked against the python implementation of Harmony (65) (*Harmonypy* v0.0.5) at various theta values between 1 and 20. kBET (66) and Silhouette scores (*sklearn.metric.sil_score*) were computed for each iteration between donor covariates and compared to the scVI integration. For integration of adult scRNA-seq data, publicly available single-cell and single-nuclei RNA-seq data of 20 healthy adult tissues (data S6 and S7) were integrated using scVI (HVG=1500, layers=1). Batch correction was conducted on donors, single cell or single nuclei, data source, number of genes, total counts, percentage of mitochondrial genes and ribosomal genes. Please see data S6 for information regarding external single-cell RNA sequencing (scRNA-seq) datasets that have been incorporated and integrated in this study.

For multimodal integration of CITE-seq datasets, we compared the integration of both RNA and protein modalities using the *totalVI* module in *scvi-tools* (v0.19.0) against batch integration utilizing only the RNA modality the scVI module in scvi-tools. We performed *sc.pp.highly_variable_genes* function on RNA modality (HVG=4000) accounting for FACs sampling and donor technical covariates. We then generated a multi-modal totalVI VAE latent representation following totalVI pipeline (53). To benchmark performance between multi-modal and single modality scRNA-seq data integration, global silhouette distances between leiden clusters (res=3) derived from the multi-modal totalVI VAE latent representation were compared against clusters derived from the scVI derived VAE representation as described above (HVG = 4000, dropout_rate=0.2, n_layer=2) (fig. S11A).Intersecting cells captured between protein and RNA modalities (by barcode) were considered in the totalVI integration (fig. S11, B to D; and data S4 and S22).

All scVI VAE and ldVAE models trained on the YS and integrated atlases are available on our data portal (see data availability) and will facilitate transfer learning for future reference mapping of scRNA-seq data with single cell architectural surgery (scArches) (18)

### Clustering and annotation of scRNA-seq and CITE-seq data

Clustering of scRNA-seq and CITE-seq datasets was performed using the Leiden algorithm (67) (*sc.tl.leiden*) with a resolution parameter of *res*=1.5 (CITE-seq res=3) on a k-nearest neighborhood graph (k=30 for scRNA-seq and k=15 for CITE-seq) unless specified otherwise. To measure the effect of decreasing graph complexity on specific populations (YS scRNA-seq endoderm and IPSC-derived macrophages) and global population specificity and homogeneity in each independent analysis, the neighborhood graph complexity parameter (k) was benchmarked at value intervals between 5 and 50. Benchmarked metrics for population specificity included the adjusted mutual information score (MI), and adjusted Rand index (RAND). Metrics for population homogeneity included the silhouette index (SI) and within-cluster sum of squared errors (WSS) (fig. S12, A and B; and data S3 and S7). In cases where datasets are compared probabilistically, or where new classifications have been made, an implementation of low-dimensional ElasticNet regression (EN) (described in the “Cell state predictions using probabilistic low-dimensional ElasticNet regression” section of the manuscript methods) was used to first classify individual cells where a model-specific decision threshold of 0.9 was used for classification tasks. Cells classified inherited labels from the model trained on YS scRNA-seq data. Clusters were then assigned classes if the majority projected label had a label count distribution of >(mean + (1*std)) of label counts per cluster. Resultant cell state classifications were further manually checked using differentially expressed genes using the *sc.tl.rank_genes_groups* function in Scanpy which performed a two-sided Wilcoxon rank-sum test for genes expressed in >25% of cells, with a log-transformed fold change cut-off of 0.25. All p-values were adjusted for multiple testing using the Benjamini–Hochberg method. Annotation of YS and liver CITE-seq data was performed by training an EN model using YS and Embryonic Liver (EL) scRNA-seq datasets as references respectively. These labels were then distributed by majority voting onto Leiden clusters derived from CITE-seq data (data S9 and S10). The resultant cluster annotations were validated using the same markers identified in matched RNA data and underwent additional manual annotation where required. For differential expression testing of surface proteins in multi-modal CITE-seq data, we conducted a one_vs_all DE test using the *vae.differential_expression module* within totalVI (Bayes factor>3, Median LFC>0.25). Marker proteins and corresponding populations were subsequently subject to hierarchical grouping using the *sc.tl.dendrogram* functionality within Scanpy (fig. S11, B to D; and data S4, S20, and S22). Bayesian differential expression testing between cell states in the integrated 12 fetal organ atlas was carried out on using a *scVI* integrated latent VAE representation with a one_vs_all DE test using the *vae.differential_expression module* within scVI (v0.19.0) (Bayes factor>3, Median LFC >4). Variation of effect-sizes on state-specific normalized counts between latent variables in the integrated latent VAE representation were first modeled. The posterior likelihood of differential expression was attained by repeated one_vs_all sampling of the variational distribution. Significant features were defined with a likelihood of differential expression (Bayes Factor) >3 and Median LFC >4. Bayesian differential expression testing between myeloid cell states in the integrated 20 organ adult scRNA-seq atlas was carried out as described above (fig. S12 C; and data S6, S7, and S17).

### Dimensionality reduction and marker expression visualization

For visualization, the uniform manifold approximation (UMAP) algorithm was run using the *sc.tl.umap* function in Scanpy. Dot-plots and violin plots were produced in Scanpy and all gene expression values displayed were normalized, log-transformed, and scaled as described in the preprocessing section unless otherwise stated. Dot plots that display data from multiple datasets employed independent log-normalization, variance scaling, and min–max standardization to a distribution of 0-1 per dataset unless otherwise stated. Force-directed graphs (FDGs) computed with the *sc.tl.draw_graphs* function in Scanpy using the Force Atlas2 parameter were used to infer trajectories. Partition-based graph abstraction (PAGA) were computed on the k-nearest neighbor graphs and overlaid onto FDGs where nodes represented the centroid of each cell state cluster and the thickness of edges represented the similarity between cell states (data S5).

Proportion line graphs for specific populations (e.g., erythroid cells) enriched in specific genes (e.g. *HBZ*) using the *sc.tl.enrich* function in Scanpy were produced using Matplotlib (v3.6.2). To ensure temporal changes in population size and background expression were accounted for, we segregated our population of interest by age and computed changes in relative population proportion enriched in each gene, only considering cells expressing >0 log-normalized counts for each gene. Enriched cells were defined with >0 score of each scored gene subtracted with the mean expression of a randomly sampled set of 200 selected reference genes at 50 bins using the aforementioned enrichment function in *Scanpy*. Proportions of enriched cells in each cell type compartment were then plotted as a discrete time-series across gestational age to visualize differential enrichment of cells expressing the genes of interest. Data point sizes represented enriched cell counts. To aid interpretation, an ordinal scale of representative cell counts was included as a legend in the plots.

Proportions of specific populations (e.g., macrophages) enriched in specific gene modules (e.g., Pre-AGM module) were visualized using violin graphs produced using Matplotlib and *Seaborn* (v0.12.1) python libraries. To ensure background expression profiles were accounted for, we segregated our population of interest and computed changes in relative population proportion enriched in each gene module. Significant module enrichment was defined as described above. Enriched cells from each cell type compartment were then graphed across organs. Enrichment scores were standardized to the median by subtraction of the median and subsequent division by MAD.

### Differential abundance testing and FACS correction

We tested for differential cell-state abundance across gestation using the *Milo* framework (68), correcting for CD45 positive and negative FACS isolation strategies using a previously published technique (49). Where FACs correction was applied, we calculated a FACS isolation correction factor for each sample *s* sorted with gate *i* as (*fs* = log(*pi*S/Si)) where *pi* is the true proportion of cells from gate *i* and S represents the total number of cells from both gates. A KNN graph was then constructed from the remaining cells using the *milopy.core.make_nhoods* function (prop=0.05). Neighborhood labels were determined by majority voting of cell labels by frequency in each neighborhood (>50%). The YS scRNA-seq data was then split into five age bins (3 PCW, 4 PCW, 5 PCW, 7 PCW, and 8 PCW) and cell counts were modeled as a negative binomial generalized linear model (NB-GLM) with Benjamini–Hochberg weighted correction as previously described (49). Significantly differentially abundant neighborhoods were detected by SpatialFDR–(<0.1, logFC <0) for early enriched neighborhoods and (<0.1, logFC >0) for late neighborhoods (data S24).

Beeswarm plots were generated using the *ggplot2* library (v3.4.2). Each node represents an independent neighborhood of cells derived from the KNN graph. The *x*-axis position of each node represents the fold-change (positive/negative) associated with the distribution of age groups present in each neighborhood where larger proportions of older groups in a given neighborhood encourages a positive fold change and vice versa. Colored nodes represent neighborhoods with significant enrichment (*P*<0.05 spatial FDR) and the intensity represents the degree of significance.

### Clustered gene-set enrichment analysis

We ranked conserved markers (*P*<0.05) between the endoderm cell state in YS scRNA-seq data against hepatocytes in EL scRNA-seq and endoderm in the mouse gastrulation scRNA-seq data using the *FindConservedMarkers* function in Seurat (v3.1) with Bonferroni corrected FDR adjusted *P*-values. Markers were submitted for gene set enrichment ranking and analysis using the Enrichr tool as implemented in the *GSEApy* (v1.0) package to query the Gene Ontology (GO) Biological Process database (GO_BP_2022) (data S26). Using the enrichrR package (v3.0) in R, enrichment was first computed by Fisher exact test for randomly sampled genes to derive a mean rank and standard deviation to estimate background for each ontological term accessed. A *z*-score for deviation of each term to its background rank was then used to rank output genesets. We derived statistical significance (Fisher exact test <0.05, ranked by *z*-score) for each gene set enrichment and performed Markov clustering (MCL) using the MCL (v1.0) package in R to derive network neighborhoods based on geneset intersect. Gene set clusters were annotated using the AutoAnnotate function in the RCy3 (v2.16) package and clusters were ranked by the mean *z*-score of all gene sets within each cluster and manually curated based on biological significance. We used the Cytoscape software (v3.9.1) to visualize clusters.

### Cell state predictions using probabilistic low-dimensional ElasticNet regression

Label transfer class assignments and median probability of class correspondence between gene expression matrices in single cell datasets were carried out using a logistic regression (LR) framework, as previously described (35), using a similar workflow to the *CellTypist* tool (69).

Raw scRNA-seq datasets being compared were first concatenated, normalized, and log-transformed, as described in preprocessing. HVG selection was performed (min_mean=0.001, max_mean=10) for embeddings by dispersion. HVG expression matrices were used as training inputs for models unless otherwise stated. For models trained in combined low-dimensional representations, linear VAE latent representations were computed using the *LDVAE* module within *scvi-tools* (hidden layers=256, dropout-rate=0.2, reconstruction-loss=negative binomial) with donor, dataset origin, and organ information taken as technical covariates. Where PCs were used as input for training, harmony batch-corrected PCs (k=100pcs) were used, using Harmonypy (v0.0.9) with technical covariates as described above. Harmony runs were iterated through theta=1:20 and resultant embeddings benchmarked using kBET and silhouette scores between technical covariates where a low kBET rejection rate and corresponding high silhouette score denoted the optimal theta parameter.

ElasticNet regression (EN) LR models were built utilizing the “sklearn.linear_model.LogisticRegression” module in the sklearn package (v0.22). The models were trained using either gene expression data or SCVI batch-corrected low-dimensional LDVAE representation of the training data with regularization parameters (L1-ratio and alpha) tuned using the GridSearchCV function in sklearn (v1.1.3). The test grid was designed with five l1_ratio intervals (0, 0.2, 0.4, 0.6, 0.8, 1), five alpha (inverse of regularization strength) intervals (0.2, 0.4, 0.6, 0.8, 1) at five train-test splits and three repeats for cross-validation. The unweighted mean over the weighted mean squared errors (MSEs) of each test fold (the cross-validated MSE) was used to determine the optimal model.

The resultant model was used to predict the probability of correspondence between trained labels and precomputed clusters in the target dataset. To ensure that probabilistic outputs from LR models remained consistent with observed neighborhood graph connectivities, the median LR predicted probability of training label assignment was compared against normalized graph distances between classes computed using the partition-based graph abstraction (PAGA) (tl.paga) module in Scanpy as described in our previous work (10) (fig. S13A). Genes predicted to be significantly discriminatory for each LR model were assessed by significance of impact. Features were ranked in descending manner by impact score (eˆcoefficient per feature for given intercept). Impact significance (*P*<0.05) of each gene was computed by the survival function (sf) across all gene impact scores (fig. S13B). To further verify the specificity of the TREM2 macrophage gene expression profile, the proportion of differentially expressed genes (DEGs) overlapping between the TREM2 macrophage population and other macrophage populations across the 12-organ fetal atlas were computed using a two-sided Wilcoxon rank-sum test as described in the clustering and annotation section (fig. S13, C and D; and data S31).

For dataset comparisons across the 12-organ fetal atlas tasks where predesignated labels already existed in the target dataset, the median probability of training label assignment per predesignated class was computed. The resultant LR probabilistic relationship between labels of the 12-organ atlas were visualized as a heatmap (fig. S14 and S15; and data S8 to S16).

For classification tasks, a model-specific decision threshold of 0.9 was used to determine predicted labels. Clusters were then assigned classes if the majority projected label had a label count distribution of >(mean + (1*std)) of label counts per cluster. Resultant cell state classifications were further manually checked using differentially expressed genes. Further assessment of the predicted cluster labels was carried out by computing the adjusted Rand index and mutual information scores from the modules ‘sklearn.metrics.adjusted_rand_score’ and ‘sklearn.metrics.mutual_info_score’ between the original cluster labels and predicted cluster labels in each dataset. This methodology was applied to classify and annotate several external datasets including the scRNA-seq human gastrulation data (14), the human AGM data (12), the human embryonic liver data and human fetal skin data (49), as well as the human YS and liver CITE-seq data (data S6).

An implementation of the EN workflow described above, in conjunction with the SAMap (Self-Assembling Manifold mapping) workflow (v1.0.7) (70), was used to classify and probabilistically compare cell states across the human YS scRNA-seq data and the mouse gastrulation YS data. A gene–gene sequence homology graph weighted by human and mouse sequence similarity was first constructed using the SAMAP tool. Reciprocal BLAST mapping using the tblastx tool between the entire mouse and human transcriptomes for significant homology (E-value<10^−6^) was supplied. The resultant SAM object returned k=300 species-stitched PC components for the top 3000 paired genes. These PC components were used to train the cross-species EN model as a classification task described above (data S11).

LR models and weights trained on the YS and integrated fetal atlases are available via our interactive web portal in “.sav” format (see data availability) and will facilitate future use of our YS atlas for label transfer and and to rapidly annotate scRNA-seq datasets using the Python package CellTypist (v.0.1.9) (69).

### Differential lineage priming and progenitor cell fate predictions

The CellRank package (v1.5.1) was used to define and rank fate probabilities of terminal state transitions across annotated hematopoietic lineages in the YS and iPSC scRNA-seq datasets. In the YS data, cell clusters broadly annotated to be in the myeloid lineage were first subsetted from the YS data. After refinement, DCs were excluded from this subset. We did not identify any DCs in the <CS14 (pre-agm) myeloid lineage. Macrophage trajectory inference was then constructed across the myeloid subset (fig. S8E). Cells were divided by donors aged <CS14 and >CS14 (post-agm) and trajectory inference recomputed on new embeddings. First-order-kinetics matrices were imputed for each dataset using the *pp.moments* function (n_pcs=20, n_neighbours=30) in the scVelo package (v0.2.4). A Cytotrace pseudotime for state transitions across each dataset was then computed to direct graph-edges towards estimated neighborhood regions of increasing differentiation using the Cytotrace kernel provided within the CellRank package. The resultant KNN and Cytotrace pseudotime were used to compute a probability transition matrix with the *compute _transition_matrix* command in Cytotrace. Neighborhoods of cells representing terminal states of differentiation were identified using true Schur matrix eigen decomposition of the transition matrix *compute_schur* (n_components=20, method=brandts), followed by the *compute_macrostates* (n_states=10) command in Cytotrace. The resultant terminally differentiated cell states were then manually selected if multiple terminal states were identified per lineage. Fate absorption probabilities were then computed across all cells terminating at each prespecified terminal cellstate neighborhood using the *compute_absoprtion_probabilities* command in CellRank. Fate probabilities were then presented as a circular plot using the *pl.circular_projection* with embedding proximity to terminal edges of the graph representing the fate-transition probability of a particular cell towards the prespecified terminally differentiation state. HSPC progenitor population density was then computed by kernel density estimation (KDE) of a precomputed UMAP highlighting relative probabilities of HSPC lineage priming (KDE calculated using the *tl.embedding.density* function in Scanpy).

For HSPC lineage priming analyses which included the respective embryonic erythroid and erythroid terminal states, embryonic erythroid states were defined as any erythroid cell with a HBZ module *z*-score > 0, and erythroid as any erythroid cell with individual module *z*-score of HBA1, HBA2, HBG1, HBG2, HBD > 0.

### pySCENIC for regulon analysis

The pySCENIC package (v0.9.19) was used to identify transcription factors (TFs) and their target genes in the YS and iPSC scRNA-seq datasets. The ranking database (hg38 refseq-r80 500bp_up_and_100bp_down_tss.mc9nr.feather), motif annotation database (motifs-v9-nr.hgncm0.001-o0.0.tbl) and list of TFs (lambert2018.txt) were used. An adjacency matrix of TFs and their targets was generated. TF activity from the AUcell output was modeled along diffusion pseudotime rankings of each trajectory and used to train a nonlinear Generalized Additive Model (nlGAM) using the pyGAM.LinearGAM model to identify TF modules which significantly changed across each lineage pseudotime. A gridsearch of between 50 and 200 splines were calculated. Significantly changing TF regulons across pseudotime were classified with a *P*<0.05 and reported in Fig. 5, D and H, and data S30). Regulon matrix heatmaps were plotted using the *Seaborn* (v0.12.1) package in Python. Regulon scores were variance-scaled and min–max-standardized with a distribution of 0-1.

### Cell-cell interaction predictions using CellPhoneDB

To assign putative cell–cell interactions within the YS scRNA-seq dataset, we used CellPhoneDB (v2.1.2). Log-transformed, normalized, and scaled gene expression values for all cell states were exported. CellPhoneDB was run using the statistical method using the receptor-ligand database (v2.0.0) with a significance *P*-cut-off of 0.05 (data S28 to S29). Outputs were ranked by log-mean expression for interactions between cell types of interest in each analyses and plotted as a *z*-scored heatmap to show standard deviations from mean for each receptor–ligand pair.

### Hiplex RNAscope

Human YS tissue (8 PCW) was frozen in OCT compound (Tissue-Tek). 12-plex smFISH was performed using the RNAscope HiPlex v2 assay (ACD, Bio-Techne) on three cryosections (10 µm) per manufacturer’s instructions, using the standard pretreatment for freshly frozen samples and permeabilized with Protease III, for 15 min at room temperature. The imaging cycles, primary probes and label fluorophores were: *Cycle1_KLRB1_AlexaFluor488, Cycle1_CD1C_Dylight550, Cycle1_IL7R_Dylight650, Cycle1_SPINK2_AlexaFluor750, Cycle2_P2RY12_AlexaFluor488, Cycle2_TNFA_Dylight550, Cycle2_LGALS3_Dylight650, Cycle2_IL33_AlexaFluor750, Cycle3_PLVAP_AlexaFluor488, Cycle3_SPINK1_Dylight550, Cycle3_C1QA_Dylight650, Cycle3_ACTA2_AlexaFluor750, Cycle4_P2RY12_Opal570 and Cycle4_IBA1_Cy5.* Slides were counterstained with DAPI and coverslipped for imaging.

For protein validation, slides were fixed with 4% (w/v) paraformaldehyde (PFA) for 60 min at room temperature and then washed and dehydrated in an ethanol gradient (50 to 100%) for 5 min each. Sections were treated with Protease III (ACD, Bio-Techne) for 15 min at room temperature, then washed with PBS prior to blocking in 10% (v/v) normal donkey serum containing 1% (w/v) Triton X-100 and 0.2% (w/v) gelatin for 60 min at room temperature. Primary antibodies were incubated at 4°C overnight, then washed three times for 20 min each with a wash buffer (0.1% (w/v) Triton X-100 in PBS). Slides were blocked with HRP Block (ACD, Bio-Techne) for 60 min at room temperature, and washed with ACD Wash Buffer (ACD, Bio-Techne) prior to addition of secondary antibody and incubation for 60 min at room temperature. Slides were washed three times for 20 min each (0.1% (w/v) Triton X-100 in PBS). TSA-Opal570 was added for 10 min at room temperature, then washed three times with ACD Wash Buffer. Slides were counterstained with DAPI and coverslipped for imaging.

Imaging was performed on a custom two-camera spinning disk confocal microscope built around a Crest Optics X-light v3 module by Cairn Research, a scientific equipment manufacturer. The instrument was controlled using the Micro-Manager software (71). All imaging was performed in spinning disk confocal mode with a 40X water immersion objective (NA 1.15, 180nm/pixel) and 1.5-µm *z*-step using Prime BSI Express (Teledyne Photometrics) camera.

### RNAscope image analysis

Before each imaging experiment, a slide covered in a sparse layer of 0.5-µm Tetraspeck beads was imaged in all channels. The bead images in all channels were then registered against the beads in the DAPI channel and their respective affine transforms were saved.

After imaging, each individual tile was *z*-projected with a maximum intensity projection, then the channels were transformed using the saved affine transforms. The projected, transformed tiles were saved back to a temporary directory along with a bigstitcher-compatible XML file. The BigStitcher software (72) was then used to stitch the transformed tiles together and the final stitched image exported for further analysis.

All imaging cycles for a given tissue section were registered in two steps. First, we used feature registration algorithm implemented in Python via OpenCV-contrib library (version 4.3.0) (73) to compute an affine transformation of DAPI channel from cycle r>1 (moving image) with respect to DAPI channel from the first cycle r=1 (reference image). Key points were detected using the FAST feature detector, whose surrounding areas were described using the DAISY feature descriptor, while the FLANN-based matcher was used to find correspondences between pairs of key points from reference and moving images and filter out unreliable points. The remaining key points were processed using the RANSAC-based algorithm that aligns them and estimates affine transformation parameters with four degrees of freedom.

For the second registration step, a nonlinear registration algorithm based on Farneback optical-flow available in Python via OpenCV library was used to achieve more accurate registration by warping images locally. Specifically, local warping was computed using the DAPI channel, from cycle r>1 with respect to the corresponding channel of the first round. The computational pipeline implementing these registration steps was optimized so that it could be performed efficiently on large images. The corresponding code for feature registration is available at github.com/BayraktarLab/feature_reg, while the code for optical-flow registration at github.com/BayraktarLab/opt_flow_reg.

### Immunohistochemistry

Formalin-fixed, paraffin-embedded blocks of YS 4-8 PCW, embryonic liver 7-8 PCW, and healthy adult liver were sectioned at 4-µm thickness onto slides coated with 3-aminopropyltriethoxysilane (APES).

For hematoxylin and eosin staining, slides were dewaxed in xylene and rehydrated through graded ethanol, as previously described (10). Rehydrated slides were incubated for 5 min in Mayer’s hematoxylin (Dako, Agilent), rinsed in tap water and then differentiated for 2 s in acid alcohol before washing in tap water followed by Scott’s tap water substitute (Leica Biosystems). Sections were counterstained in triple eosin (Dako, Agilent) for 5 min before being rinsed in tap water, dehydrated through graded ethanol (70% to 99%), and then placed in xylene before mounting with DPX (Dako, Agilent).

For immunohistochemistry (IHC), dewaxing, rehydration, and staining was performed using the Discovery Ultra auto Stainer and kits (Ventana, Roche) following the manufacturer’s protocols. Primary and secondary antibodies and their concentrations are listed in data S23. Slides were counterstained with one drop of hematoxylin II (Ventana, Roche) for 8 min, rinsed with Reaction Buffer and one drop of Bluing reagent (Dako, Agilent) added for 4 min. The slide was then rinsed with a Reaction buffer, before being dehydrated by hand through graded ethanol (70% to 99%), placed in xylene and mounted with DPX (Dako, Agilent).

Rabbit polyclonal anti-human alpha-1-fetoprotein (AFP; Agilent) staining was performed by NovoPath, Newcastle upon Tyne NHS Trust, using a proprietary method.

For the Martius Scarlet eBlue (MSB) stain, slides were dewaxed in xylene and rehydrated through graded ethanol as previously published (10). Rehydrated slides were placed in Bouins’ fixative (Atom Scientific) for 1 hour at 60°C, washed in running water, incubated in Weigert’s solution (Atom Scientific) for 10 min and washed in water. Slides were differentiated in 0.9% ethanol for 1-2 s before rinsing in tap water followed by Scott’s tap water substitute (Leica Biosystems), distilled water and finally 95% ethanol. Slides were then incubated stepwise in Martius yellow (3 min) (Atom Scientific), Brilliant crystal scarlet (6 min) (Atom Scientific), and 50% (v/v) Methyl blue (2 min) (Atom Scientific), washing with distilled water between each stain. Slides were washed in tap water, rapidly dehydrated (2-3 min) through graded ethanol (70 to 99%), then placed in xylene before mounting with DPX mountant (Dako, Agilent).

All slides were imaged at 20X magnification on a NanoZoomer S360 (Hamamatsu) digital slide scanner. MSB stained images were deconvolved into respective Martius yellow, crystal scarlet and methyl blue channels using the Colour Deconvolution plugin (v1.8) (Masson Trichrome) in FIJI with thresholds set using the Otsu method. Pseudocolors for each deconvolved channel were then assigned as in Fig. 2C.

### ASGR1 and CD34 immunofluorescence microscopy

YS sections were baked onto slides for 2 hours at 60°C before being dewaxed in xylene and rehydrated through graded ethanol as previously described (10). Slides were washed with distilled water then placed in a pressure cooker with boiling citrate buffer pH 6 (10 mM citric acid (Sigma), 0.05% v/v Tween 20 (Sigma) in DI water) for 2 min for antigen retrieval. Slides were then washed for 3 min with distilled water followed by 3 min in PBS (Sigma). Sections were blocked with 20% (v/v) goat serum (R&D Systems) for 45 min at room temperature. Primary antibodies were diluted in blocking solution (data S23), added to the sections and incubated for 1 hour at room temperature. Slides were washed twice for 3 min each in a wash buffer (0.1% (w/v) Triton X (Sigma) in PBS), then twice for 3 min each in PBS. Secondary antibodies (see data S23) were diluted in blocking solution, added to section and incubated for 2 hours at room temperature. The wash step was repeated and then 300 nM DAPI (Sigma) was added. Slides were incubated for 5 min before washing with PBS. Slides were then mounted with ProLong™ Diamond Antifade (Thermofisher) and imaged on a Zeiss Axioimager with Zeiss ZEN pro software.

### SMA and LYVE1/CD34 immunofluorescence microscopy

PFA-fixed YS was cryoprotected with sucrose 10%, embedded in gelatin-sucrose solution (7.5% x/v gelatin (VWR 24350.262), 10% w/v sucrose (VWR27478.296), in 0.12M PBS), frozen at -50°C, then sectioned at 14µm. Slides were stored at -80°C until use, dried for 30 min, then blocked with PBS Gelatin Triton (0.2% w/v gelatin, 0.25% Triton X-100 (Sigma-Aldrich) in PBS) for 1 hour. Primary antibodies were diluted in blocking solution (data S23), added to the sections, and incubated overnight. Slides washed with PBS three times at 10-min intervals. Secondary antibodies were diluted in blocking solution and added to sections to incubate for 2 hours (data S23). Hoechst 33258 (Sigma-Aldrich) was added to the secondary antibody solution. Sections were washed with PBS three times at 10-min intervals, and coverslips were mounted with Mowiol (Calbiochem). Sections were imaged at 20X magnification on Leica DM6000 widefield microscope with MetaMorph software. Brightness and contrast were adjusted and a scale bar was added with FIJI (74).

### Light-sheet fluorescence microscopy

Candidate antibodies were screened by immunofluorescence on cryosections obtained from OCT-embedded specimens as previously described (10, 58). Routine light-sheet immunofluorescence microscopy (LSFM) was then performed on floating whole-mount yolk sacs as previously described, with primary antibody incubation reduced to 10 days and secondary reduced to 2 days, both at 37°C to preserve tissue integrity. Antibody and other reagents including nuclear marker TO-PRO-3 iodide are specified in data S23. Yolk sacs were embedded in 1.5% agarose blocks prior to solvent-based clearing as previously described (58). YS retained its spherical shape throughout the procedure. Imaging was performed as previously described in dibenzyl ether with a Miltenyi Biotec Ultramicroscope Blaze (sCMOS camera 5.5MP controlled by Inspector Pro 7.3.2 acquisition software), which generates light sheets at excitation wavelengths of 488, 561, 640, and 785 nm. Objective lenses of 4X magnification (MI Plan 4X NA0.35) and 12X magnification (MI Plan NA 0.53) were used. Imaris (v9.8, BitPlane) was used for image conversion, processing, and video production. Blender 3.0 was used to edit videos and add text. All raw image data are available on request (A.C. and M.H.).

### Statistics and reproducibility

The number of cells from each cell type in each de novo single cell dataset provided in this manuscript are provided in data S4.

## Supplementary Material

Table S1

Table S2

Table S3

Table S4

Table S5

Table S6

Table S7

Table S8

Table S9

Table S10

Table S11

Table S12

Table S13

Table S14

Table S15

Table S16

Table S17

Table S18

Table S19

Table S20

Table S21

Table S22

Table S23

Table S24

Table S25

Table S26

Table S27

Table S28

Table S29

Table S30

Table S31

Table S32

Table S33

MDAR Reproducibility Checklist

Movie S1

Movie S2

Supplementary Materials

## Data Availability

All novel raw sequencing data from this study are made publicly available at ArrayExpress as FASTQs and count matrices as follows: (i) Human embryonic liver and yolk sac 10X scRNA-seq (82); (ii) Human embryonic yolk sac 10X scRNA-seq (83); (iii) Human embryonic yolk sac Smart-seq2 scRNA-seq (84); (iv) Human embryonic yolk sac CITE-seq (85); (v) Human embryonic liver CITE-seq (86); (vi) Human fetal liver CITE-seq (87). Accessions for published data reused in this study are detailed comprehensively in data S6. Processed single-cell datasets are available for interactive exploration and download as well as corresponding trained scVI and logistic regression models via our interactive web portal (https://developmental.cellatlas.io/yolk-sac). Of note, data on portals are best used for rapid visualization. For formal analysis and all code for reproducibility including trained scVI VAE, ldVAE and trained logistic regression models, we recommended following our archived code available on Github (88) and our interactive web portal. All raw and processed imaging data are available on the EBI Bioimaging archive (89). Processed imaging data are available on our interactive web portal. For the purpose of Open Access, the author has applied a CC-BY public copyright license to any author-accepted version of this manuscript arising from this submission.

## One-Sentence Summary

The human yolk sac is a key staging post in a relay of vital organismal functions during human pregnancy.
