# Supplementary material for: Yolk sac cell atlas reveals multiorgan functions during human early development
Source: Science. Author manuscript; Available in PMC 2023 Aug 26. (PMC7614978; doi:10.1126/science.add7564)
Supplement: Supplementary Materials [file EMS185071-supplement-Supplementary_Materials.pdf]

Science AAAS

# Supplementary Materials for

## Yolk sac cell atlas reveals multiorgan functions during human early development

**Authors:** Issac Goh[1,2]†, Rachel A. Botting[1,2]†, Antony Rose[1,2]‡, Simone Webb[1,2]‡, Justin Engelbert[2], Yorick Gitton[3], Emily Stephenson[1,2], Mariana Quiroga Londoño[4], Michael Mather[2], Nicole Mende[4], Ivan Imaz-Rosshandler[4,5], Lu Yang[1], Dave Horsfall[1,2], Daniela Basurto-Lozada[1,2], Nana-Jane Chipampe[1], Victoria Rook[1], Jimmy Tsz Hang Lee[1], Mai-Linh Ton[4], Daniel Keitley[1,6], Pavel Mazin[1], M.S. Vijayabaskar[4], Rebecca Hannah[4], Laure Gambardella[1], Kile Green[7], Stephane Ballereau[1], Megumi Inoue[3], Elizabeth Tuck[1], Valentina Lorenzi[1], Kwasi Kwakwa[1], Clara Alsinet[1,8], Bayanne Olabi[1,2], Mohi Miah[1,2], Chloe Admane[1,2], Dorin-Mirel Popescu[2], Meghan Acres[2], David Dixon[2], Thomas Ness[9], Rowen Coulthard[9], Steven Lisgo[2], Deborah J Henderson[2], Emma Dann[1], Chenqu Suo[1], Sarah J. Kinston[4], Jong-eun Park[10], Krzysztof Polanski[1], John Marioni[1,11,12], Stijn van Dongen[1], Kerstin B. Meyer[1], Marella de Bruijn[13], James Palis[14], Sam Behjati[1,15], Elisa Laurenti[4], Nicola K. Wilson[4], Roser Vento-Tormo[1], Alain Chédotal[3], Omer Bayraktar[1], Irene Roberts[16], Laura Jardine[1,2]*, Berthold Göttgens[4]*, Sarah A. Teichmann[1,17]*, Muzlifah Haniffa[1,2,18]*

Correspondence to: mh32@sanger.ac.uk

**This PDF file includes:**

Figs. S1 to S15
Captions for movies S1 and S2
Captions for data S1 to S33

**Other Supplementary Materials for this manuscript include the following:**

Movies S1 and S2
Data S1 to S33

# Supplementary Figure 1

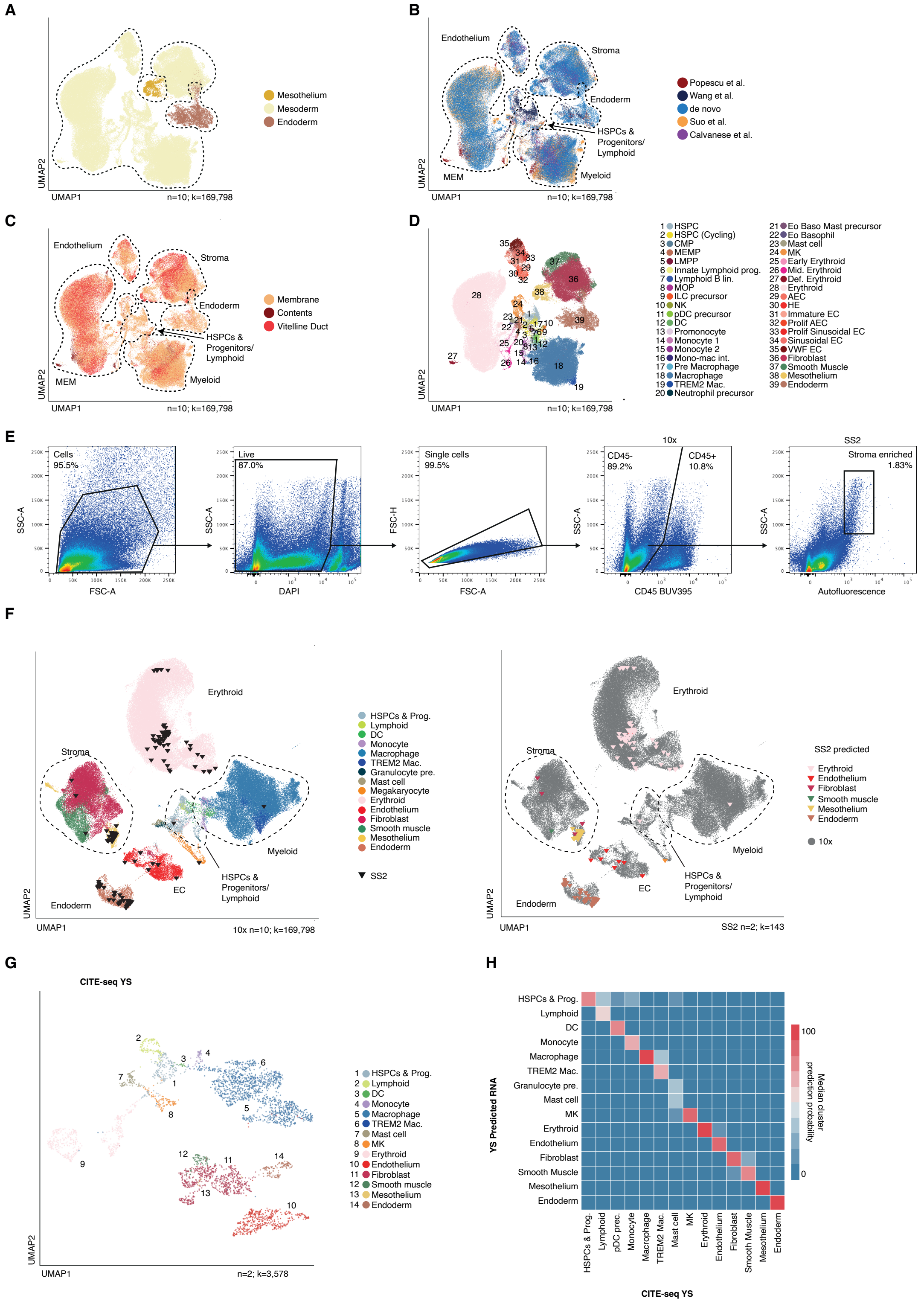

**Fig. S1. A single-cell atlas of the human yolk sac.** (**A** to **D**) UMAP visualization of scRNAseq data shown in Fig. 1C colored according to yolk sac (YS) tissue layer (**A**), data source (**B**), anatomical structure (**C**), and refined annotation (**D**). HSPC: hematopoietic stem/progenitor cell; CMP: common myeloid progenitor; MEMP: megakaryocyte–erythroid–mast cell progenitor; LMPP: lymphoid-primed multipotent progenitor; Prog.: progenitor; MOP: monocyte progenitor; ILC: innate lymphoid cell; NK: natural killer cell; pDC: plasmacytoid DC; pre.: precursor; DC: dendritic cell; Mac.: macrophage; Mono-mac int.: monocyte macrophage intermediate; Eo: eosinophil; Baso: basophil; MK: megakaryocyte; AEC: arteriolar endothelial cell; HE: hemogenic endothelium; EC: endothelial cell (data S3, S4, S5, and S17). (**E**) FACS gating strategy used to sort YS CD45$^{+/-}$ fractions for plate-based scRNA-seq. Sequential gates show selection of CD45$^{-}$SSC$^{hi}$ autofluorescent cells, enriched in non-erythroid stromal populations. Representative gating from n=2 independent samples (5-7 PCW) (data S1). (**F**) UMAP visualization of the YS cells shown in Fig. 1C (n=10, k=169,798) integrated with k=143 cells from plate-based sequencing (SS2) cells (triangles) FACS-isolated from n=2 individual donors (5-7 PCW). Left: colors indicate cell states in droplet based scRNA-seq (10X). Right; colors indicate predicted cell states in plate-based scRNA-seq (SS2) (data S5 and S18). (**G**) UMAP of YS cells from CITE-seq data, intersected between RNA and protein modalities batch corrected using TotalVI from n=2 biologically independent samples (n=2, k=3,578). Colors represent broad cell states (data S4, S5, and S19). (**H**) Heatmap of class prediction probabilities for a logistic regression model (Elasticnet) trained on YS scRNA-seq cell states (*y*-axis) and projected onto corresponding cluster-derived cell states in YS CITE-seq data (*x*-axis) (data S9). Color scale indicates median probabilities.

# Supplementary Figure 2

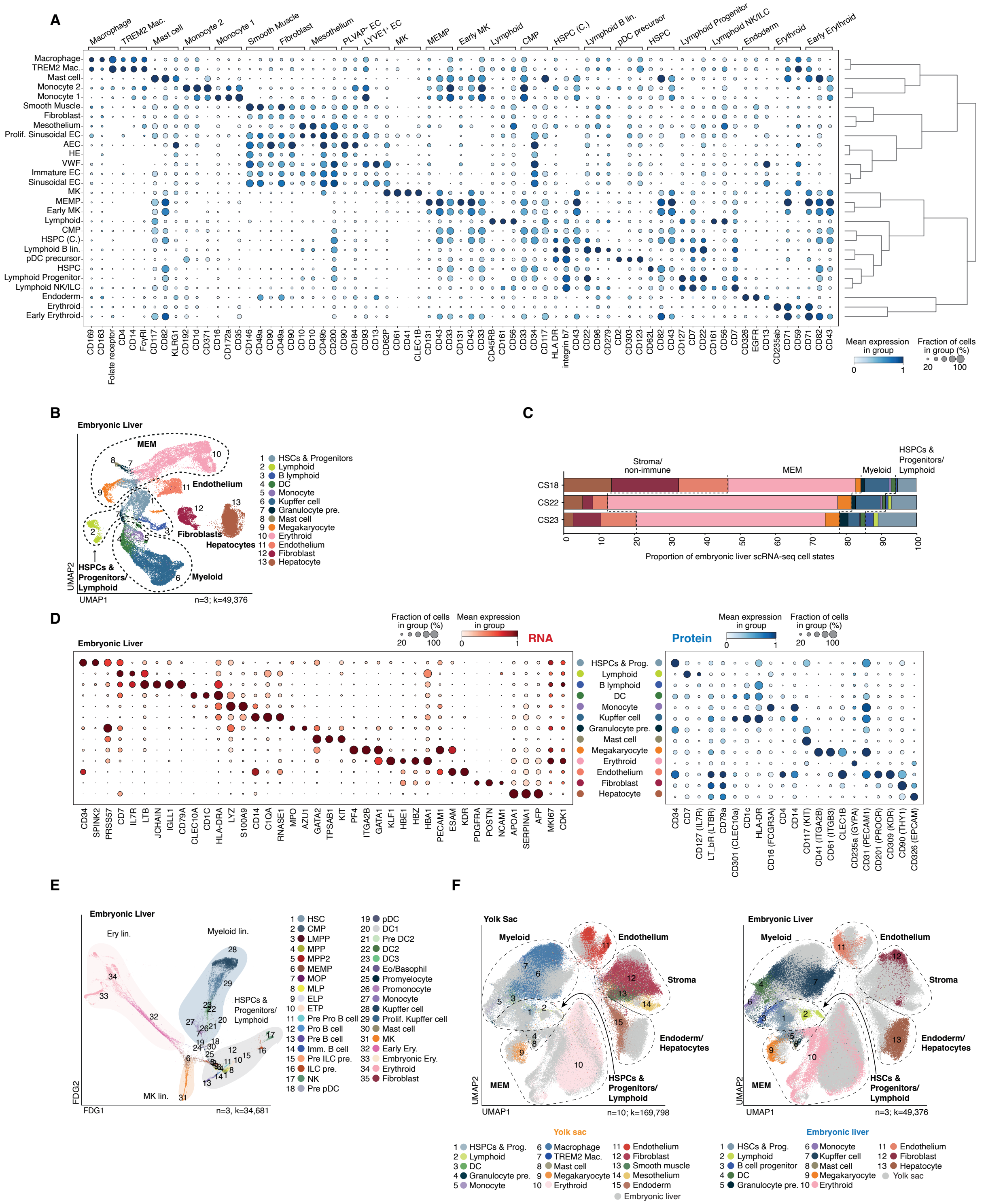

**Fig. S2. CITE-seq analysis of human yolk sac and embryonic liver.** (**A**) Dot plot showing the mean expression (color scale) and the fraction of cells expressing each protein (dot size) of surface proteins in YS CITE-seq data analyzed using totalVI. Cell states were grouped using a hierarchical dendrogram. Marker proteins were derived using a one-vs-all TotalVI differential expression test (*P*<0.05, Bayes factor >0.95, Median LFC >0.51) (more details on this approach are available in the revised methods section under 'Clustering and annotation of scRNA-seq and CITE-seq data'). Data are min-max-standardized with a distribution of 0-1. (data S19 and S20). (**B**) UMAP visualization of the cell states identified in the embryonic liver (EL) scRNA-seq dataset from n=3 independent biological repeats (k=49,376, CS18-23). Colors represent cell states. DC: dendritic cell; MEM: megakaryocyte-erythroid-mast cell lineage; pre.: precursor (data S21). (**C**) Stacked bar chart displaying the proportional representation of broad cell states in EL scRNA-seq data by sample. Colors match cell states shown in (B). (**D**) Dot plot showing the mean expression (by color) and the fraction of cells expressing each gene or protein (by dot size) of broad cell state-defining genes in EL scRNA-seq data (n=3, k=49,376) (left), and their protein counterparts in EL CITE-seq dataset (n=9 biologically independent samples, k=57,310, CS16-17 PCW) (right) (data S4). Data are min-max-standardized with a distribution of 0-1. (**E**) Force-directed graph (FDG) visualization of hematopoietic cell states identified in EL scRNA-seq from n=3 biologically independent donors (k=34,681). Colors represent cell states and clouds represent lineages. CMP: common myeloid progenitor; DC: dendritic cell; ELP: early lymphoid progenitor; Eo./Baso.: eosinophil/basophil; Ery.: erythroid; ETP: early thymic progenitor; HE: hemogenic endothelium; HSC: hematopoietic stem cell; HSPC: hematopoietic stem progenitor cell; ILC: innate lymphoid cell; LMPP: lymphoid-primed multipotent progenitor; Mac: macrophage; MEM: megakaryocyte-erythroid-mast cell lineage; MEMP: megakaryocyte-erythroid-mast cell progenitor; MK: megakaryocyte; MLP: multi-lymphoid progenitor; Mono: monocyte; MOP: monocyte progenitor; MPP: multipotent progenitor; Neut: neutrophil; NK: natural killer cell; pDC: plasmacytoid DC; pre.: precursor; prog.: progenitor; prolif.: proliferating (data S4, S5, and S17). (**F**) UMAP visualization of the merged YS and EL scRNA-seq data shown in Fig. 1B and fig. S3B, respectively, colored by cell state and tissue (Left, YS, n=10, k=169,798; Right, EL, n=3, k=49,376) (data S4, S5, and S17).

# Supplementary Figure 3

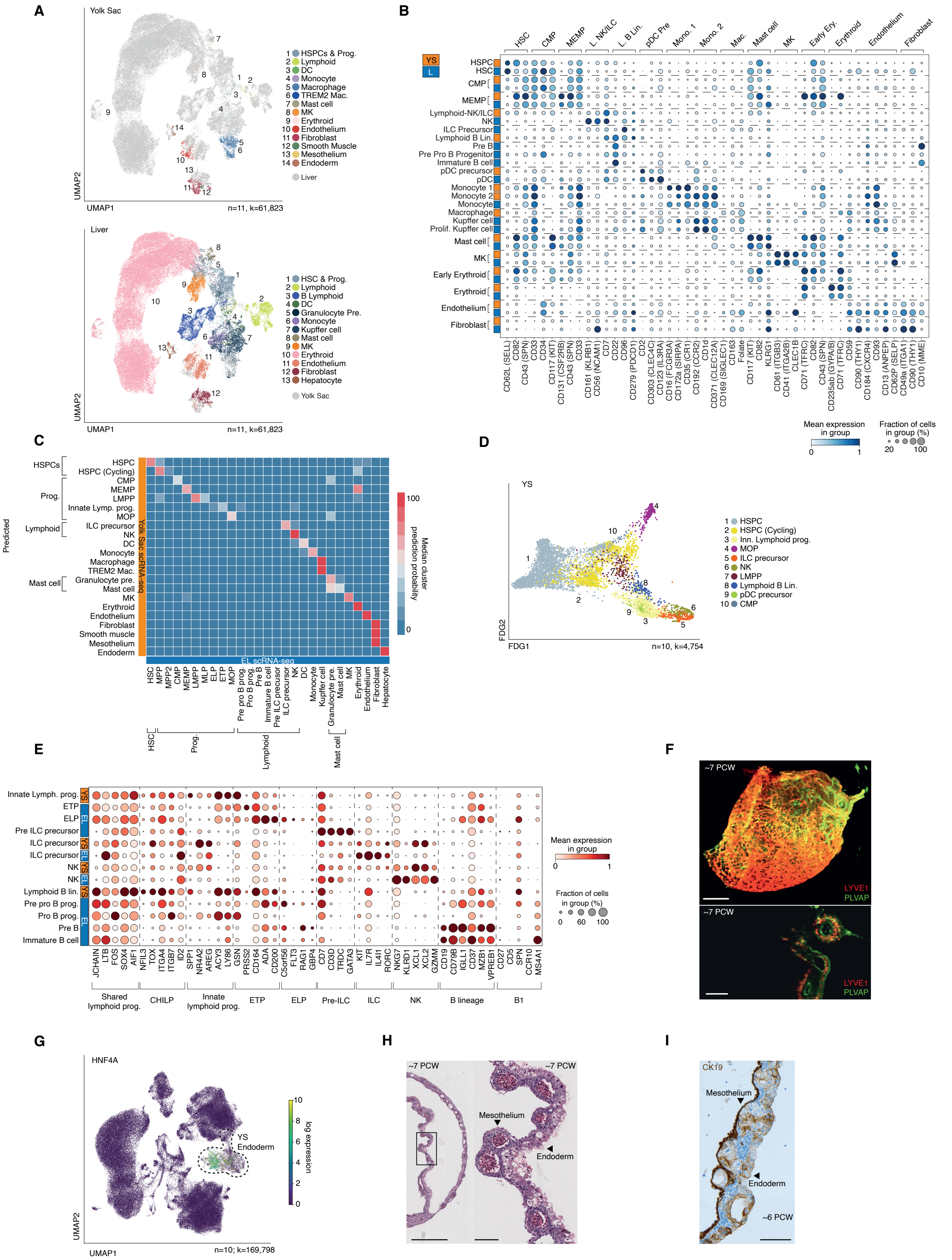

**Fig. S3. Comparison between yolk sac and embryonic liver cell states and yolk sac anatomy.** (**A**) UMAP visualization of combined YS (top) and liver (bottom) CITE-seq datasets (n=11, k=61,823). Colors represent cell states. Colors represent cell states (data S4, S5, S19, and S22) (**B**) Dot plot showing the mean expression (by color) and the fraction of cells expressing each protein (by dot size) of proteins derived from fig. S2A for each matched refined cell state in the YS and liver CITE-seq datasets. The equivalent gene symbol is shown in parentheses when different from the protein. HSC: hematopoietic stem cell; HSPC: hematopoietic stem and progenitor cell; CMP: common myeloid progenitor; MEMP: megakaryocyte–erythroid–mast cell progenitor; pDC: plasmacytoid dendritic cell; MK: megakaryocyte (Data are min-max-standardized with a distribution of 0-1) (data S19 and S22). (**C**) Heatmap of class prediction probabilities for a logistic regression model (Elasticnet) trained on embryonic liver (EL) scRNA-seq cell states (*x*-axis) and projected onto corresponding YS scRNA-seq cell states (*y*-axis) (data S12). Color scale indicates median probabilities. Brackets indicate broad cell-state groups as shown in (A). (**D**) Force directed graph (FDG) visualization of lymphoid cell states in the YS scRNA-seq dataset (n=10, k=4754) (data S5). (**E**) Dot plot showing the mean expression (color scale) and the fraction of cells expressing each gene (by dot size) of lymphoid marker genes in lymphoid lineage cell states in the YS and EL scRNA-seq datasets. Data are min-max-standardized with a distribution of 0-1. (**F**) Representative images of 3D (top) and 2D *z*-stack (bottom) light-sheet fluorescence microscopy images of 7-PCW YS stained with anti-LYVE1 (red) and anti-PLVAP antibodies (green). Scale bars: 700 µm (top) and 100 µm (bottom). See movie S2 and data S23**.** (**G**) Feature plot of *HNF4A* expression in YS scRNA-seq data (Fig. 1C) log-normalized and scaled max expression value=10 (n=10, k=169,798) (data S5). (**H**) Histology of a 7-PCW YS, demonstrated by hematoxylin and eosin staining of a formalin-fixed paraffin-embedded tissue section. A representative image from one of n=4 biologically independent samples (4-8 PCW) is shown, with mesothelium and endoderm marked by arrows. Scale bars: 1 mm (left) and 100 µm (right). (**I**) Immunohistochemistry stain for CK19 (brown) in a 7-PCW YS, representative image from one of n=4 biologically independent samples. Scale bar: 100 µm (data S23).

# Supplementary Figure 4

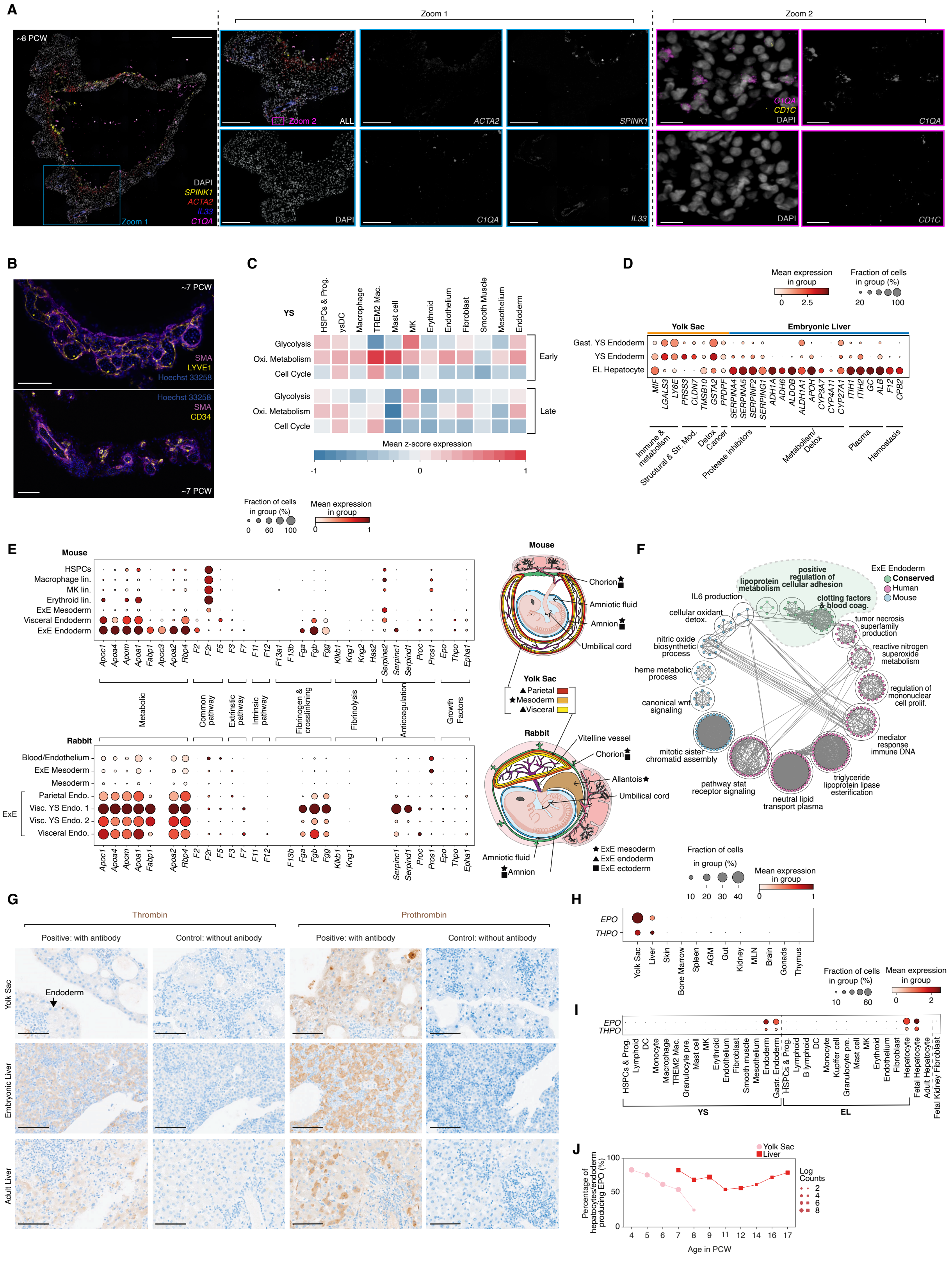

**Fig. S4. Multiorgan functions of the yolk sac.** (**A**) RNAscope imaging of an 8-PCW YS with probes specific for endoderm (*SPINK1*), smooth muscle (*ACTA2*), arteriolar endothelial cells (*IL33*), macrophages (*C1QA*), and dendritic cells (*CD1C*; right "Zoom 2" panels only), costained with DAPI. Blue box ("Zoom 1") and magenta box ("Zoom 2") indicate ROIs shown in Fig. 1F. "Zoom 1" and "Zoom 2" panels show greyscale images of the individual channels in Fig. 1F. Left scale bar: 500 µm; middle "Zoom 1" scale bars: 200 µm; right "Zoom 2" scale bars: 50 µm (data S23). (**B**) Lightsheet immunofluorescence imaging of 7-PCW YS stained with anti-SMA (magenta) and anti-LYVE1 (yellow; top) or anti-CD34 (yellow, bottom) antibodies, costained with Hoechst 33268 (blue) and imaged using confocal microscopy. Scale bar: 100 µm (data S23). * indicates vessels. (**C**) Heatmap of *z*-normalized GO geneset module scores between early and late predicted Milo neighborhoods for cell cycle (GO:0022402), oxidative metabolism (GO:0045333), and glycolysis (GO:0006096), subtracted by the mean expression of 200 randomly sampled genes at 50 bins (data S24). (**D**) Dot plot showing the mean expression (color scale) and the fraction of cells expressing each gene (by dot size) of selected DEGs (data S3, S7, S21, and S26) between YS endoderm (main and gastrula (gast.) data) and embryonic liver (EL) hepatocytes (data scaled max_value=10, gastrulation data scaled independently). Brackets indicate curated enriched GO annotations for each set of genes. Str. Mod.: Structural modification. (**E**) Left: Dot plots showing the mean expression (color scale) and the fraction of cells expressing each gene (by dot size) of clotting and soluble factors in relevant cell states from mouse gastrulation scRNA-seq data (*75*) (top) and from rabbit scRNA-seq data (*55*) (bottom). Data are min-max-standardized with a distribution of 0-1. Right: illustrations of developing mouse (~E9.5) and rabbit (~GD9) embryos. Text legends indicate corresponding extraembryonic anatomical regions between species, whereas shapes indicate germ layer origin of the anatomical regions. Star: mesoderm; triangle: endoderm; and square: ectoderm. Layers of the YS are delineated by color. Red: parietal; orange: mesoderm; and yellow: visceral. (**F**) Flower plot of the significant gene sets enriched in YS endoderm (pink), mouse extraembryonic (ExE) endoderm (blue), and conserved between species (green). Nodes indicate significantly enriched gene sets (Q-value <0.05), whereas edges between nodes represent gene overlap between gene sets. Annotated grouping circles indicate Markov cluster neighborhoods of gene expression modules which share high gene-set similarities (data S25). (**G**) IHC antibody staining of thrombin (F2) (column 1), prothrombin (column 3), and the respective controls without antibody (columns 3 and 4) in 7-PCW YS (top), 7-PCW EL (middle), and healthy adult liver (bottom). Representative images from 1 of n=3, 3, and 3 biologically independent YS (4-7 PCW), ELs (7-12 PCW) and adult livers, respectively. Protein (brown) and nuclei (blue). Scale bar: 100 µm (data S23). (**H**) Dot plot showing the mean expression (color scale) and the fraction of cells expressing each gene (dot size) of Endoderm-derived soluble factors between YS endoderm (main and gastrula (gast.) data) and embryonic liver (EL) hepatocytes, and non-immune cells from skin, bone marrow, spleen, aorta–gonad–mesonephros (AGM), gut, kidney, mesenteric lymph nodes (MLN), brain, gonads, and thymus. Data are min-max-standardized with a distribution of 0-1. (**I**) Dot plot showing the mean expression (color scale) and the fraction of cells expressing each gene (dot size) of soluble factors in YS (main and gastrula), EL, fetal/adult liver, and fetal kidney scRNA-seq select cell states (each dataset scaled max_value=10 independently then combined except YS and EL scRNA-seq). (**J**) Line graph showing the relative change in proportion of endoderm and hepatocyte cell states in YS and liver, respectively (*y*-axis), enriched in expression of EPO. Pink: human YS main and gastrulation scRNA-seq data. Red: matched Liver scRNA-seq data.

# Supplementary Figure 5

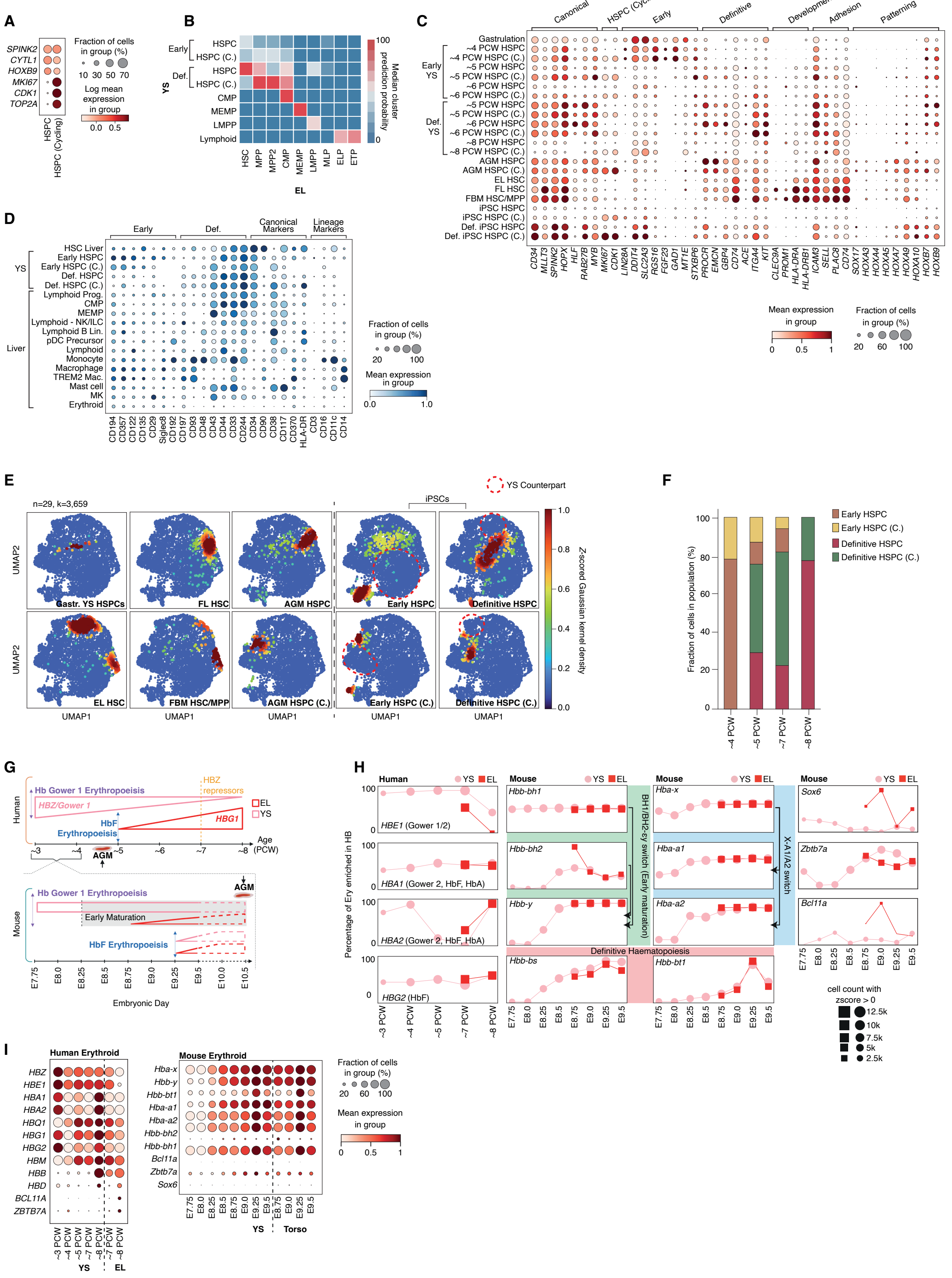

**Fig. S5. Early versus definitive hematopoiesis in yolk sac and liver.** (**A**) Dot plot showing the mean expression (color scale) and the fraction of cells expressing each gene (dot size) of genes distinguishing YS HSPC from YS cycling HSPC in the YS (main) scRNA-seq data (data scaled max_value=10). (**B**) Median ElasticNet logistic regression class prediction probabilities for a model trained on EL progenitor scRNA-seq cell states (*x*-axis) projected onto YS scRNA-seq cell states (*y*-axis) (data S12). (**C**) Dot plot showing the mean expression (color scale) and the fraction of cells expressing each gene (dot size) of canonical, cycling HSPC-specific, early, definitive, developmental-stage specific, adhesion and patterning HSC markers expressed between YS HSPCs (split by HSPC/ cycling HSPC and early/definitive) across time including gastrulation (67), AGM HSPC (66), matched EL HSC, FL HSC (*10*), fetal BM HSC/MPP (*35*), iPSC-derived HSPC (*20*), and definitive iPSC-derived HPSC (*12*)**.** Data are min-max-standardized with a distribution of 0-1. (**D**) Dot plot showing the mean expression (color scale) and the fraction of cells expressing each protein (dot size) of differentially expressed proteins between early and definitive HSPCS, alongside canonical HSC markers and lineage markers, in selected cell states from YS and liver CITE-seq data. Data are min-max-standardized with a distribution of 0-1. (data S27) (**E**) Density plots showing the distribution of indicated HSPC populations in the integrated UMAP landscape of HSPC/HSCs from the following scRNAseq datasets: YS (n=10, k=2,597), YS gastrulation (*14*) (n=1, k=23), AGM (*76*) (n=3, k=182), matched embryonic liver (EL) (n=3, k=412), fetal liver (FL) (n=14, k=242), fetal bone marrow (*35*) (FBM) (n=9, k=92), iPSC-derived HSPC (n=12, k=355) (*20*) and definitive iPSC-derived HSPC (n=2, k=273) (*12*). The color of HSC/HSPCs represents the *Z*-scored kernel density estimation (KDE) score for each population (data S5). Red dashed lines indicate the embedded positions of corresponding YS HSPC populations as shown in Fig. 3C. (**F**) Bar chart showing the proportional representation of early YS HSPC and cycling HSPC to definitive YS HSPC and cycling HSPC in the main and gastrulation YS scRNA-seq data (grouped by gestational age in PCW). (**G**) Schematic diagram showing the relative timescales of early and definitive erythropoiesis in human and mouse, and contributions of AGM, EL, and YS to this process. (**H**) Left column: Line graphs showing the relative change in expression of Gower 1/2 globin *HBE1*, Gower 2 globins *HBA1/2* and definitive globin *HBG2* in human erythroid cells from YS (pink) and matched embryonic liver (EL) (red) over gestational age. Central and right columns: Globin expression in mouse erythroid cells (*75*) including HBB BH1, εγ, X, A2, BT1 and BS. Pink lines: mouse YS scRNA-seq data. Red lines: aged-matched mouse torso scRNA-seq data. Mouse hemoglobins implicated in primitive maturation, definitive hematopoiesis, and a switch between the two are grouped. The *y*-axis represents the proportion of erythroid lineage cells. (**I**) Dot plot showing the mean expression (color scale) and the fraction of cells expressing each gene (dot size) of Hb genes in erythroid lineage cells, grouped by gestational age. Left: human YS scRNA-seq (main and gastrulation) and EL data. Right: mouse YS and aged-matched torso scRNA-seq data (*75*). Data are min-max-standardized with a distribution of 0-1.

# Supplementary Figure 6

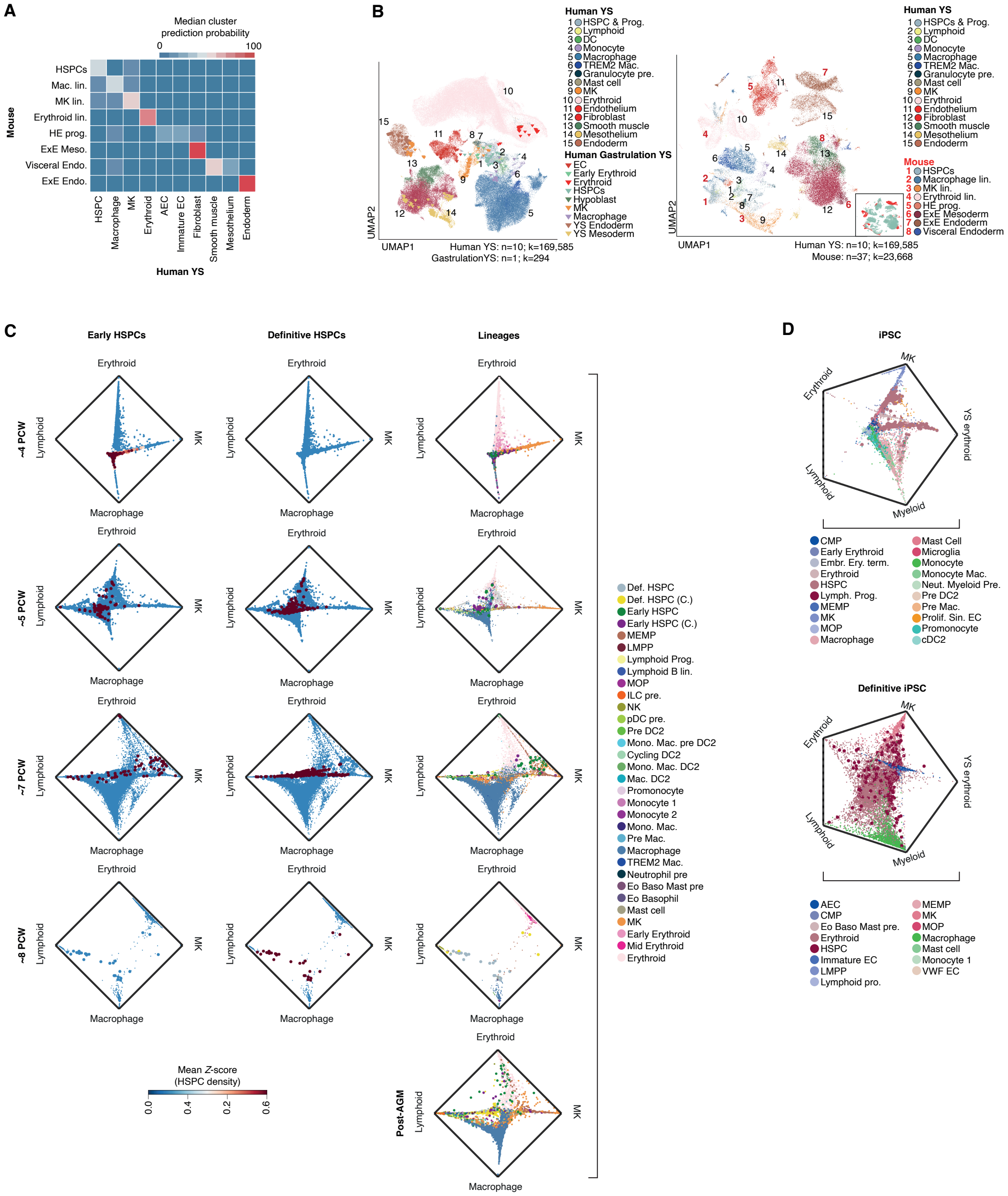

**Fig. S6. Hematopoietic waves in human yolk sac.** (**A**) Heatmap of class prediction probabilities for a LR model (Elasticnet) trained on human YS scRNA-seq cell states (*x*-axis) projected onto corresponding mouse extraembryonic cell states from mouse gastrulation dataset (*75*) (*y*-axis). Prog.: progenitor; AEC: arteriolar endothelial cell; EC: endothelial cell; HSPC: hematopoietic stem and progenitor cell (data S11). Color scale indicates median probabilities. (**B**) Left: UMAP visualization of matched hematopoietic cell states in human YS scRNA-seq (dots) as shown in Fig. 1C (n=10, k=169,798) integrated with human gastrulation (CS7) scRNA-seq data (*14*) (triangles) (n=1, k=91). Lin.: lineage; pre.: precursor; DC: dendritic cell; MK: megakaryocyte; EC: endothelial cell (data S5). Right: UMAP visualization of human YS scRNA-seq (as shown in Fig. 1C) and equivalent mouse gastrulation extraembryonic cell states (*75*) (n=36, k=139,331). Insert highlighting location of mouse cell states within UMAP. Colors represent cell states. ExE.: extra embryonic; lin.: lineage; HE: hemogenic endothelium. Inset colored by species (mouse: red; human: teal). (**C**) Radial plots showing relative probabilities of lineage-state transition between HSPCs and lineage-specific cell states starting from early HSPC (left) and definitive HSPC (middle). Right-hand column shows cell state annotations. Plots are segregated by gestational stages between CS10-11, CS14-15, CS17-18 and CS22-23. Color indicates the HSPC population density as a *z*-scored kernel density estimation (KDE) score and the position of HSPC population densities indicate respective lineage priming probability between macrophage, lymphoid (NK and B lineage), erythroid and MK terminal states. (**D**) Radial plots showing relative probabilities of lineage-state transition between iPSC-derived HSPCs from the culture protocol optimized for macrophage differentiation (top) and from the definitive iPSC culture protocol (bottom). Color indicates the cell-state annotation and the position of each population indicates respective lineage priming probability between myeloid, lymphoid erythroid, embryonic erythroid and MK terminal states.

# Supplementary Figure 7

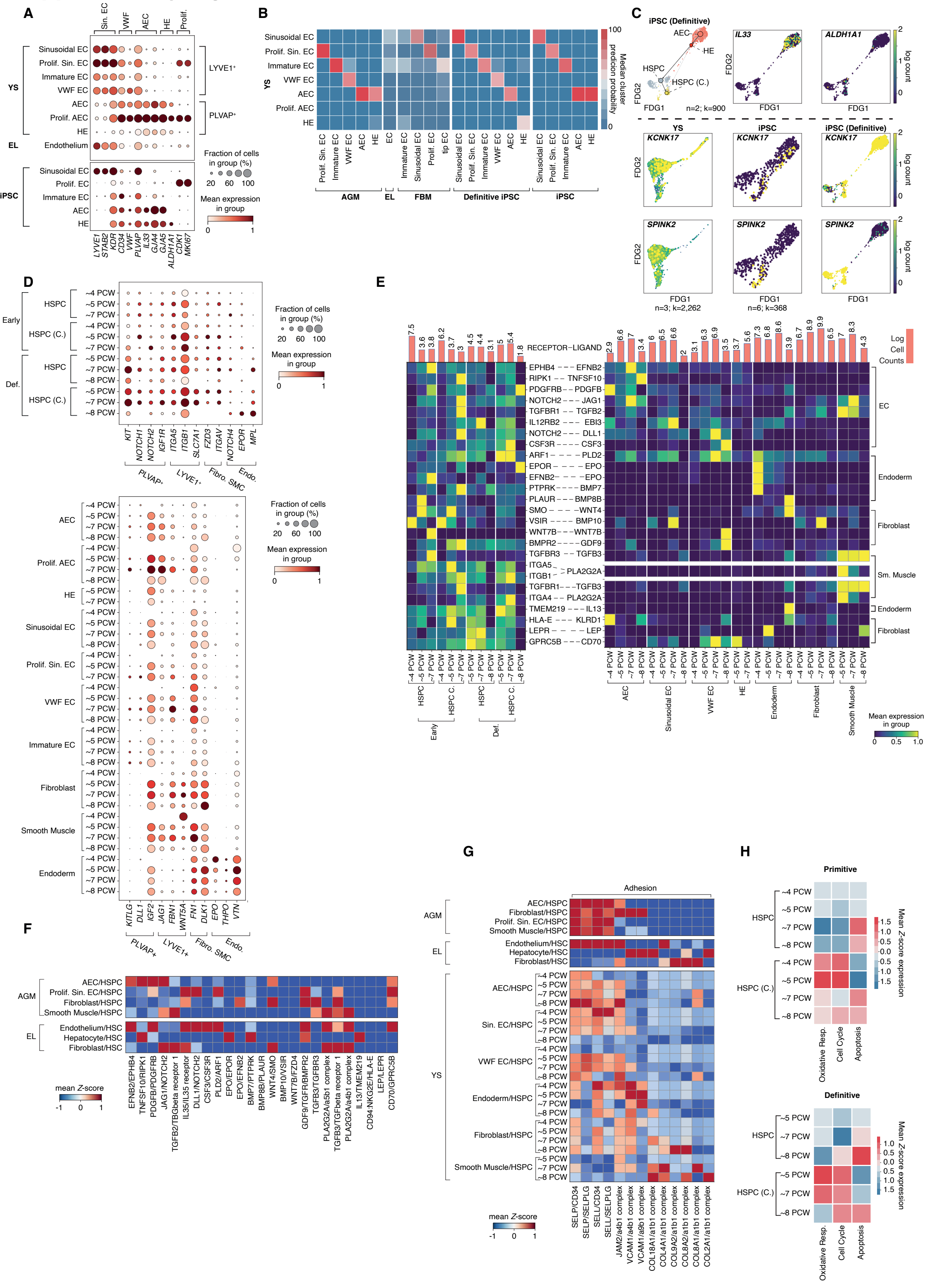

**Fig. S7. The lifespan of yolk sac HSPCs.** (**A**) Dot plot showing the mean expression (color scale) and the fraction of cells expressing each gene (dot size) of genes distinguishing endothelial cell subsets in YS (main), matched EL scRNA-seq and iPSC (*20*) scRNA-seq datasets. Data are min-max-standardized with a distribution of 0-1. (**B**) Heatmap of class prediction probabilities for a logistic regression model (ElasticNet) trained on YS endothelial cell (EC) states (*y*-axis) projected onto EC states in AGM (*32*, *76*), matched EL, and FBM (*35*), iPSC (*20*) and definitive iPSC (*12*) (*x*-axis) (vmin=0, vmax=1) (data S14). Color scale indicates median probabilities. (**C**) Top: Force directed graph overlaid with partition-based approximate graph abstraction (PAGA) map showing the trajectory of hemogenic endothelium (HE) transition to HSPC in definitive iPSC scRNA-seq data (*12*) (n=3, k=2262) with feature plots of key genes (*IL33*, *ALDH1A1*) involved in endothelial to hemogenic transition (data S5; as shown in Fig 4B for YS and iPSC). Bottom: Feature plots of key genes in endothelial to hemogenic transition (S*PINK2, KCNK17*) in YS, iPSC and definitive iPSCs scRNA-seq trajectories. (**D**) Dot plot showing the mean expression (color scale) and the fraction of cells expressing each gene (dot size) of genes predicted by CellphoneDB to form statistically significant ($P$<0.05) protein-protein interactions between HSPCS (top plot) and stromal cells (bottom plot) arranged by gestational age. Brackets indicate genes which form complexes (data scaled max_value=10) (data S29). Data are min-max-standardized with a distribution of 0-1. Fibro.: fibroblast, Endo.: endoderm. (**E**) Heatmaps showing mean (standardized) expression of curated and statistically significant ($P$<0.05) CellphoneDB putative receptor ligand interactions which change across time. Left: HSPC receptors; right: stromal ligands. Both are grouped by gestational age (data S29). Data are min-max-standardized with a distribution of 0-1 (standard-scale='var'). Log normalized cell counts are shown as a barplot above each cell-state column. Sm.: smooth (**F**) Heatmap showing relative mean expression *Z*-scores of curated CellphoneDB putative receptor ligand interaction matching S7E between stromal and HSC/HSPC subsets in AGM (top) and liver (bottom) (data S29). Sin.: sinusoidal (**G**) Heatmap showing relative mean expression *z*-scores of curated and statistically significant ($P$<0.05) CellphoneDB putative curated functional adhesion receptor ligand interactions between AGM (top), YS (middle) and EL (bottom) stromal subsets vs HSPC across gestation (data S29). (**H**) Heatmaps showing mean *z*-scored expression of metabolic (GO-ontology, GO-0045333), cell cycle (GO-ontology, GO:0022402) and apoptosis (GO-ontology GO:0006915) modules for early (left) and definitive (right) YS HSPC and cycling HSPC across gestational age (Module enrichment computed against 200 randomly sampled background genes at 50 bins). Resp.: respiration.

# Supplementary Figure 8

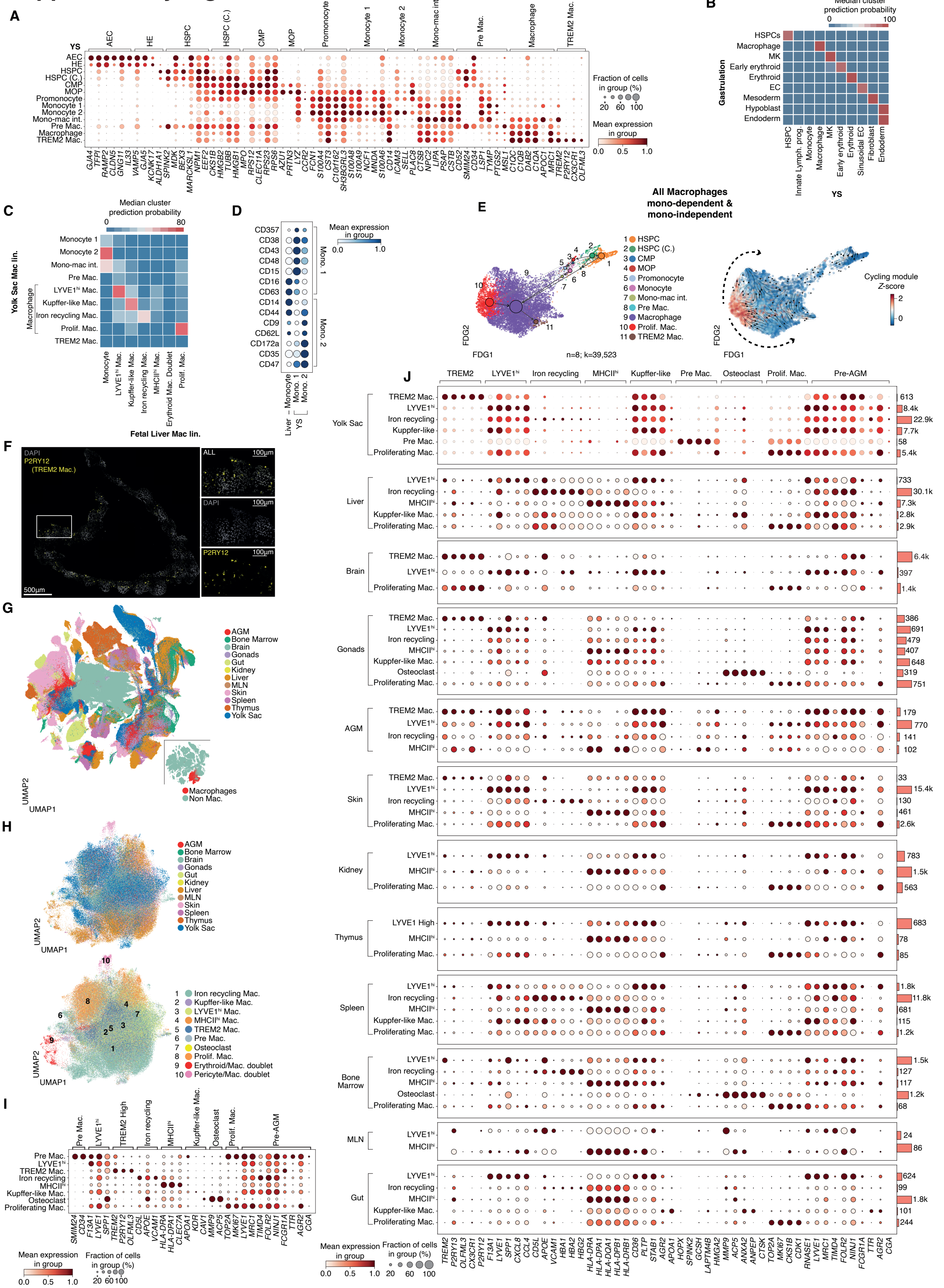

**Fig. S8. Macrophage subsets in human yolk sac and prenatal organs.** (**A**) Dot plot showing the mean expression (color scale) and the fraction of cells expressing each gene (dot size) of significant differentially expressed myeloid lineage gene markers ($P<0.05$) in the YS scRNA-seq. Differential expression was derived via a two-sided Wilcoxon rank-sum test (thresholded at expression in >25% of class, LFC >0.25 and Benjamini–Hochberg corrected $P<0.05$) (data S3 and S31). Data are min-max-standardized with a distribution of 0-1. (**B**) Heatmap of class prediction probabilities for a LR model (Elasticnet) trained on YS scRNA-seq cell states (*x*-axis) and projected onto cell states in human gastrulation scRNA-seq data (*14*) (*y*-axis). This LR was performed after reannotating human gastrula data in-house (data S15). Color scale indicates median probabilities. (**C**) Heatmap of class prediction probabilities for a LR model (Elasticnet) trained on expanded YS scRNA-seq Macrophage lineage cell states (*y*-axis) and projected onto Macrophage lineage cell states in human Fetal liver scRNA-seq data (7-17 PCW) (*14*) (*x*-axis) (data S12). Brackets indicate macrophage fractions resolved from the YS macrophages shown in Fig. 5C. Color scale indicates median population probability between 0-80. (**D**) Dot plot showing the mean expression (color scale) and the fraction of cells expressing each protein (dot size) of proteins in monocytes from embryonic liver and matched YS CITEseq datasets (data S20). Differentially expressed proteins and proteins matched to RNA markers from Fig. 5A are shown. (**E**) Left: Force directed graph (FDG) visualization overlaid with directional partition-based approximate graph abstraction (PAGA) map showing the trajectory of macrophage differentiation is YS scRNAseq data (n=8, k=39,523, CS10-CS23). Right: FDG visualization colored by *z*-score of enrichment in cycling module (GO:0007049) genes and overlaid with arrows inferred from a CellRank state transition matrix indicating the trend of trajectory. Dashed arrows indicate predicted trajectories of cycling macrophages into macrophage and TREM2 macrophage populations. (Module enrichment computed against 200 randomly sampled background genes at 50 bins). (**F**) Immunofluorescence images of an 8-PCW YS stained with anti-P2RY12 antibody to demarcate TREM2 macrophages and anti-IBA1 antibody to demarcate macrophages, co-stained with DAPI (n=1). White box indicates ROI shown. Scale bars: 500 µm and 100 µm (inset) (data S23). (**G**) UMAP of the integrated 12-organ fetal atlas ($k=3.12\times10^6$, n=150), colored by organ. Inset indicates the position of macrophages (teal) and non-macrophages (red). Organs include: YS (n=10, k=169,494), AGM (n=4, k=12,248), skin (n=13, k=178,563), brain (n=72, $k=2.16\times10^6$), gonads (n=44, k=14,244), thymus (n=11, k=104,251), gut (n=5, k=79,435), kidneys (n=4, k=26,372), liver (n=14, k=210,549), spleen (n=10, k=127,186), bone marrow (n=8, k=93,677), and MLN (n=2, k=6039)(data S6 and S7). (**H**) Feature plot showing VAE latent-space derived UMAP representation of macrophages across the integrated 12 organ fetal atlas colored by organ (top) and by annotated heterogenous macrophage substates (bottom). (**I**) Dot plot showing the mean expression (color scale) and the fraction of cells expressing each gene (dot size) of marker genes by macrophage subsets across the 12-organ fetal atlas. Data are min-max-standardized with a distribution of 0-1. (**J**) Dot plot showing the mean expression (color scale) and the fraction of cells expressing each gene (dot size) of marker genes by macrophage subsets across the 12-organ

fetal atlas per organ. Data are min-max-standardized with a distribution of 0-1. The cell counts for each macrophage subset are displayed in the bar graphs.

# Supplementary Figure 9

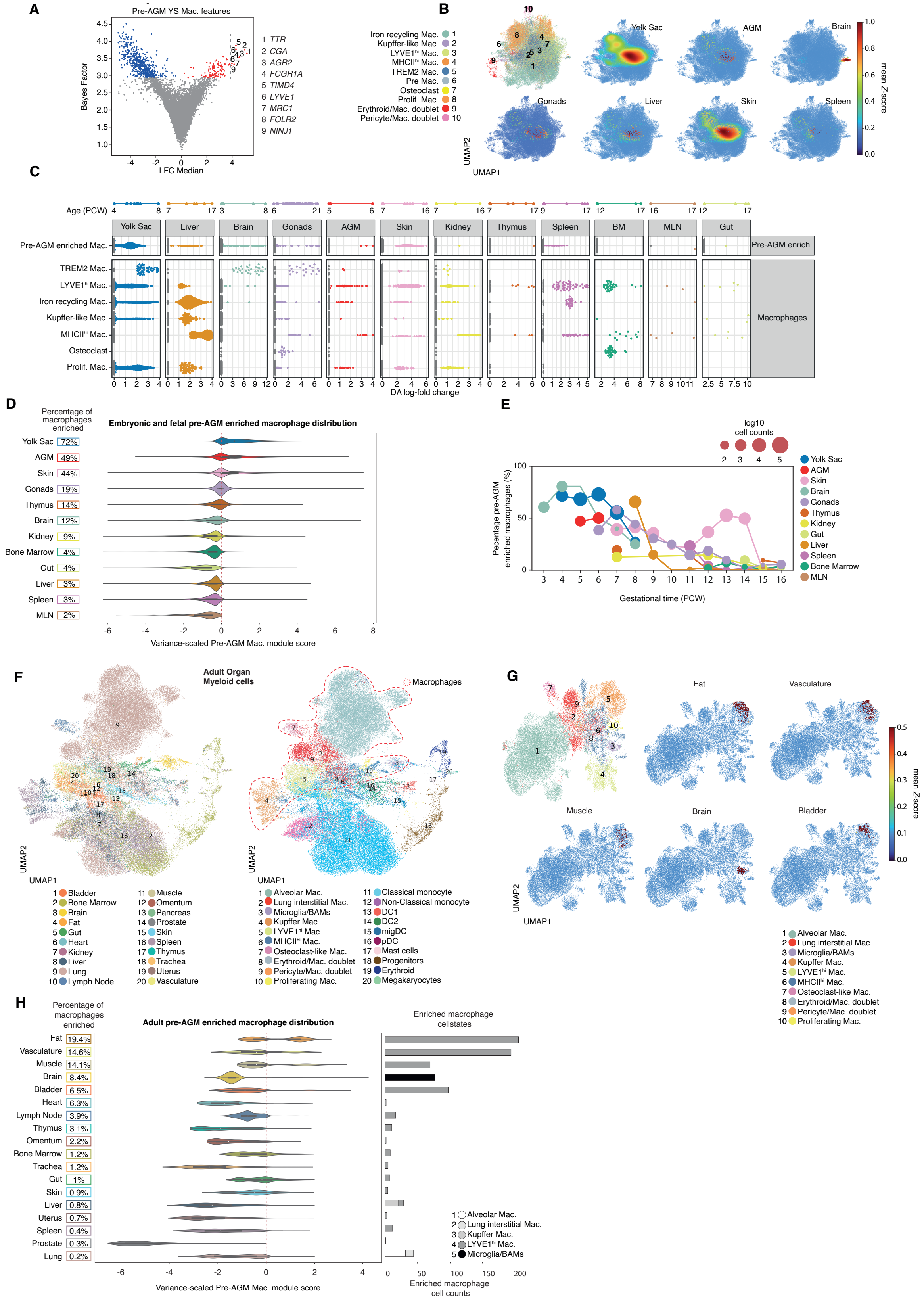

**Fig. S9. Pre-AGM macrophage gene signature in prenatal and adult organs.** (**A**) Volcano plot displaying the top nine significant differentially expressed genes (Bayes factor >3, Median LFC >4) of YS Macrophages arising pre-AGM (<CS14) against macrophages from all timepoints in the 12 organ-atlas, determined by *scVI* variational autoencoder differential feature selection (data S17). (**B**) UMAP (top left) and density plots showing macrophages from the 12-organ atlas colored by cell type (UMAP) or *z*-scored kernel density estimation (KDE) score of pre-AGM macrophage gene module enrichment (see methods), in AGM, brain, gonads, liver, skin, spleen and YS (Density plots). (**C**) Milo beeswarm plot showing neighborhood differential abundance of macrophages enriched in pre-AGM macrophage module, and other macrophage subtype modules across organs and gestational time. Colored neighborhoods are significantly enriched with positive fold changes (SpatialFDR<0.1, logFC>0) denoting significant presence at given time points and colors denote organs of origin (data S24). Per-organ sampled age ranges (PCW) are displayed at the top of the plot. (**D**) (left) Percentages of all macrophages positively enriched in Pre-AGM gene-module per organ in the fetal 12-organ atlas. (Middle) Violin plot showing distribution of the macrophages enriched in pre-AGM macrophage gene module. Violins show the median scaled module scores ((module_score-median)/MAD(module_score)). Each Violin indicates the distribution of enrichment scores across macrophages. The dotted red line indicates the threshold for positive enrichment (module_score>0) (data S7). (**E**) Line graph showing the relative change in proportion of macrophages enriched in the pre-AGM macrophage module across gestational age. Dot size represents log10 cell counts and color represents organ. (**F**) Left: Feature plot UMAP of the integrated 20-organ adult macrophage lineage atlas (n=65, k=94,286), colored by organ. Right: UMAP colored by annotated cell-states, red line indicates macrophage clusters. Organs include: bladder (n=3, k=2831), fat (n=2, k=1667), heart (n=7, k=486), pancreas (n=2, k=1030), omentum (n=4, k=103), prostate (n=2, k=356), thymus (n=3, k=508), vasculature (n=2, k=1602) (*77*), bone marrow (n=10, k=9408), gut (n=13, k=1678), (*78*), lung (n=19, k=46805), lymph node (n=,15 k=2507), muscle (n=13, k=2553), spleen (n=15, k=13643), skin (n=7, k=1088), trachea (n=6, k=1376), uterus (n=6, k=632), (*69*, *77*), brain (n=7, k=832)(*79*), kidney (n=9, k=358) (*80*), liver (n=12, k=4823) (*69*, *77*, *81*). (data S6 and S7). (**G**) UMAP (top left) and density plots showing macrophages from the adult atlas colored by cell type. UMAPs of the top five organs with the largest proportion of pre-AGM enriched macrophages colored by *z*-scored kernel density estimation (KDE) score of pre-AGM gene module enrichment (see methods). (**H**) (left) Percentages of all macrophages positively enriched in Pre-AGM gene-module per organ in the adult 20-organ atlas. (Middle) Violin plot showing distribution of the macrophages enriched in pre-AGM macrophage gene module. Violins show the median scaled module scores ((module_score-median)/MAD(module_score)). Each violin indicates the distribution of enrichment scores across macrophages. The dotted red line indicates the threshold for positive enrichment (module_score>0). (Right) Bar plot illustrating the cell-state distribution of positively enriched macrophage subsets per organ by percentage (data S7).

# Supplementary Figure 10

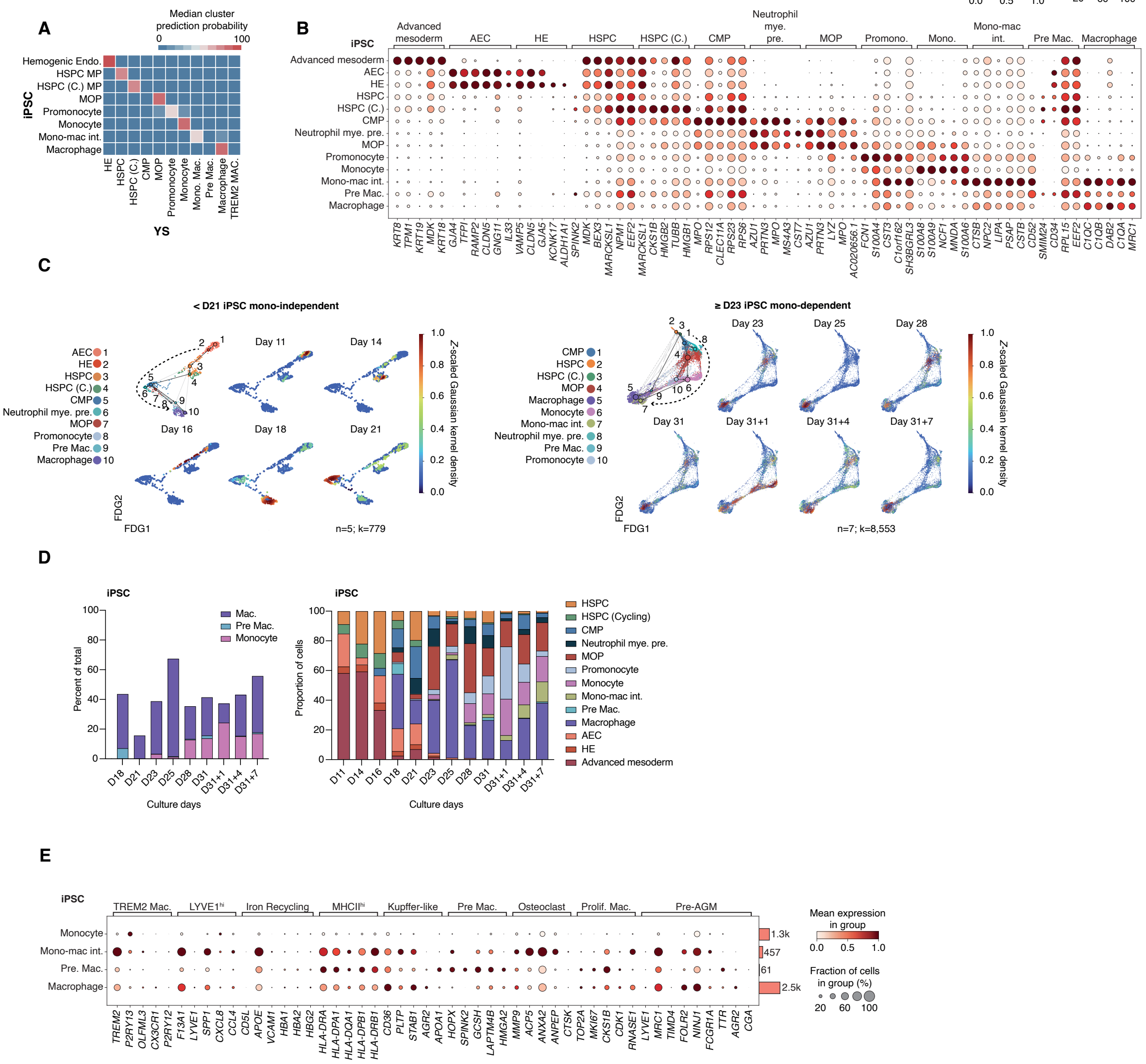

**Fig. S10. Accelerated macrophage differentiation in yolk sac and iPSC-derived macrophage cultures.** (**A**) Heatmap of class prediction probabilities for a logistic regression model (Elasticnet) trained on YS scRNA-seq myeloid cell states (*x*-axis) projected onto equivalent cell states in iPSC scRNA-seq data (*y*-axis), where the iPSC system was optimized for macrophage production (*20*). "Monocyte1" and "Monocyte2" are grouped into the "Monocyte" category (data S13). Color scale indicates median probabilities. (**B**) Dot plot showing the mean expression (color scale) and the fraction of cells expressing each gene (dot size) of myeloid lineage genes in myeloid-lineage cell states from the iPSC scRNA-seq (*20*) dataset (data S7). Data are min-max-standardized with a distribution of 0-1. (**C**) Density plots showing the distribution of transitioning iPSC-derived scRNA-seq (*20*) HSPCs and macrophage lineage cells from <D21 (n=5; k=779) (left) and >D23 (n=7; k=8553) (right) in the integrated FDG embedding from Fig. 5G. Color of cells represents the *z*-scored kernel density estimation (KDE) score for each timepoint (data S7 and S5). (**D**) Left: Stacked bar plot displaying the percent of monocytes, pre-macrophages, and macrophages found in iPSC cultures (*20*) by day. Right: Stacked bar plot displaying the proportion of cell states found in iPSC cultures by day. (**E**) Dot plot showing the mean expression (color scale) and the fraction of cells expressing each gene (dot size) of macrophage subtype-defining gene sets in iPSC macrophage cell states. Data are min-max-standardized with a distribution of 0-1 (data S17).

# Supplementary Figure 11

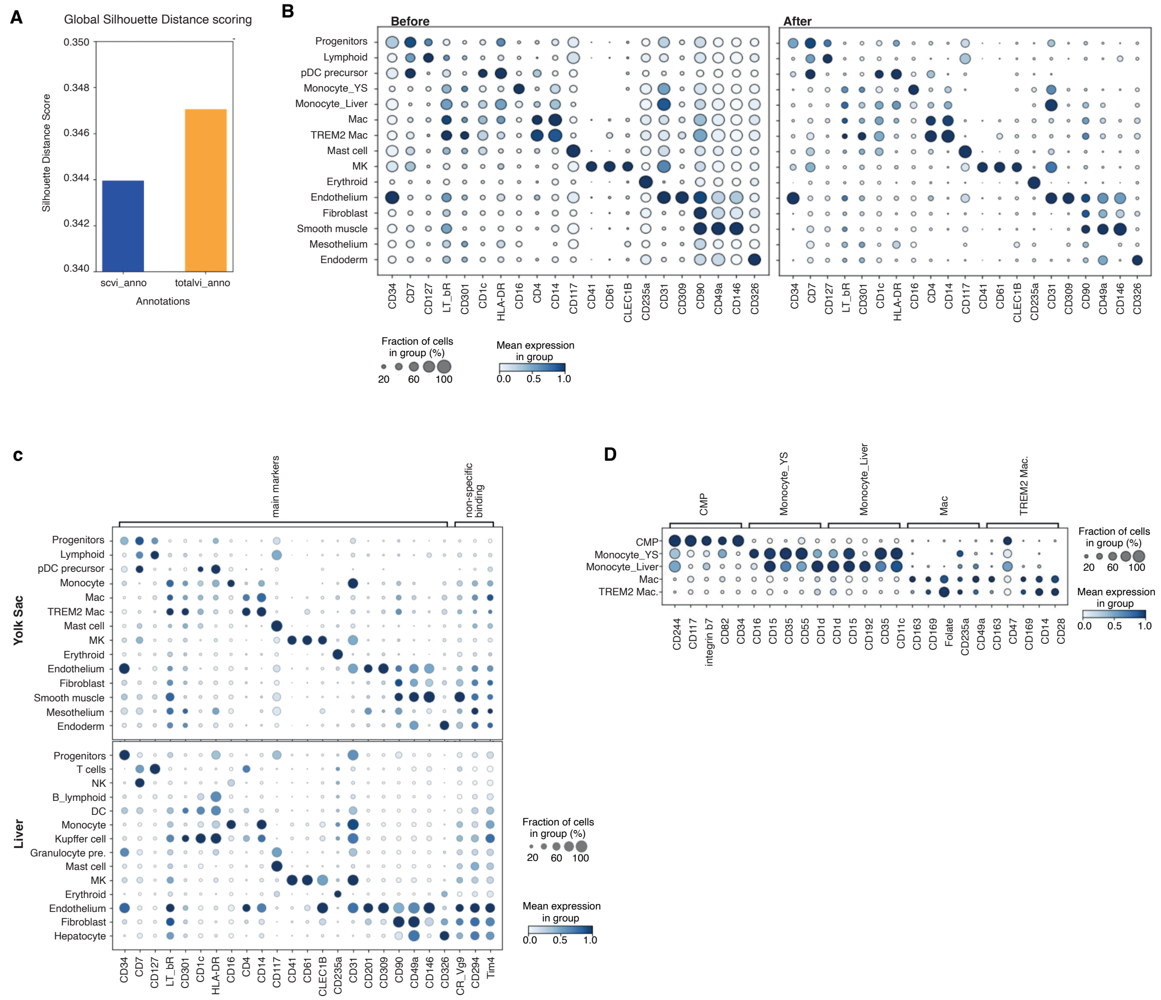

**Fig. S11. CITE-seq multi-modal integration and denoising.** (**A**) Bar chart displaying global silhouette distance scores on cluster-derived annotations between SCVI and totalVI annotations. A higher score indicates stronger independent cell cluster assignment. (**B**) Dot plots illustrating the mean expression (color scale) and the fraction of cells expressing each protein (dot size) of curated marker genes for cell-states using raw unprocessed YS CITE-seq data (left) and after DSB-GMM normalization following background noise removal (right). (**C**) Dot plot illustrating the mean expression (color scale) and the fraction of cells expressing each protein (dot size) of yolk sac (top) and liver (bottom) CITE-seq data showing discriminatory markers for cell states as well as three proteins that were identified as binding non-specifically but with highly variable expression. (**D**) Dot plot illustrating the mean expression (color scale) and the fraction of cells expressing each protein (dot size) of the top five differentially expressed proteins between myeloid cell states. Data are min-max-standardized with a distribution of 0-1.

# Supplementary Figure 12

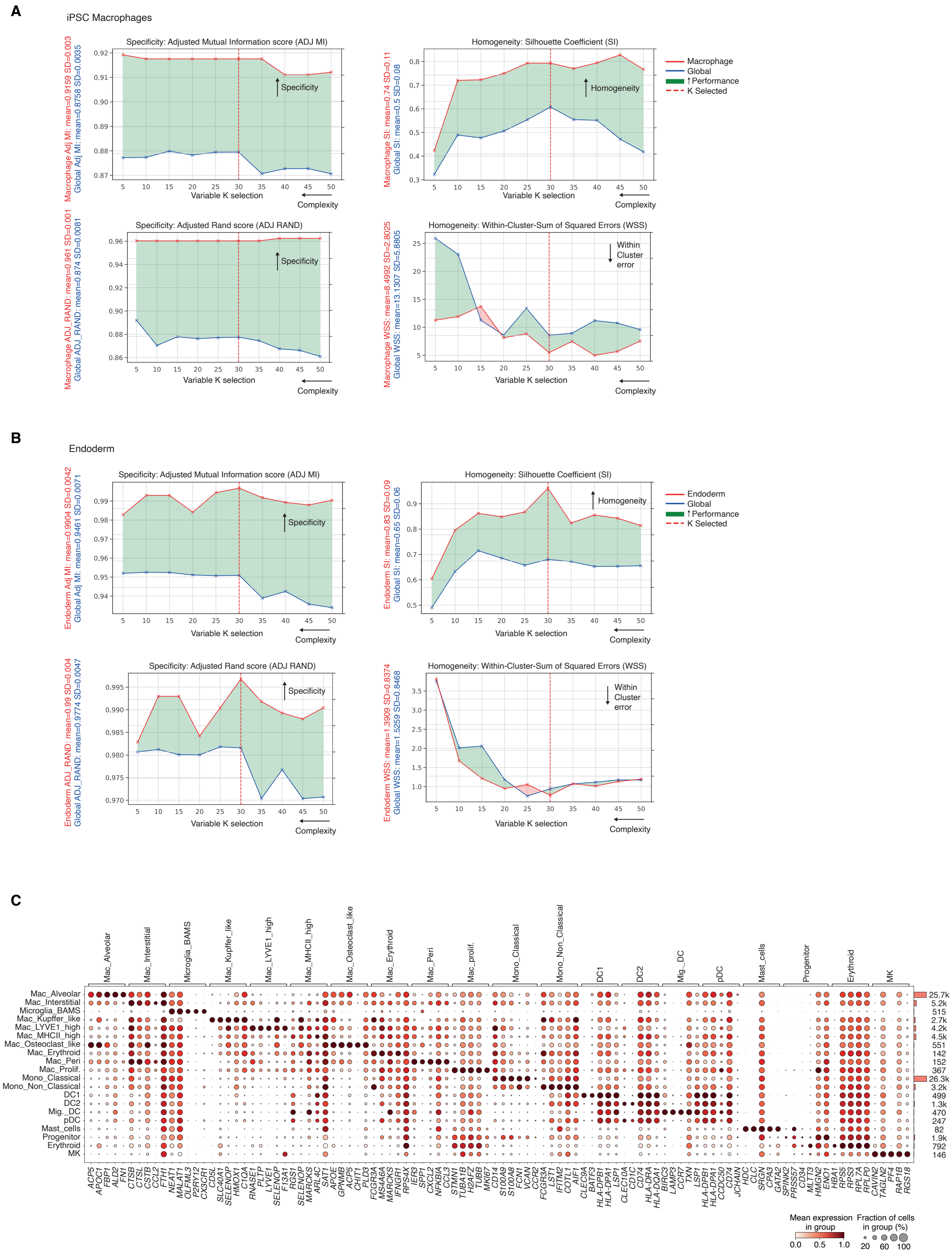

**Fig. S12. Prenatal, iPSC and adult myeloid and endoderm population annotation specificity.** (**A**) Left: Metrics of iPSC-derived macrophage population specificity, adjusted mutual information (MI) score (top) and adjusted rand (ADJ RAND) (bottom) of iPSC-derived macrophage population across decreasing graph complexity compared against all other populations. Right: Metrics of population homogeneity, silhouette coefficient index (SI) (top) and within squared sum error (WSS) (bottom) of iPSC-derived macrophage population across decreasing graph complexity (data S7). (**B**) Plots as described in **A** for the endoderm population compared against all other populations in the YS scRNA-seq data (data S3). (**C**) Dot plot showing the mean expression (color scale) and the fraction of cells expressing each gene (dot size) of significant differentially expressed markers ($P<0.05$, $LFC>0.25$) for each annotated cell state in the adult myeloid 20-organ atlas (data S7). Differential expression was derived via a two-sided Wilcoxon rank-sum test (thresholded at expression in >25% of class, $LFC > 0.25$ and Benjamini–Hochberg-corrected $P<0.05$) (data S17). Data are min-max-standardized with a distribution of 0-1.

# Supplementary Figure 13

**A**

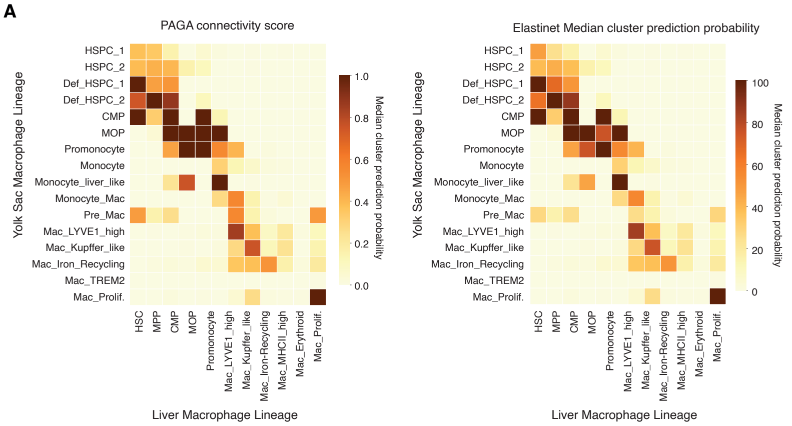


**B**

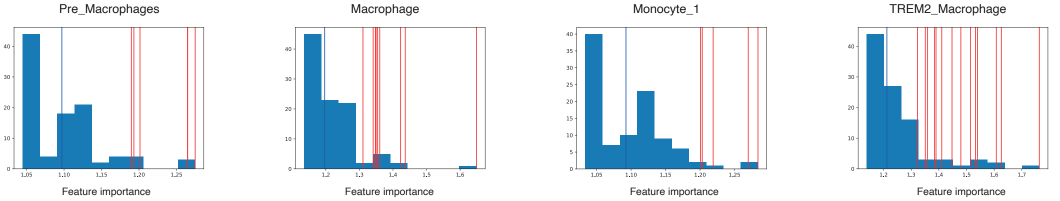


| | e^coef |
|---|---|
| *SMIM24* | 1.2748809 |
| *SPINK2* | 1.2649529 |
| *DAB2* | 1.2647697 |
| *CD14* | 1.2013143 |
| *CXCL8* | 1.1931766 |
| *C1QC* | 1.1897315 |

| | e^coef |
|---|---|
| *ACTB* | 1.64884 |
| *FCGRT* | 1.4364573 |
| *F13A1* | 1.422764 |
| *HLA-DPA1* | 1.3606718 |
| *CST3* | 1.3543245 |
| *C1QA* | 1.3496593 |
| *SPP1* | 1.3475064 |
| *C1QC* | 1.3408504 |
| *CDK1* | 1.3106023 |

| | e^coef |
|---|---|
| *HLA-DPA1* | 1.2840273 |
| *C15orf48* | 1.2698874 |
| *C5AR1* | 1.2190192 |
| *CLEC7A* | 1.2031921 |
| *LGALS3* | 1.2007608 |

| | e^coef |
|---|---|
| *CGA* | 1.7632571 |
| *APOC3* | 1.6258442 |
| *APOA1* | 1.6076742 |
| *TREM2* | 1.5397294 |
| *CX3CR1* | 1.5323834 |
| *APOA2* | 1.5140054 |
| *KRT8* | 1.4795908 |
| *APOM* | 1.4475342 |
| *OLFML3* | 1.41054 |
| *HSPB1* | 1.389693 |
| *P2RY13* | 1.384554 |
| *LY96* | 1.3593465 |
| *GATM* | 1.350822 |
| *VSIG4* | 1.3228374 |

**C**

Specificity of YS Macrophages annotation by DEGs

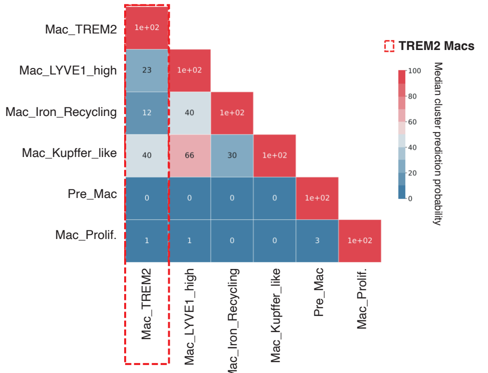


% overlap of top 100 DEGs across YS Macrophage subsets

**D**

Sensitivity of TREM2 Macrophage annotation by DEGs

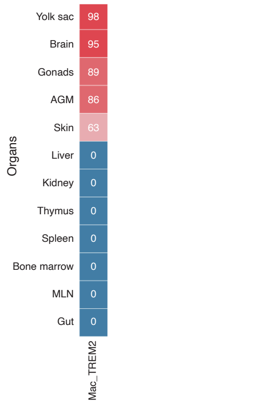


% overlap of top 100 DEGs across TREM2 Macrophages in pan-organ data

**Fig. S13. Probabilistic label transfer methodology and sensitivity in myeloid lineage populations across organs.** (**A**) Left: Heat map illustrating median PAGA connectivity scores based on abstracted neighborhood distances (AGA) between YS and corresponding liver macrophage cell-states. Right: ldVAE ElasticNet LR median cluster projection probabilities between YS labels and corresponding clusters in the fetal liver. The model is trained on the YS subset of a jointly integrated 12-organ ldVAE latent representation (C=0.2, L1_ratio=0.05, R2=0.86, RMSE=0.13). (data S14). (**B**) Histograms illustrating key discriminatory features predicted by logistic regression (top) between YS macrophage cell states weighed by impact on model prediction (e^Coeff). Red lines indicate positions of significant features (P<0.05 of 1-cumulative distribution function) in the histogram. (**C**) Heat map illustrating the number of intersecting genes within the top 100 differentially expressed genes (DEGs; P<0.05, LFC>0.25) for each macrophage subset in the YS, compared against DEGs identified for each of the other Macrophage subsets. DEGs were computed between macrophages in YS using the scanpy tl.rank_gene_groups function (see manuscript methods) with a threshold of P<0.05 and logfc>0.25. (data S3 and S31) (**D**) Heatmap illustrating percentage overlap of top 100 differentially expressed genes for TREM2 macrophages across the 12 developmental tissue cross-organ atlases compared against independently computed DEGs per-organ for TREM2 macrophages. DEGs were computed between macrophage types across organs using the scanpy tl.rank_gene_groups function with a threshold of P<0.05 and logfc>0.25.

# Supplementary Figure 14

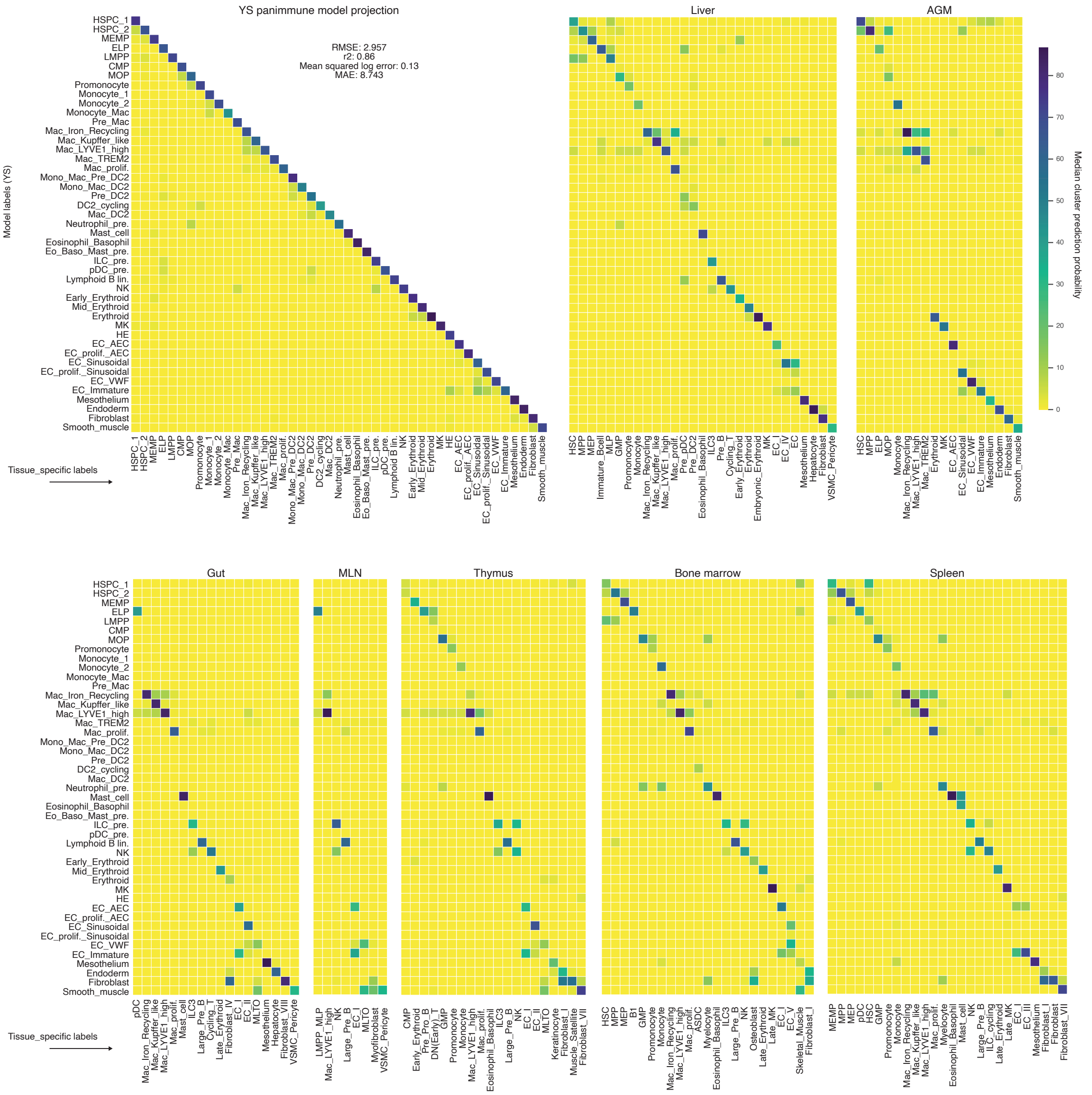

**Fig. S14. Logistic regression model of the fetal pan-organ variational landscape.** Heat maps illustrating ldVAE ElasticNet LR median cluster projection probabilities between YS labels (y-axis) and corresponding clusters (x-axis) in eight of the 12-organ fetal atlas (YS, liver, AGM, gut, MLN, thymus, FBM and spleen). The model is trained on the YS subset of a jointly integrated 12-organ ldVAE latent representation (C=0.2, L1_ratio=0.05, R2=0.86, RMSLE=0.13). Only cell types which had a max probability >0.05 for any YS label are shown in the heatmap (data S6, S7 and S14).

# Supplementary Figure 15

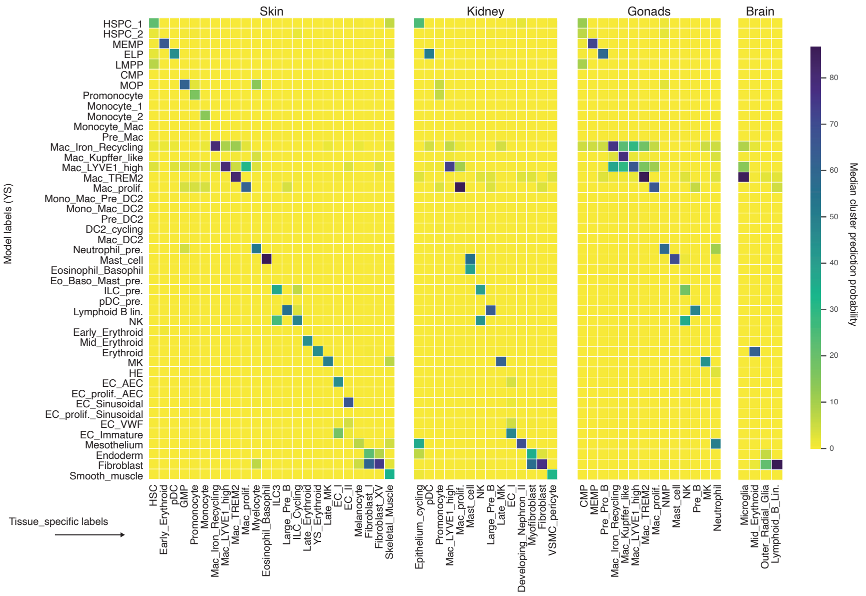

**Fig. S15. Logistic regression model of the fetal pan-organ variational landscape continued.** Heat maps illustrating ldVAE ElasticNet LR median cluster projection probabilities between YS labels (y-axis) and corresponding clusters (x-axis) in four of the 12-organ fetal atlas (skin, kidney, gonads, and brain). The model is trained on the YS subset of a jointly integrated 12-organ ldVAE latent representation (C=0.2, L1_ratio=0.05, R2=0.86, RMSLE=0.13). Only cell types which had a max probability >0.05 for any YS label are shown in the heatmap (data S6, S7 and S14).

**Movie S1. (separate file)**
Light-sheet fluorescence microscopy video of ~6.9-PCW YS stained with CD34 (yellow), HNF4A (cyan), and LYVE1 (magenta). Still taken for the left panel of Fig. 1E.

**Movie S2. (separate file)**
Light-sheet fluorescence microscopy video of ~7-PCW YS stained with PLVAP (green), LYVE1 (red), and IBA1 (white). Stills taken for fig. S3F.

**Data S1. (separate file)**

**Sample metadata.** The sample information metadata for the core YS and embryonic liver single cell datasets (scRNA-seq, CITE-seq and Smart-seq2) that have been integrated and analyzed for this study. Sample metadata includes, among other categories, sample ID, age, sex, tissue, sequencing platform, and alignment software. Data that we have previously together with our collaborators is noted clearly in the dataset column. All data included are made publicly available on ArrayExpress at the accessions noted, inclusive of raw FASTQ files and raw count matrices.

**Data S2. (separate file)**

**Sample Manifest.** The sample manifest directly follows the sample metadata table. This manifest includes summaries for the YS and embryonic liver scRNA-seq, CITE-seq, and Smart-seq2 data that have been integrated and analyzed for this study. Metadata and cell count information are provided more broadly for each biological replicate and tissue.

**Data S3. (separate file)**

**YS scRNA-seq (10X) metadata.** Metadata for the integrated YS 10X scRNA-seq data including scrublet QC calls by barcode.

**Data S4. (separate file)**

**YS and liver scRNA-seq and CITE-seq (10X) cell state counts.** Cell state annotation counts in each of the core yolk sac and embryonic liver single cell datasets (scRNA-seq, CITE-seq and Smart-seq2) desegregated by biological replicate.

**Data S5. (separate file)**

**Coordinates metadata.** Dimensional reduction coordinates by cell barcode for all UMAP and FDG embeddings shown in this study's main and supplementary figures.

**Data S6. (separate file)**

**External datasets.** A table detailing all external single-cell datasets leveraged for comparison with our core human yolk sac and embryonic liver single cell datasets. For each external dataset, we note the organ, research article DOI, data accessibility links, and number of biological replicates and cells. We also provide notes for any selection criterion where appropriate.

**Data S7. (separate file)**

**External dataset annotations.** Barcodes cell annotations for all external datasets used, which are present within both main and extended figure panels shown. Data for fetal and adult atlases also include per cell Pre-agm enrichment calls.

**Data S8. (separate file)**

**Logistic Regression YS scRNA-seq to YS ss2 scRNA-seq**. Table output from Logistic regression ElasticNet model trained on the combined low-dimensional representation of the YS scRNA-seq data for purposes of projection, class assignment and visualization of class correspondence between trained labels and target YS SS2 scRNA-seq plate-based data annotations. (See Methods for "cell state predictions using probabilistic low-dimensional ElasticNet regression" for default parameters used.)

**Data S9. (separate file)**

**Logistic Regression YS scRNA-seq to YS CITE-seq.** Table output from the Logistic regression l2 model (sparsity=0.2, max_iter=1000) trained on the combined low-dimensional representation of the YS scRNA-seq data for purposes of projection, class assignment, and visualization of class correspondence between trained labels and target YS Cite-seq RNA data annotations. (See Methods for "Cell state predictions using probabilistic low-dimensional ElasticNet regression" for default parameters used.)

**Data S10. (separate file)**

**Logistic Regression EL scRNA-seq to Liver CITE-seq**. Table output from the Logistic regression ElasticNet model trained on the combined low-dimensional representation of the EL scRNA-seq data for purposes of projection, class assignment and visualization of class correspondence between trained labels and target YS scRNA-seq data annotations. (See Methods for "Cell state predictions using probabilistic low-dimensional ElasticNet regression" for default parameters used.)

**Data S11. (separate file)**

**Logistic Regression YS scRNA-seq to mouse yolk sac scRNA-seq**. Table output from the Logistic regression ElasticNet model trained on the combined cross-species integrated low-dimensional representation of the YS scRNA-seq data for purposes of projection, class assignment, and visualization of class correspondence between trained labels and target mouse scRNA-seq data clusters (See Methods for "Cell state predictions using probabilistic low-dimensional ElasticNet regression" parameters).

**Data S12. (separate file)**

**Logistic Regression YS scRNA-seq to EL scRNA-seq.** Table output from the Logistic regression ElasticNet model trained on the expression data of the YS scRNA-seq data for purposes of projection, class assignment, and visualization of class correspondence between trained labels and target EL RNA data annotations. (See Methods for "Cell state predictions using probabilistic low-dimensional ElasticNet regression" for default parameters used.)

**Data S13. (separate file)**

**Logistic Regression YS scRNA-seq to iPSC scRNA-seq**. Table output from the Logistic regression ElasticNet model trained on the expression data of the YS scRNA-seq data for purposes of projection, class assignment and visualization of class correspondence between trained labels and target iPSC scRNA-seq data annotations. (See Methods for "cell state predictions using probabilistic low-dimensional ElasticNet regression" for default parameters used.)

**Data S14. (separate file)**

**Logistic Regression YS scRNA-seq to AGM, EL and FBM endothelium and across the integrated fetal atlas.** Table output from the Logistic regression ElasticNet model trained on the combined low-dimensional representation of the YS scRNA-seq data for purposes of projection, class assignment, and visualization of class correspondence between trained labels and target datasets across the fetal atlas and AGM, EL, and FBM scRNA-seq data annotations. (See Methods for "Cell state predictions using probabilistic low-dimensional ElasticNet regression" for default parameters used.)

**Data S15. (separate file)**

**Logistic Regression projection probabilities for YS Gastrulation SS2 data**. Table output from the Logistic regression ElasticNet model trained on the combined low-dimensional representation of the YS scRNA-seq data for purposes of projection, class assignment, and visualization of class correspondence between trained labels and target YS gastrulation SS2 data annotations. (See Methods for "Cell state predictions using probabilistic low-dimensional ElasticNet regression" for default parameters used.)

**Data S16. (separate file)**

**Logistic Regression YS scRNA-seq to mouse hematopoietic lineage.** Table output from the Logistic regression ElasticNet model trained on the combined low-dimensional representation of the cross species SAMAP sequence-blast YS scRNA-seq data for purposes of projection, class assignment, and visualization of class correspondence between trained labels and target mouse hematopoietic lineage scRNA-seq data annotations. (See Methods for "cell state predictions using probabilistic low-dimensional ElasticNet regression" for default parameters used.)

**Data S17. (separate file)**

**Differentially expressed genes for scRNA-seq datasets.** Differentially expression testing results from independent analyses between cell states in the YS, EL and IPSC scRNA-seq datasets derived from the sc.tl.rank_gene_groups function in the Scanpy package which performed a two-sided Wilcoxon rank-sum test. DEGs were expressed in >25% of cells and had a log-transformed fold change of >0.25. All *P*-values were adjusted for multiple testing using the Benjamini–Hochberg method and the threshold for statistical significance was set as $P<0.05$.

**Data S18. (separate file)**

**Smart-Seq2 Metadata.** Metadata for the yolk sac Smart-seq2 data by barcode.

**Data S19. (separate file)**

**Yolk sac CITE-seq metadata.** Metadata for the yolk sac CITE-seq data by barcode for RNA and protein.

**Data S20. (separate file)**

**Differentially expressed proteins in yolk sac CITE-seq all-cell states and myeloid cell states.** Marker proteins were derived using a one-vs-all TotalVI differential expression test ($P<0.05$, Bayes factor >0.95, Median LFC > 0.5) or Wilcoxon rank sum test using sc.tl.rank_genes_groups function from scanpy (more details on this approach are available in Methods under "Clustering and annotation of scRNA-seq and CITE-seq data").

**Data S21. (separate file)**

**Embryonic liver (EL) scRNA-seq (10X) metadata**. Metadata for the embryonic liver 10X scRNA-seq data by barcode.

**Data S22. (separate file)**

**Yolk sac and embryonic liver CITE-seq metadata**. Metadata for the Liver CITE-seq data by barcode for RNA and protein.

**Data S23. (separate file)**
**Antibody list.** Antibodies used for cell sorting and imaging: (i) enrichment sort for scRNA-seq (10X, CITE-seq and SS2), (ii) IHC, (iii) FFPE immunofluorescence microscopy, (iv) fixed frozen immunofluorescence microscopy, (v) light-sheet fluorescence microscopy, and (vi) RNAscope-associated immunofluorescence microscopy

**Data S24. (separate file)**
**MILO differential abundance analysis output**. Differential abundance of cell-state neighborhoods by MILO with differential expression testing results across each cell state enriched in early and late neighborhoods. The statistical significance of differential abundance was set as SpatialFDR (<0.1, logFC <0) for early enriched neighborhoods and SpatialFDR (<0.1, logFC >0) for later enriched neighborhoods. (See Methods for "differential abundance testing and FACs correction").

**Data S25. (separate file)**
**Mouse gene knockout lethality and conserved and differential clustered enriched gene-set module statistics between mouse and human endoderm scRNA-seq (10X).** Neighborhood clusters and statistical significance of significantly enriched gene set modules for differential and conserved expression between cell states of interest (See Methods for "Clustered gene-set enrichment analysis").

**Data S26. (separate file)**
**Differentially expressed genes in YS scRNA-seq endoderm across time from MILO derived neighborhoods and GSEA enrichment statistics**. Differentially expression testing results from independent analyses between endoderm across time using MILO derived neighborhoods derived from the sc.tl.rank_gene_groups function in the Scanpy package which performed a two-sided Wilcoxon rank-sum test. The threshold for statistical significance was set as $P<0.05$. Gene set enrichment outputs produced by the enrichr (P values provided from Fisher exact test and ranked by *z*-score) workflow as implemented in the GSEApy package (See Methods for "Differential abundance testing and FACS correction" and "Clustering and annotation of scRNA-seq and CITE-seq data" for default differential abundance and differential expression testing parameters).

**Data S27. (separate file)**
**Differentially expressed proteins in YS CITE-seq early and definitive HSPCs**. Differential expression testing results from independent analyses between HSPC subsets in the YS CITE-seq dataset derived using a one-vs-all TotalVI differential expression test (Bayes factor >0.5) (See Methods under "Clustering and annotation of scRNA-seq and CITE-seq data").

**Data S28. (separate file)**
**CellphoneDB receptor–ligand interactions statistics across YS scRNA-seq cellstates data (10X).** Significant CellPhoneDB receptor ligand interaction predictions between all cell states in the YS scRNA-seq data run using the CellPhoneDB statistical method workflow desegregated by gestational time (see Methods for "cell–cell interaction predictions using CellPhoneDB"). The threshold for statistical significance of mean receptor ligand expression was set as $P<0.05$.

**Data S29. (separate file)**

**CellphoneDB receptor–ligand interactions statistics between HSPCs and stroma in YS, EL and AGM scRNA-seq cell-state data (10X).** Significant CellPhoneDB receptor ligand interaction predictions between HSPC cellstates and stroma in the YS, EL, and AGM scRNA-seq data desegregated by gestational time ran using the CellPhoneDB statistical method workflow (see Methods for "cell–cell interaction predictions using CellPhoneDB"). The threshold for statistical significance for mean receptor ligand expression was set as $P<0.05$.

**Data S30 (separate file)**

**pyScenic differential regulon usage statistics**. Differential regulon usage across the YS scRNA-seq macrophage differentiation pseudotime trajectory. Significantly changing regulons across pseudotime were estimated by a general additive model (See Methods for pyScenic) ($P< 0.05$). Significant GAMs with a logFC±0 for each cell state were retained for ranking in visualization.

**Data S31. (separate file)**

**Differentially expressed genes for myeloid cell states across the 12 organ integrated fetal atlas scRNA-seq datasets**. Differential expression testing results from independent analyses between cell states in the integrated fetal atlas scRNA-seq datasets derived from the sc.tl.rank_gene_groups function in the Scanpy package which performed a two-sided Wilcoxon rank-sum test for genes expressed in >25% of cells, log-transformed fold-change cut-off of 0.25. All *P*-values were adjusted for multiple testing using the Benjamini–Hochberg method and the threshold for statistical significance was set as $P<0.05$.

**Data S32. (separate file)**

**MILO differential abundance analysis across fetal organ output**. Differential abundance of cell-state neighborhoods by MILO with differential expression testing results across each cell state enriched in early and late neighborhoods by organ. The threshold for statistical significance for differential abundance was set as SpatialFDR (<0.1, logFC <0) for early enriched neighborhoods and SpatialFDR (<0.1, logFC >0) for later enriched neighborhoods. (See Methods for "differential abundance testing and FACs correction").

**Data S33. (separate file)**

**CITE-seq antibodies.** Detailed information for the TotalSeq™ (Biolegend) antibodies used, including target, clone, and barcode, in the CITE-seq cocktail used on embryonic YS and liver.
